# The Role of Light in Mediating Physiological Stress in Small Corals Exposed to Elevated Temperature

**DOI:** 10.1002/ece3.74505

**Published:** 2026-11-12

**Authors:** P. J. Edmunds, K. C. Scafidi, F. Cohn, K. Layagala, J. A. Sims, A. M. S. Correa, S. C. Burgess

**Affiliations:** ^1^ Department of Biology California State University Northridge California USA; ^2^ Department of Marine Science University of Otago Dunedin Otago New Zealand; ^3^ Department of Environmental Science, Policy, and Management University of California, Berkeley Berkeley California USA; ^4^ Department of Biological Science Florida State University Tallahassee Florida USA; ^5^ Department of Biology Norwegian University of Science and Technology Trondheim Norway

**Keywords:** bleaching, coral reef, *Pocillopora*, Scleractinia, thermal tolerance

## Abstract

Marine heat waves (MHW) are important stressors on coral reefs, and evaluating their implications for the population dynamics of young corals has become a key research need. However, projecting the effects of MHW on the demographics of young corals is made difficult by an incomplete understanding of how their response to high temperature is modulated by light. Here we tested for the interactive effects of temperature and light on genetically identified colonies of the Pacific coral, *Pocillopora* spp. from 3‐m depth in the back reef of Moorea, French Polynesia. The ecological implications of the responses to manipulated temperature and light were strengthened through context provided by long‐term in situ records of temperature and light. Two 14‐day trials were conducted in which young *P. meandrina* were exposed to 28°C and 31°C at six light levels ranging from 436 to 1621 μmol photons m^−2^ s^−1^. The probability of bleaching was unaffected by temperature, but it increased across the light gradient, while the probability of partial or full mortality increased with light faster at 31°C compared to 28°C. Mortality was associated with a decline in the photophysiological performance (*F*_v_/*F*_m_) of the symbiotic algae (Symbiodiniaceae) in the corals, notably as light levels increased at 31°C but not at 28°C, and at light levels commonly reached at 3‐m depth. Declines in *F*_v_/*F*_m_ revealed impaired PSII functionality, although values remained higher than those usually associated with bleaching (i.e., < 0.500). These results show how light can modulate the impacts of MHWs on the physiology and demography (i.e., survival) of young *Pocillopora* spp., and show that corals pale enough to be scored as fully bleached on the reef can contain relict algal populations that remain photophysiologically functional at elevated temperature and high light.

## Graphical abstract

This research describes how naturally occurring variation in underwater light intensity can quantitatively alter the susceptibility of small and young corals to thermal bleaching. Codification and quantification of the light‐bleaching and light‐mortality relationships advance the understanding of the foundational biology of bleaching and will also improve the capacity to predict the occurrence and severity of bleaching at high temperature.

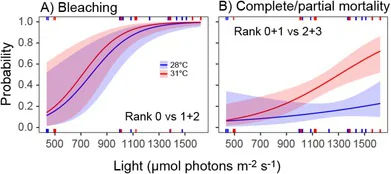

## 1 Introduction

The sensitivity of corals to high temperature is a defining feature of tropical reefs (Couce et al. 2012; Yonge 1968) that restricts their distribution to equatorial latitudes (Muir et al. 2015; Stoddart 1969). The effects of temperature have therefore been studied since the earliest physiological experiments were conducted with corals (Mayer 1917; Edmondson 1928), although it was not until the 1970s that their narrow thermal tolerance was rigorously quantified (Coles et al. 1976; Jokiel and Coles 1977). In the 1980s, the thermal biology of corals became a focus of research because the first large‐scale coral bleaching episodes (Lasker et al. 1984; Roberts 1987) were quickly associated with elevated seawater temperature (Glynn 1993).

Bleaching is a general physiological stress response of symbiotic corals, and when it persists for weeks, affected corals are likely to die (Brown 1997; Lesser 2011; Lough and van Oppen 2018). The large spatial extent and severity of these events (Reimer et al. 2024; Sully et al. 2019) have made bleaching a centerpiece of coral reef science (Hughes et al. 2017), with this trend intensifying as marine heat waves (hereafter, MHW, Oliver et al. 2021) in the 21st Century have amplified coral mortality through bleaching (Hughes et al. 2017, 2018). Although research has identified multiple proximal mechanisms of thermal bleaching (Helgoe et al. 2024), some of the greatest attention has been accorded to the interactive effects of elevated temperature and bright light (Helgoe et al. 2024; Lesser et al. 1990). These effects are caused by the production of superoxide radicals that damage cell functionality (Helgoe et al. 2024; Lesser 2006).

Corals are thus less likely to bleach at high temperatures if light levels are low (Anthony et al. 2007; Mumby et al. 2001; Tagliafico et al. 2022). The severity of bleaching can also be reduced if acclimatization or adaptation to elevated temperature has occurred (Baker et al. 2004; Coles and Brown 2003), which are possibilities supported by the results of manipulative experiments involving temperature (Humanes et al. 2022; Johnston et al. 2024), as well as studies of multiple bleaching events affecting the same coral communities (Lachs et al. 2023). While protection from high temperature by low light creates opportunities for the survival of corals in shady habitats during periods of high temperature, it does not alter their thermal sensitivity and leaves them vulnerable to future MHWs. In contrast, acclimation and adaptation can fundamentally alter thermal sensitivity to leave corals less vulnerable to MHWs, but these processes are likely to require substantial time to occur. It is important to distinguish among acclimatization, adaptation, and attenuation of thermal stress through synergy with other factors, particularly for small and young corals. This size class and life stage has been less extensively studied than adult corals or their fragments (McLachlan et al. 2020), even though they have positive demographic influences on population growth (Doropoulos et al. 2016; Speare et al. 2025). One means of modulating this influence is through a recruitment bottleneck (Doropoulos et al. 2016; Edmunds 2021) that controls the number of corals advancing to sexually mature adult life stages.

Despite the well‐known importance of light in coral bleaching (Helgoe et al. 2024; Lesser et al. 1990), and more broadly to phototrophic reef corals (Dubinsky and Falkowski 2010), light has rarely been continuously recorded underwater on coral reefs (Hochberg et al. 2020; Yentsch et al. 2002). Our measurements of light on coral reefs show large variation over multiple time scales at 16‐m depth in St. John, U.S. Virgin Islands. In St. John, the mean molar extinction coefficient (*K*_d_) differs 63% between September and November, and light integrated by day varies 4‐fold between days and can be depressed for weeks following storms (Edmunds et al. 2018, 2019). Variation in underwater light is driven by reflectance at the sea surface, absorption and scattering by seawater (Hochberg et al. 2020; Kirk 2011), and absorbance by benthic surfaces (Mozel 2001), but it is also affected by surface light, which changes dynamically with cloud cover, weather, and season. Additionally, surface light is also affected by “global dimming” (Wild 2009), which is attributed to a variety of mechanisms of which high altitude aerosols and clouds are most prominent (Stamatis et al. 2024; Wild 2009), with comparable effects reported as “darkening” of global oceans (Davies and Smyth 2025). Given the multiple causes of variation in underwater light, and the growing threats to corals of rising temperatures (Hoegh‐Guldberg et al. 2023), it is timely to revisit the positive synergy between light and temperature (Helgoe et al. 2024) to codify and quantify the relationship between bleaching and light at high temperature.

Using small colonies of six *Pocillopora* species from the back reef of Moorea, French Polynesia, we tested the hypothesis that the susceptibility of corals to elevated temperature is quantitatively modulated by light, with susceptibility evaluated by photophysiological performance, bleaching, and mortality. We focused on small and young corals to better understand how temperature mediates the stringency of the recruitment bottleneck that influences population growth (Doropoulos et al. 2020; Edmunds 2021; Speare et al. 2025). Colonies of *Pocillopora* spp. that were ≲ 40 mm diameter were considered small, and assumed to be young (i.e., < 1–2 year old (Nozawa et al. 2021; Speare et al. 2025)) with genetically distinct host tissue, because they originated from the settlement of pelagic larvae of sexual origin. Because *Pocillopora* contains multiple cryptic species with distinct phenotypes throughout the Indo‐Pacific (Johnston et al. 2022), the corals used in our experiment were genetically identified (Cohn et al. 2026a, 2026b). The primary analyses focus on the responses of the most common species, and the results for all species (i.e., pooled as *Pocillopora* spp.) are presented in Supporting Information. By exploiting an abundant early life stage with an annual survivorship of ca. 70% (Kodera et al. 2020; Nozawa et al. 2021; Speare et al. 2025), our collecting permit allowed enough corals to be collected to use mortality as a response variable. This approach strengthened the capacity to evoke fitness‐related consequences of our results and reduced the demographic implications of collecting corals (because ~60% would have died on the reef in 2 years).

## 2 Materials and Methods

### 2.1 Overview

The experiment was completed in April and May 2025 using *Pocillopora* spp. from the back reef (~3‐m depth) of the eastern shore of Moorea (−15.519° S, −149.764° W). Two 14‐day trials were completed, each with new corals, and both employed six tanks (150 L) at ~28°C and six at ~31°C. Each tank at a given temperature was maintained at different light levels (photon flux densities [PFD] of photosynthetically active radiation [PAR, 400–700 nm]) using AquaIllumination Hydra 64HD LED lights operated at 30%, 60%, 70%, 80%, 90%, or 100% of their power output, and was stocked with 18 corals (for 468 corals across both trials). The vagaries of each LED light resulted in the percentage power settings providing precise but variable PAR levels. Therefore, PAR was recorded in all tanks throughout the experiment and mean PAR levels was used as a continuously distributed independent variable in the statistical design. The response of the corals was evaluated through the photophysiological performance of PSII and “health” (on a nominal scale culminating with death). The response variables were compared among temperatures, light levels, time, and trials using generalized linear mixed effects models (GLMM) for the most abundant species and also for *Pocillopora* spp. (i.e., pooled among taxa). Long‐term records of surface light, underwater light, and seawater temperature were used to provide context to the treatment conditions.

### 2.2 Light and Temperature

The quantity of light (i.e., PAR) was measured on the surface and underwater on the fore reef using cosine corrected logging meters. Surface light was measured on the roof of the lab (~3‐m above sea level) adjacent to PaoPao (Cook's) Bay using duplicate weather stations (Hobo USB Micro, H21‐USB, Hobo Data Loggers, Bourne, MA) fitted with a PAR sensor (S‐LIAM003, Hobo Data Loggers, Bourne, MA) and logging at 0.0017 Hz. Underwater PAR was recorded at 17‐m depth using a logger (MiniPAR, PME, Vista, CA) fitted with a cosine correct sensor (LI‐192, LiCor Environmental, Lincoln, NE) and wiper (MiniWiper, PME, Vista, CA) and recording at 0.0017 Hz. Records of surface and underwater light from 19 May 2021 to 29 April 2025 were used to calculate the molar extinction coefficient for PAR (*K*_dPAR_; Kirk 2011), which was used to estimate light at the collection depth of the corals. As the corals were collected just behind the reef crest, we assumed that the optical properties of the seawater at the collection site were the same as seawater on the fore reef and therefore light at 3‐m depth could accurately be predicted using *K*_dPAR_ (Kirk 2011). Light was also integrated by day (mol photons m^−2^ day^−1^) at the surface and at 3‐m depth. Seawater temperature was recorded at 10‐m depth on the north shore fore reef (LTER2 site) using an SBE39 logger (0.0083 Hz and ±0.002°C), with data from 19 May 2021 to 29 April 2025 reported.

### 2.3 Coral Collection

Corals were collected on snorkel three days before each trial on 4 April (Trial 1) and 20 April (Trial 2). Collections targeted colonies ≲ 40‐mm diameter on carbonate rock, ~75 m behind the reef crest, and all were discrete growths and not part of an antecedent *Pocillopora* skeleton. Trial 1 started on 7 April and finished on 21 April, and Trial 2 started on 23 April and finished on 7 May.

For each trial, 234 corals were collected with a hammer and chisel, individually bagged, and returned to the lab for processing. Sampling took ~4 person‐hours and the number of corals collected represented a small proportion of the young *Pocillopora* spp. growing in this location. Freshly collected corals were measured with calipers (mean of two planar diameters, ±1 mm); 18 were randomly selected for biomass determinations, and 216 were prepared as nubbins (Birkeland 1976) and were swabbed using sterile polyester swabs (PurFlock Ultra, Puritan Medical Products, Guilford, MA). Swab tips were then excised into 2 mL cryovials containing a DNA/RNA stabilizing solution (Zymo DNA/RNA shield, Zymo Research, Irvine, CA) and stored at 4°C until used subsequently for analysis.

Processing of the freshly collected corals took place in a shallow tank filled with seawater from PaoPao (Cook's) Bay and exposed to sunlight screened by an opaque acrylic roof. Colonies were glued upright onto numbered plastic tiles (~4 × 4 cm) using cyanoacrylate adhesive (Coral Glue, Ecotech Elements) and transferred on the day of collection to a 1000 L tank for acclimation. This tank was maintained at 29°C using a heater and chiller, supplied with seawater from PaoPao (Cook's) Bay and filtered through sand (~100 μm pore size), and illuminated with four LED lights (AquaIllumination Hydra 64HD). The lights supplied a mean (±SE) maximum light of 1316 ± 62 μmol photons m^−2^ s^−1^ (*n* = 11 records) with 4 h ramping to provide ~38 mol photons m^−2^day^−1^. The temperature and light in the tank were close to ambient in situ conditions at the time of coral collection from the study site (Figure 1). The corals were placed in the 1000 L tank on a platform that rotated at 2 revolutions day^−1^ (to remove position effects) and were kept under these conditions for 3 days.

**FIGURE 1:**
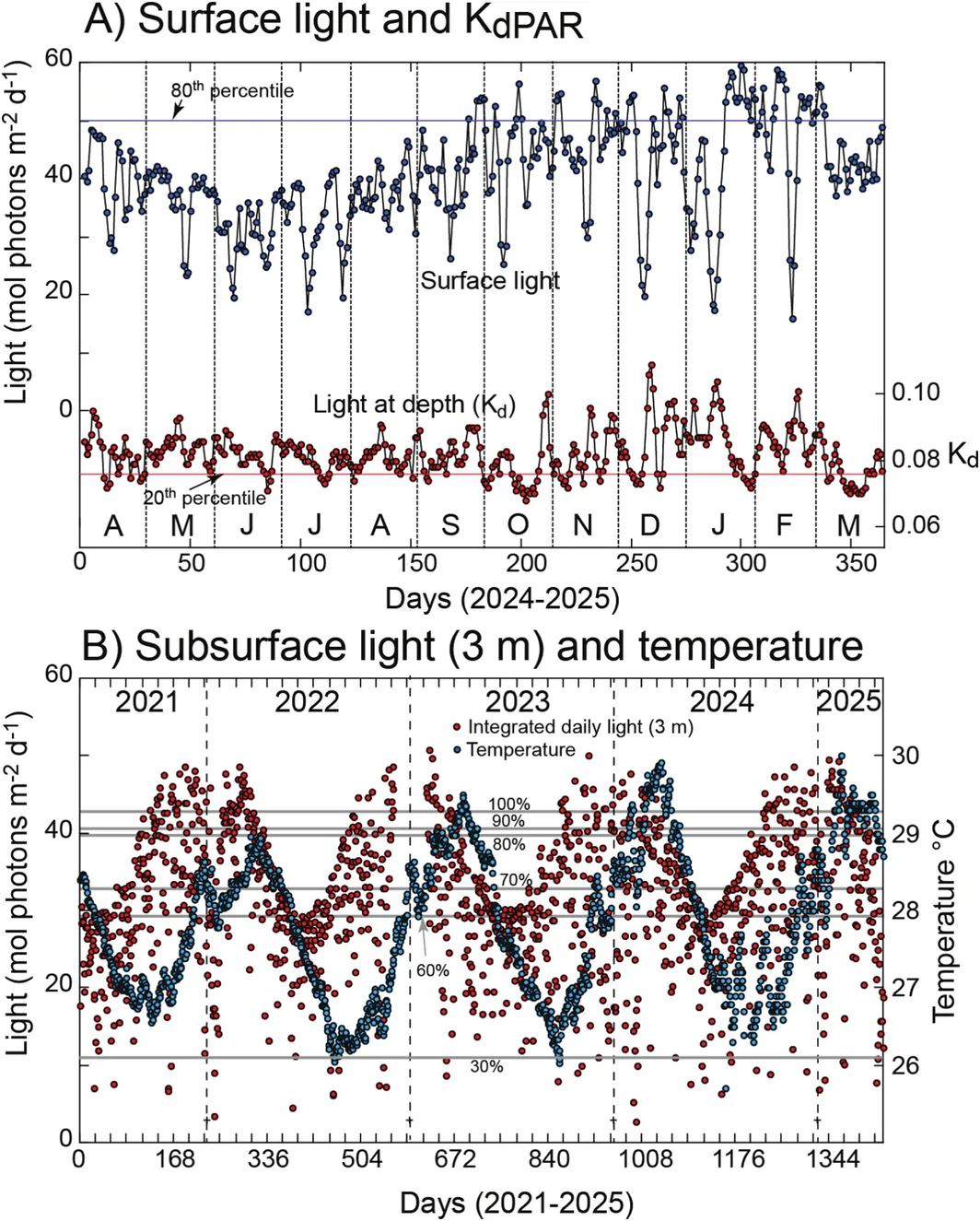
Light in Moorea on the surface and at 17‐m depth on the fore reef. (A) Surface light (integrated by day, left Y) and the molar extinction coefficient (*K*_d_, right Y) for PAR, both by day for 12 months from 1 April 2024 to 31 March 2025. The 80th and 20th percentile lines mark extreme values of high light levels at depth. (B) Light at the collection depth of the corals (3 m, integrated by day, left Y) and seawater temperature at 10‐m depth (right Y) on the fore reef, both by day for 5 years from 19 May 2021. Horizontal lines show the light treatment employed in the present study.

### 2.4 Incubation Conditions

The 12 tanks (each 150 L) were indoors and independently heated, chilled, illuminated, and supplied with sand‐filtered (100 μm pore size) seawater from PaoPao (Cook's) Bay. They were cooled by a common closed‐loop chilling system, heated by submerged heaters (200 W Jager), and illuminated by LED lights (AquaIllumination Hydra 64HD). Heaters and coolers were operated by digital controllers (Ranco, ±1°F for finer resolution cf. ±1°C), and the lights were programmed using the manufacturers' software (myAI, AquaIllumination). Six tanks were randomly assigned to one of two temperatures (28°C and 31°C), and one of six light regimes (30%, 60%, 70%, 80%, 90%, and 100% of maximum light output). The low temperature was ~1°C cooler than ambient seawater to alleviate early signs of bleaching when the corals were collected, and 31°C was just above the local bleaching threshold (29.8°C, Burgess et al. 2021).

The lights were operated on a 12:12 h photoperiod, with 4 h ramping at the start and end of each day so that the 100% setting created maximum and integrated light levels close to the mean values expected at the collection site (1667 ± 9 μmol photons m^−2^ s^−1^ and 31.2 ± 0.2 mol photons m^−2^ day^−1^ [*n* = 1368 days]). For each tank, temperature was recorded three times daily with a digital thermometer (Fisherbrand Traceable Digital Thermometer, ±0.02°C); light was recorded at noon with a 4‐π quantum sensor (Li Cor LI‐250A, at the height of the corals); and the rate of seawater addition was recorded with a timed fill of a measuring cylinder. The settings for temperature, light, and flow were adjusted to maintain target values. The corals were placed on the floor of each tank and rotated daily to alleviate position effects, and the tanks and plastic bases of the corals were cleaned daily with a brush to remove algae.

### 2.5 Dependent Variables

The biomass of freshly collected corals (18 corals in each trial) was determined by fixation in 5% formalin in seawater, decalcification in ~5% HCl in fresh water, rinsing of the tissue layer (released as a “thimble” when the skeleton was dissolved) in distilled water to remove salts, followed by drying to a constant weight at 60°C. Tissue dry weight was standardized to the 3‐dimensional area of the coral estimated from the area‐size relationship prepared with similar corals sampled in 2024 (Edmunds et al. 2025).

The response of corals exposed in the experiment to variation in temperature and light was evaluated by measuring the photophysiological performance of PSII of the algae and the holobiont condition. Photophysiological performance was used to evaluate the impacts of temperature and light in bleaching, and it was quantified using the minimum fluorescence yield (*F*_o_) and the maximum photochemical efficiency of open RCIIs (*F*_v_/*F*_m_) using a Diving‐PAM‐I fluorometer (Heinz Walz GmbH, Effeltrich, Germany). *F*_o_ was used as a coarse measure of the quantity of chlorophyll and the number of Symbiodiniaceae cells (Jones 2004; Jones et al. 2000) as they declined through bleaching, although *F*_o_ has limitations as a proxy for chlorophyll in corals (Ralph et al. 2016). *F*_v_/*F*_m_ was used to evaluate the functionality of PSII under different regimes of light and temperature.

The fluorometer was fitted with a 6‐mm‐diameter fiber optic probe that was held 5 mm above the upper surface on each colony. Preliminary trials were used to optimize the settings of the PAM fluorometer for corals freshly collected from the reef (gain = 12, measuring intensity = 11, saturation intensity = 12, damping = 2), and they were not changed throughout the experiment to ensure results were comparable across days and trials. Fluorescence measurements were completed after local darkness following > 2 h of dark adaptation and were made under dim red light. On the first day of each trial, 17 corals were randomly placed in each tank. That evening after dark‐adaptation, the fluorescence measurements of the remaining 12 corals in the acclimation tank were recorded to establish the baseline conditions of freshly collected corals. These 12 corals were randomly placed in the treatment tanks for a total of 18 corals tank^−1^. Thereafter, fluorescence was measured on all corals 24 h after placement in the treatments (*n* = 216), and then on 6 corals randomly selected from each tank every 2 days throughout the trial. The number of measurements in the analysis was reduced because not all corals delivered *F*_o_ values that were interpretable, particularly toward the end of each trial when the corals became pale. Following measurement of fluorescence, the corals were returned to their treatment tanks.

The condition of the corals was assessed on an ordinal scale of 0, 1, 2, or 3. Corals without tissue damage were assigned a score of 0. White corals with intact tissue were assigned a score of 1. White corals with tissue withdrawn into corallites and skeleton exposed between corallites, or with tissue sloughing off, were assigned a score of 2. Corals that were dead (i.e., no visible tissue) were assigned a score of 3. Corals were evaluated by careful inspection, initially while they were in the treatment tank when healthy, but as they deteriorated it was necessary to inspect them submerged in seawater (from their treatment tanks) using a dissecting microscope (40× magnification). Microscopic screening for the presence of tissue took 3–4 min for corals transitioning from a score of 2 to a score of 3 because tissue relicts remained on deteriorating corals for multiple days. For corals assigned a score of 2, inspection revealed translucent tissue between skeletal spines and vestigial tentacles surrounding the mouth of each polyp. Corals assigned a score of 1 would typically be recorded as bleached during in situ surveys of a coral reef, while corals assigned a score of 2 would probably be scored as dead without the aid of a microscope. Corals were scored on the health scale on every day of the experiment, and when dead (score = 3), they were removed from the treatment tanks.

### 2.6 Genetic Identification of the Corals

Swabbed corals were identified to the species level at the UC Gump South Pacific Research Station. First, the swab tips (in DNA/RNA stabilizing solution) were vortexed for 5 min. DNA was extracted from 10 μL of the DNA/RNA stabilizing solution by incubating it with 100 μL 10% Chelex 100 resin solution (Bio‐Rad Laboratories, Hercules, CA) for 1 h at 55°C, followed by 20 min at 95°C (Cohn et al. 2026a). After incubation, the supernatant containing genomic DNA was stored at −40°C until further processing.

For each sample, polymerase chain reaction (PCR) amplicons of the mitochondrial open reading frame (mtORF) and histone 3 (PocHistone) regions of the pocilloporid genome were generated using the respective primer sets: FatP6.1 and RORF for mtORF (Flot et al. 2008); and PocHistoneF and PocHistoneR for PocHistone (Johnston et al. 2018). PCRs were performed in 50 μL reactions containing 1× Taq PCR Master Mix (Qiagen, Germantown, MD), 0.2 μM each forward and reverse primer, 0.002% bovine serum albumin (BSA, ThermoFisher Scientific, Waltham, MA), and 1 μL genomic DNA. PCR cycling conditions for the mtORF amplification were: 94°C for 60 s, followed by 40 cycles of 94°C for 30 s, 53°C for 30 s, and 72°C for 75 s, and a final extension at 72°C for 5 min. PCR cycling conditions for PocHistone amplification were: 94°C for 60 s, followed by 40 cycles of 94°C for 30 s, 53°C for 30 s, and 72°C for 60 s, and a final extension at 72°C for 5 min (Cohn et al. 2026a).

Species identifications were then determined using a set of restriction fragment length polymorphism (RFLP) assays (Cohn et al. 2026a, 2026b), based on banding patterns from separate restriction enzyme digests run for each sample. In brief, the mitochondrial open reading frame (mtORF) amplicon was cut with the restriction enzyme *NlaIV* to distinguish *Pocillopora acuta* from all other species, and to group *P. tuahiniensis* + *P. verrucosa* , and *P*. cf. *effusa* + *P. meandrina* + *P. grandis* . For samples in the *P. tuahiniensis* + *P. verrucosa* group, the mtORF amplicon was cut with the restriction enzyme *BseYI/AciI* to separate *P. tuahiniensis* from *P. verrucosa* . For samples in the *P*. cf. *effusa* + *P. meandrina* + *P. grandis* group, the mtORF amplicon was cut with the restriction enzyme *EcoRV‐HF* to separate *P*. cf. *effusa* from *P. meandrina* + *P. grandis*. For samples in the *P. meandrina* + *P. grandis* group, the histone 3 (PocHistone) amplicon was cut with *XhoI* to separate *P. meandrina* from *P. grandis*.

For each sample, up to three separate restriction enzyme digests (NlaIV, EcoRV‐HF, and AciI‐BseYI digests; see Data S1 in Supporting Information, Cohn et al. 2026a) of the mtORF amplicons were conducted. For the NlaIV digest, 5 μL of the mtORF amplicons were added to 1 unit of *NlaIV* restriction enzyme (New England BioLabs, NEB, Ipswich, MA) in 0.5 μL 10× rCutSmart Buffer (NEB). For the EcoRV‐HFdigest, 5 μL of the mtORF amplicons were added to 10 units of *EcoRV‐HF* restriction enzyme (NEB) in 0.5 μL 10× rCutSmart Buffer. For the AciI‐BseYI digest, 5 μL of the mtORF amplicons were added to 5 units of *AciI* restriction enzyme (NEB) and 2.5 units of *BseYI* restriction enzyme (NEB) in 0.5 μL 10× NEBuffer r3.1 (NEB). Five μL of the PocHistone amplicons from each coral sample were also run in a XhoI digest (Data S1, Cohn et al. 2026a) with 10 units of *XhoI* restriction enzyme (NEB) in 0.5 μL 10× rCutSmart Buffer. In all restriction enzyme digests, amplicons were digested at 37°C for 1 h, followed by enzyme denaturation at 80°C for 20 min. Digested PCR amplicons and Quick‐Load Purple 50 bp DNA Ladder (NEB) were run on 2% agarose gels for 45–70 min at 100 V and visualized using SYBR Safe DNA Gel Stain (ThermoFisher Scientific, Waltham, MA) on a blue light transilluminator (Accuris Instruments, Edison, NJ, Cohn et al. 2026a).

### 2.7 Statistical Analysis

The genetically identified corals were divided into two groups: one consisting of the single most abundant species, and the other consisting of all taxa (pooled). The sample sizes of the individual taxa (other than the most common species) were too small to support statistical analysis of all species. In all models, trials and tanks nested within trials were included as random effects (Table 1).

**TABLE 1:** Summary of statistical replication employed in the mesocosm experiment.

Scale of inference	Scale at which the factor of interest is applied	Number of replicates at the appropriate scale
Population of *Pocillopora* corals around Moorea	Individual tanks (150 L) in mesocosm experiment	Two trials with 12 tanks (6 tanks randomly assigned across the light gradient at 28°C and 31°C; multiple corals per tank)

The scaling of log_10_(biomass) with log_10_(area) was estimated using a generalized linear mixed effects model (GLMM) that assessed the interactive and additive effects of trial and coral size. Physical conditions were compared among treatments and trials using GLMM's in which treatment was a fixed effect. Models were fit using restricted maximum likelihood (REML) and the assumptions of normality and equal variance were tested through graphical analysis of residuals.

The effect of temperature (categorical) and light (continuously distributed PAR values representing the mean per tank over the 14 days [Table 1]), on the photophysiological response of the corals (either *F*_o_ or *F*_v_/*F*_m_) was assessed using Gaussian GLMMs. Additionally, the change in *F*_v_/*F*_m_ over the 11 days (continuous) was assessed across temperature (categorical) treatments and light (continuous), using the PAR values measured in each tank on each day.

The effect of light (continuous) and temperature (categorical) on the probabilities of bleaching and partial and full mortality was estimated using binomial GLMMs. The probability of bleaching was estimated by coding corals in Condition Category 0 as “0” while those in health categories 1 and 2 were coded as “1” (Condition Category 3 corals that were dead were excluded from analysis). The probability of partial or full mortality was estimated by coding corals in condition categories of 0 and 1 as “0”, while those in Condition Categories 2 and 3 were coded as “1”. Too few corals died (Condition Category 3) to estimate the probability of survival in a GLMM. Separate models were run for Day 8, 11, and 14 when treatment effects started to be expressed during the 14‐day experiment. Day 11 is presented in the main text, while Days 8 and 14 are presented in the supplement.

Candidate models for each response variable included all additive and interactive combinations of predictor variables. For *F*_o_ and *F*_v_/*F*_m_, candidate models also included a second‐order polynomial term for light to assess potential curvi‐linear relationships. Model selection was conducted using Akaike's Information Criterion corrected for small sample size (AIC_c_) to determine how well additive and interactive parameters combinations fit the data. For each model we calculated ΔAIC_c_ values relative to the model with the lowest AIC_c_. When multiple models had ΔAIC_c_ ≤ 2, indicating similar support, we selected the most parsimonious model that retained both of the manipulated variables of temperature and light. From this simplest model, fixed effects were assessed using log‐likelihood ratio tests of models with and without the factor of interest. All analyses were completed in R 4.4.3 software using the glmmTMB package (version 1.1‐9, Brooks et al. 2025).

## 3 Results

### 3.1 Light and Temperature

From 2021 to 2025, maximum light at 17‐m depth ranged from 28 to 905 μmol photons m^−2^ s^−1^, with a mean (±SE) of 495 ± 4 μmol photons m^−2^ s^−1^ (*n* = 1370 days), and a mean *K*_dPAR_ (±SE) of 0.0862 ± 0.0003 (*n* = 1368). Using *K*_dPAR_ (Figure 1A) to calculate light at the collection depth of the corals, mean (±SE) integrated light at 3‐m depth was 50.9 ± 0.2 mol photons m^−2^ day^−1^. During the twelve‐month period ending on 31 March 2025, *K*_dPAR_ ranged from 0.0569 to 0.1176, and there were 75 days of clear water when *K*_dPAR_ was in the < 20th percentile of the frequency distribution of all values (i.e., low values reflecting clear water, Figure 1A). Integrated surface light over the same period ranged from 8.4 to 61.8 mol photons m^−2^ day^−1^, and 72 days were in the > 80th percentile of the frequency distribution of the data (i.e., unusually sunny and bright). Integrated surface light varied among days, sometimes declining 78% from 42.8 mol photons m^−2^ day^−1^ to 9.6 mol photons m^−2^ day^−1^ in a day.

Five years of integrated light at 3‐m depth and temperature (Figure 1B) revealed asynchronous seasonality. Maximum in situ light occurred close to the Austral summer equinox on ~21 December, but maximum temperature occurred 3–4 months later. In situ (3‐m depth) integrated light and temperature were positively associated (*r* = 0.140, df = 1355, *p* < 0.001), but only 1.9% of the variation in light was explained by temperature. Relative to the light treatments, integrated light at 3‐m depth frequently exceeded the values expected in the 100% light treatment (11% of days, *n* = 1368 days), as well as the 90% (17% days), 80% (21% days), and 70% (44% days) treatments.

### 3.2 Corals and Treatments

When the corals were collected in April 2025, seawater temperature in the back reef was 29.4°C (6th April, north shore). While some of the corals at the collection site were bleaching, small *Pocillopora* spp. were brown when freshly collected (Figure S1). The mean size (±SE) of these small corals was 21.6 ± 0.4 mm (range 7.5–40.5 mm; *n* = 233) in Trial 1, and 23.6 ± 0.4 mm (range 11.0–46.0 mm, *n* = 234) in Trial 2.

Species identifications were successful for 448 of 467 coral samples, including those used for the experiment and for the determination of the initial biomass (95.9%, Data S1 and S2). Ten samples could not be identified because PCR amplifications were unsuccessful; 9 samples could not be identified because one or more of the restriction enzyme digests were not performed (due to logistical constraints). Of the 467 samples, the assemblage was dominated by *P. meandrina* (70.4%, 329 samples), with *P. acuta* , *P*. cf. *effusa*, *P. grandis* , *P. tuahiniensis*, and *P. verrucosa* also represented (Data S1 and S2). The subset of corals used in the experiment (*n* = 432) shared a similar species composition to those used for the determination of the initial biomass (Table 2). Accordingly, we focused statistical analyses on *P. meandrina* , with results for all *Pocillopora* spp. (i.e., pooled among species) in Supporting Information.

**TABLE 2:** Number of colonies (and their percentage of corals used in the whole experiment, *n* = 467) of each *Pocillopora* species used in the experiment, the initial biomass determination, or combined.

Allocation	*P. acuta*	*P*. cf. *effusa*	*P. grandis*	*P. meandrina*	*P. tuahiniensis*	*P. verrucosa*	Unidentified	Total
Experiment	9 (2.1%)	6 (1.9%)	51 (11.8%)	307 (70.4%)	35 (7.9%)	7 (1.9%)	17 (4.1%)	432
Biomass	0 (0%)	3 (1.4%)	4 (11.8%)	22 (71.1%)	2 (8.1%)	2 (1.6%)	2 (3.9%)	35
All samples	9 (1.9%)	9 (1.9%)	55 (11.7%)	329 (70.4%)	37 (7.9%)	9 (1.9%)	19 (4.1%)	467

For biomass (dry weight), the most parsimonious model included additive effects of initial size (surface area) and trial (Table 3, Table S1). While the scaling of biomass with size did not differ between trials (𝝌^2^ = 2.170, df = 1, *p* = 0.140), their elevations did (𝝌^2^ = 9.585, df = 1, *p* = 0.002) (Figure 2). Mean biomass was 69% (26–128, 95% CI) higher in Trial 1 (14.2 ± 1.5 SE mg cm^−2^, *n* = 14) compared to Trial 2 (8.5 ± 0.9 SE mg cm^−2^, *n* = 8), for which corals were collected 16 days after those used in Trial 1. The biomass of all *Pocillopora* spp. displayed the same trend (Trial 1, 13.4 ± 1.3 mg cm^−2^, *n* = 17; Trial 2, 8.4 ± 0.9 mg cm^−2^, *n* = 18) (Figure S2).

**TABLE 3:** Statistical contrast of log_10_(biomass) of *Pocillopora meandrina* (Figure 2) between Trials with Log_10_ (area) as the covariate.

Model structure	*K*	ΔAIC_c_	Effect	*χ* ^2^	df	*p*
Area + Trial	4	0.00	Trial	9.585	1	0.002
			Area	32.362	1	< 0.001
Area * Trial	5	1.22	Area × Trial	2.176	1	0.140

*Note:* Refer to Figure S2 and Table S1 for results for all *Pocillopora* spp.

Abbreviations: ΔAIC_c_, change relative to the lowest value of AIC_c_ adjusted for small sample sizes; *K*, number of parameters.

**FIGURE 2:**
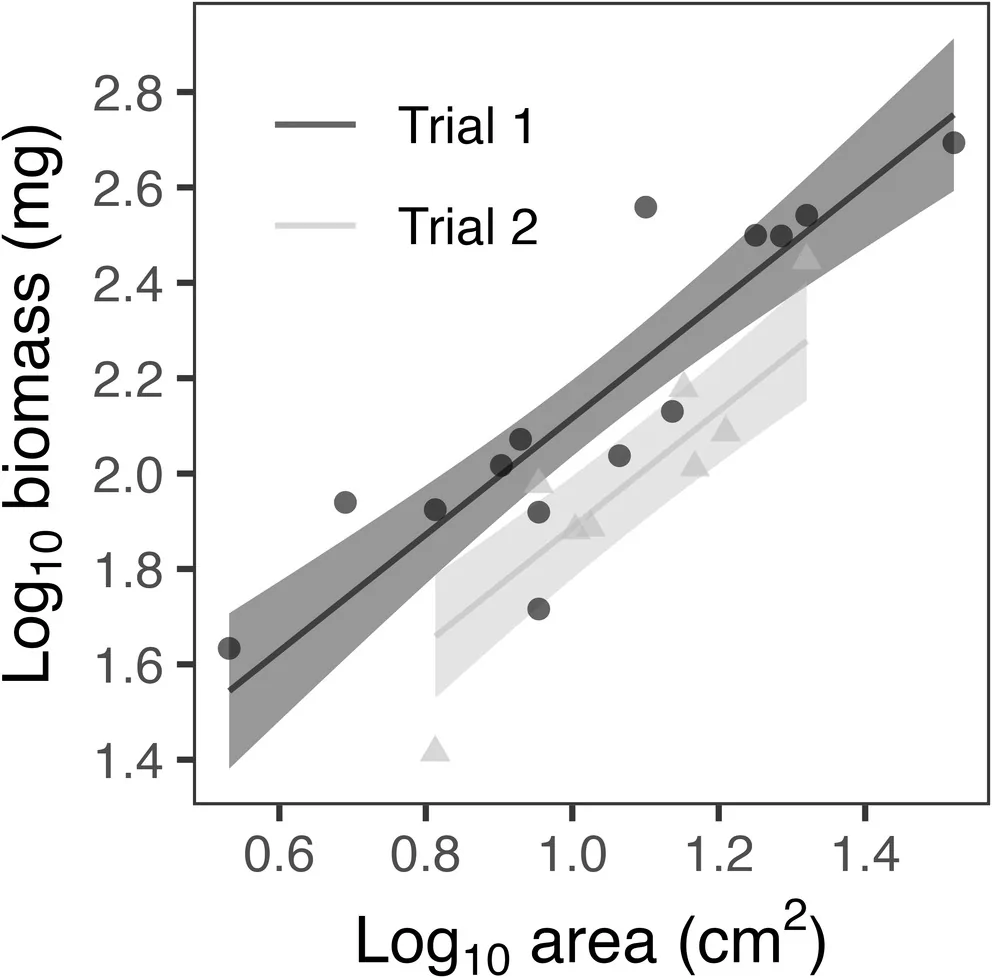
Log_10_ relationship between area (cm^2^) and biomass (mg) of *Pocillopora meandrina* in Trial 1 (*n* = 14) and Trial 2 (*n* = 8). Fitted lines and ribbons represent the mean and 95% confidence interval, respectively, estimated from a linear model with additive effects of trial and area. Refer to Figure S2 for results for all *Pocillopora* spp.

The physical conditions in the tanks were maintained precisely (Table 4). Conditions differed between treatments for temperature (*F* = 820.855, df = 1,1002, *p* < 0.001) that contrasted 28.20°C ± 0.04°C versus 30.98°C ± 0.07°C (mean ± SE, *n* = 12), and light (*F* = 17.322, df = 5342, *p* < 0.001) that varied from a low (i.e., the 30% treatment) of 470 ± 16 μmol photons m^−2^ s^−1^ to a high (i.e., 100% treatment) of 1381 ± 4 μmol photons m^−2^ s^−1^ (mean ± SE, *n* = 4) (Table 4). Fresh seawater flowed into each tank at 167 ± 5 mL min^−1^ (mean ± SE, *n* = 360).

**TABLE 4:** Tank conditions during Trial 1 and Trial 2.

Temperature	Light	Trial 1		Trial 2	
		Temp (°C)	Light μm m^−2^ s^−1^	Temp (°C)	Light μm m^−2^ s^−1^
Warm	30%	28.2 ± < 0.1	436 ± 9	28.1 ± < 0.1	448 ± 8
	60%	28.2 ± < 0.1	998 ± 15	28.2 ± 0.1	1024 ± 12
	70%	28.2 ± < 0.1	1228 ± 24	28.2 ± < 0.1	1085 ± 25
	80%	28.3 ± 0.1	1480 ± 29	28.0 ± < 0.1	1510 ± 26
	90%	28.5 ± 0.1	1436 ± 23	28.1 ± 0.1	1434 ± 17
	100%	28.2 ± 0.1	1385 ± 16	28.3 ± < 0.1	1371 ± 17
Hot	30%	30.8 ± < 0.1	493 ± 12	30.8 ± < 0.1	502 ± 7
	60%	31.3 ± < 0.1	1004 ± 14	30.8 ± 0.1	1012 ± 14
	70%	31.2 ± 0.1	1124 ± 17	30.9 ± < 0.1	1123 ± 18
	80%	31.4 ± 0.1	1127 ± 13	31.3 ± 0.1	1117 ± 18
	90%	30.6 ± < 0.1	1390 ± 21	30.7 ± 0.1	1379 ± 24
	100%	31.1 ± 0.2	1559 ± 40	30.9 ± 0.2	1621 ± 17

*Note:* For temperature, *n* = 41–42 for both trials; for light, *n* = 15 (Trial 1) and 14 (Trial 2).

### 3.3 Photophysiological and Condition Rank Response

For freshly collected *P. meandrina* , mean (±SE) *F*_v_/*F*_m_ was 0.653 ± 0.009 (*n* = 9) for Trial 1 and 0.668 ± 0.003 for Trial 2 (*n* = 7), with a mean *F*_o_ of 557 ± 21 and 896 ± 92, respectively.

For *F*_o_ in *P. meandrina* on Day 11, the most parsimonious model included additive effects temperature and light (Table 5, Table S2). There were no interactive effects of temperature and light on *F*_o_ ($\chi^{2}$ = 0.064, df = 1, *p* = 0.801), and there were no additive effects of temperature ($\chi^{2}$ = 0.605, df = 1, *p* = 0.437). *F*_o_ declined linearly with light at both temperatures ($\chi^{2}$ = 9.689, df = 1, *p* = 0.002) and there was no evidence of a curvilinear relationship with light ($\chi^{2}$ = 1.854, df = 1, *p* = 0.173) (Table 5, Figure 3A, Figure S4, Table S2).

**TABLE 5:** Statistical analysis of photophysiology (*F*_o_ and *F*_v_/*F*_m_ [Figure 3, Figure S3]) and health ranks (Figure 5) for *Pocillopora meandrina* exposed to two temperatures and six light levels (Table 2) in two 14‐day trials with responses evaluated every 2 days from Days 1 to 14.

DV	Model structure	Effect	χ^2^	df	*p*
*F*_o_ (Day 11)	Light + temperature	Temperature	0.605	1	0.44
		**Light**	**9.689**	**1**	**0.002**
*F*_v_/*F*_m_ (Day 11)	Light * temperature	**Light × temp**	**10.925**	**1**	**0.001**
*F*_v_/*F*_m_ over time (across Days 1 through 11)	Day * light * temperature	**Day × light × temp**	**15.886**	**1**	**< 0.001**
Health rank: bleaching	Light + temperature	**Light**	**22.067**	**2**	**< 0.001**
(0 vs. 1 + 2)		Temperature	0.563	1	0.453
Health rank: partial or full mortality	Light + temp	**Temperature**	**14.681**	**1**	**< 0.001**
(0 + 1 vs. 2 + 3)		**Light**	**14.237**	**1**	**< 0.001**

*Note:* Results for *F*_o_ and *F*_v_/*F*_m_ presented for Day 11, across Days 1 to 11 for *F*_v_/*F*_m_, and for Day 14 for health ranks; refer to Table S3 for results from other days. *F*_o_ and *F*_v_/*F*_m_ analyzed with LME and health rank with GLMM, in which temperature and light are fixed effects, and trials and tanks as random effects; analyses for bleaching contrasts health rank 0 versus (1 + 2), partial or full mortality contrasts health rank 0 + 1 versus 2 + 3. Results for other analyzed models in Table S2. DV, dependent variable; *χ* ^2^, chi‐square statistic; df, degrees of freedom; *p*, probability; significant effects in bold.

**FIGURE 3:**
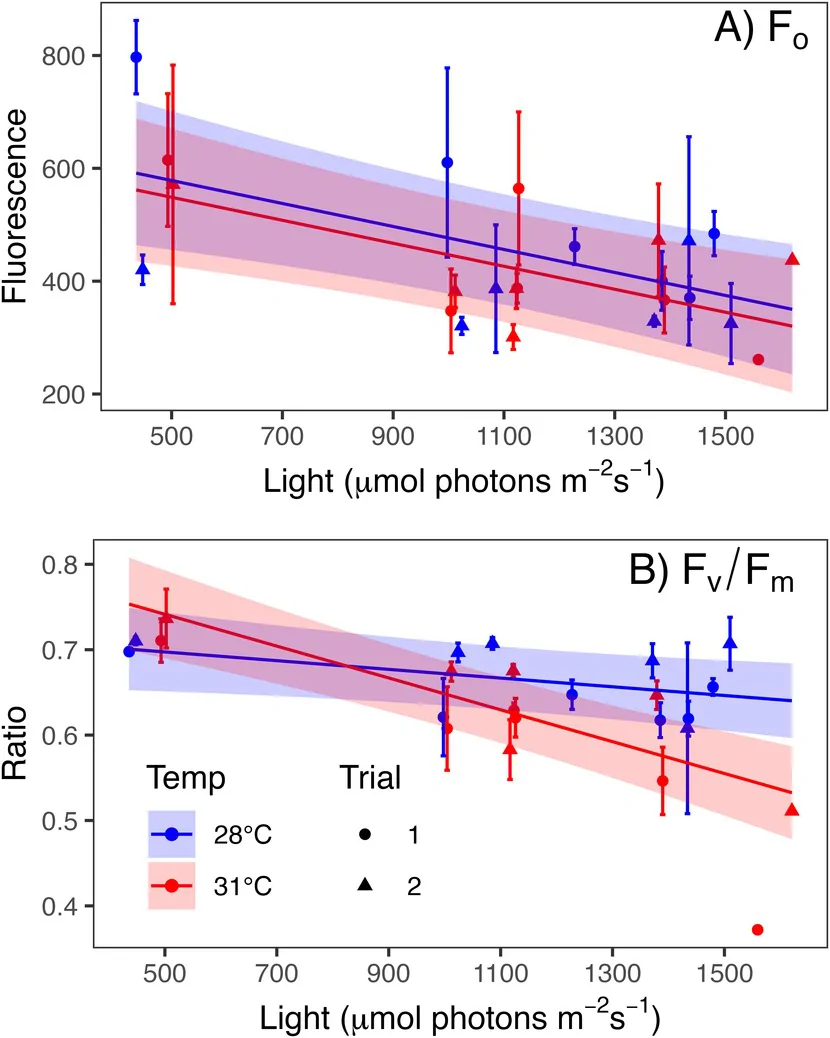
Photophysiological performance of *Pocillopora meandrina* after 11 days in two temperatures across a light gradient in two trials. Response variables were: (A) minimum fluorescence yield (*F*_o_), and (B) maximum photochemical efficiency of open RCIIs (*F*_v_/*F*_m_). The mean (±SE) maximum irradiance at 3‐m depth (the collection depth for the corals) was 1667 ± 9 μmol photons m^−2^ s^−1^ (*n* = 1368 days, 19 May 2021 to 29 April 2025), as calculated from *K*_dPAR_ at 17‐m depth on the fore reef of Moorea. Points show the mean ± SE for each tank (*n* = 1–6 by tank). Lines and shading show the fitted values and 95% CI, respectively, from GLMM's with trial and tanks within trials as random effects. Refer to Figure S3 for results for all *Pocillopora* spp.

For *F*_v_/*F*_m_, the most parsimonious model included the interaction between temperature and light (Table 5, Table S2), and there were interactive effects of temperature and light on *F*_v_/*F*_m_ ($\chi^{2}$ = 10.925, df = 1, *p* = 0.001). At 28°C, *F*_v_/*F*_m_ declined by 0.0051 (0.0006–0.0096, 95% CI) for every 100 μmol photons m^−2^ s^−1^ increase in light. At 31°C, the rate of decline in *F*_v_/*F*_m_ with light was 0.0136 (0.0057–0.0214, 95% CI) units per 100 μmol photons m^−2^ s^−1^ faster than at 28°C (Table 5, Figure 3B, Table S2). The results for all *Pocillopora* spp. are reported in Figure S4.

With respect to the effects of light and temperature on *F*_v_/*F*_m_ over 11 days (Figure 4A,B), there were interactive effects of day, light, and temperature ($\chi^{2}$ = 15.886, df = 1, *p* < 0.001, Table 5). At 31°C, *F*_v_/*F*_m_ declined most rapidly at the highest light level (1707 μmol photons m^−2^ s^−1^), which commonly was reached at 3‐m depth (Figure 4C). Results for all *Pocillopora* spp. are reported in Figure S4, Table S3.

**FIGURE 4:**
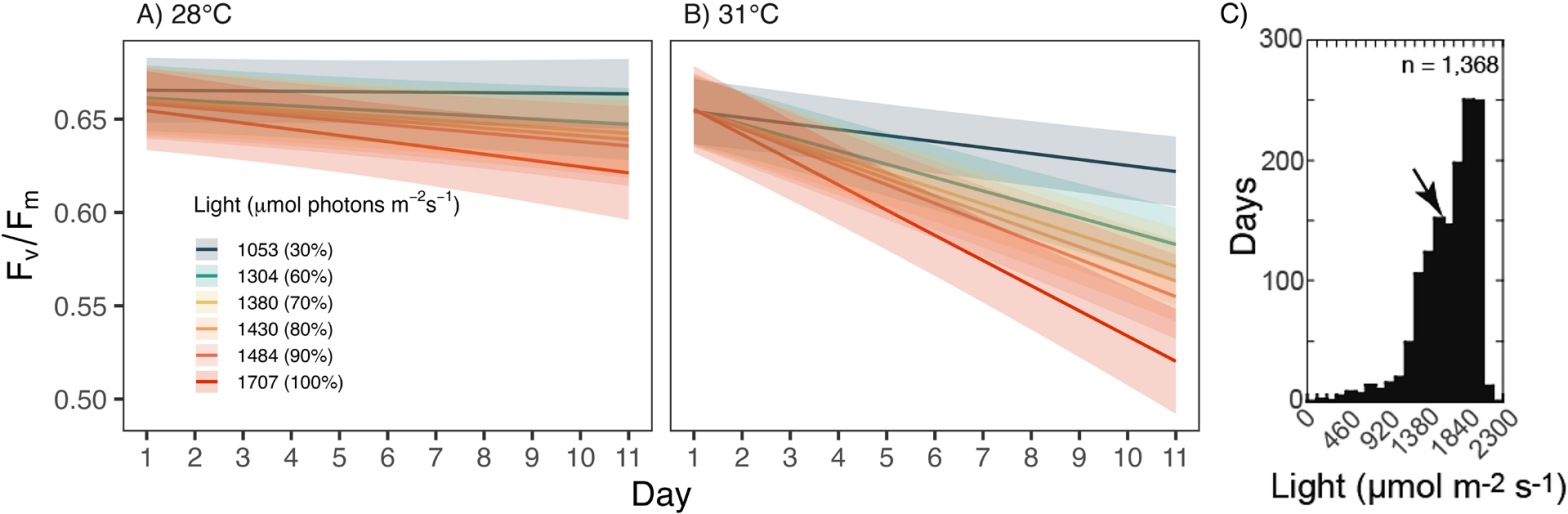
Maximum photochemical efficiency of open RCIIs (*F*_v_/*F*_m_) of *Pocillopora meandrina* at 28°C (A) and 31°C (B) over time at different light levels, together with the frequency distribution of daily integrated light at the collection depth from 19 May 2021 to 29 April 2025 (C). Fitted lines and ribbons in A and B represent the mean and 95% CI estimated from a linear effects model with fixed effects of day, temperature and light (as a continuous variable), with trial and tanks within trials as random effects. Refer to Figure S4 for results for all *Pocillopora* spp.

For the probability of bleaching (health rank 1 + 2) by Day 14, the most parsimonious model included additive effects temperature and light (Table 5, Table S2). By Day 14, the probability of bleaching in *P. meandrina* increased with light ($\chi^{2}$ = 22.067, df = 1, *p* < 0.001), but there were no interactive ($\chi^{2}$ = 0.051, df = 1, *p* = 0.821) or additive effects of temperature ($\chi^{2}$ = 0.563, df = 1, *p* = 0.453) (Figure 5A, Table 5). The odds of bleaching rose by 86.7% (48.24–135.29%, 95% CI) for every 100 μmol photons m^−2^ s^−1^ increase in light.

**FIGURE 5:**
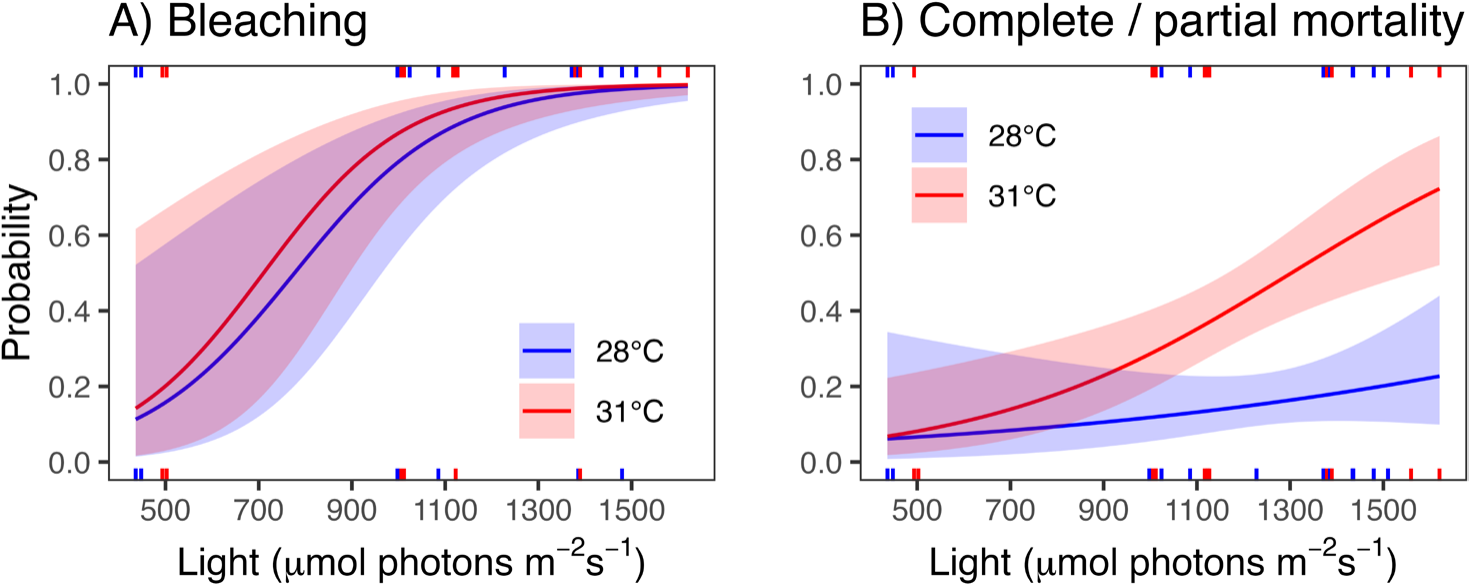
Effect of light on: (A) the probability of bleaching, and (B) partial or full mortality in small *Pocillopora meandrina* by Day 14 under 28°C or 31°C (8–16 corals per tank). Rugs on upper and lower side of plot frames show records by coral. Lines and shading show the fitted values and 95% CI, respectively, from a binomial GLMM with trial and tanks within trials as random effects. Refer to Supporting Information, for results for Days 8 and 11 (Figure S5), and Figures S6 and S7 for results for all *Pocillopora* spp.

For the probability of partial or full mortality (health rank 2 + 3) by Day 14, the most parsimonious model also included additive effects of temperature and light (Table 5, Table S2). The probability of partial or full mortality increased with light ($\chi^{2}$ = 14.237, df = 1, *p* < 0.001) and temperature ($\chi^{2}$ = 14.681, df = 1, *p* < 0.001) (Figure 5B, Table 5). The odds of partial or full mortality by Day 14 rose by 25.66% (7.61–46.75, 95% CI) for every 100 μmol photons m^−2^ s^−1^ increase in light, and were 4.54 (2.33–8.86, 95% CI) times greater at 31°C compared to 28°C (at all light levels). There were no interactive effects of temperature and light on the probability of partial or full mortality by Day 14 ($\chi^{2}$ = 2.279, df = 1, *p* = 0.131). Results for *P. meandrina* for Days 8 and 11 are shown in Figure S5, and results for all *Pocillopora* spp. are reported in Figures S6 and S7.

Throughout both trials, 13 corals (3%) died (pooled among species) and all were in the 31°C treatment, with 6 at 1621 μmol photons m^−2^ s^−1^, 4 at 1559 μmol photons m^−2^ s^−1^, and 1 at each of 493, 1004, and 1127 μmol photons m^−2^ s^−1^. Six of the dead corals were *P. meandrina* , 6 were *P. tuahiniensis*, 1 was *P. acuta* , and 1 was an unidentified species. Too few corals died (i.e., health rank 3) to support statistical analysis of whole‐colony survivorship by GLMM.

## 4 Discussion

Reef corals that partner with symbiodiniacean algae rely on light for phototrophy (Muscatine 1990; Roth 2014), nutrient uptake (Muscatine and D'Elia 1978), rapid calcification (Allemand et al. 2011), directional growth (Kaniewska et al. 2009), larval behavior (Strader et al. 2015), and reproductive timing (Boch et al. 2011). They are strongly affected by the quantity and quality of light, for example, acclimatizing in deep water to utilize blue light (Kahng et al. 2012), bleaching in response to prolonged darkness (Franzisket 1970), and bleaching more severely at high temperatures when exposed to bright light (Fitt and Warner 1995; Porter et al. 1989) and UV radiation (Lesser et al. 1990). Many of the mechanisms underlying the role of light in bleaching are well known (Helgoe et al. 2024; Mumby et al. 2001; Rowan et al. 1997), yet recent studies of coral bleaching have paid limited attention to these effects (e.g., Shlesinger and van Woesik 2023). This omission may reflect the scant number of measurements of underwater light that can be used for context (Yentsch et al. 2002), and our incomplete understanding of the mathematical functions describing the light‐bleaching and light‐mortality relationships (Rosic et al. 2020; Skirving et al. 2017). We addressed these limitations by describing light on a coral reef over five years, providing experimental support for the hypothesis that light affects the response of small *Pocillopora meandrina* to temperature, and by codifying and quantifying their curvilinear relationships between light and bleaching. Our results begin to identify the conditions under which *Pocillopora* spp. settlers have an increased chance of surviving to contribute to coral population growth at elevated temperature (i.e., when light levels are low).

Our study describes how small *Pocillopora meandrina* respond to elevated temperatures over a range of light levels relevant to back reef habitats. Our broader objective was to quantify the curvilinear relationship between physiological stress and light in early life stages of ecologically important reef building corals (i.e., *Pocillopora* spp.) in the Indo‐Pacific (Pinzón et al. 2013). We surmise that this relationship is an important aspect of the conditions determining whether coral settlers grow into small colonies and become adult corals, the early stages of which can create a bottleneck that mediates population growth (Doropoulos et al. 2016; Edmunds 2021; Speare et al. 2025). Previously, we have shown how a “physiological crisis” can develop when small corals respond to the high selective pressure for rapid growth by consuming food reserves to meet the costs of growth at the expense of increasing the risks of mortality through resource limitation (Edmunds et al. 2025). Our present conclusions enhance understanding of the coral recruitment bottleneck by describing how light and temperature mediates the demise (or survival) of small *P. meandrina* as well as *Pocillopora* spp.

The success of symbiotic corals is determined by the quality and quantity of light (Dubinsky and Falkowski 2010; Kahng et al. 2012; Roth 2014) and, therefore, there is value in measuring light underwater on a coral reef. Specialized equipment is required for this task (Long et al. 2012), and when it is deployed for more than a few days, the sensor must routinely be cleaned of fouling organisms (Delauney et al. 2010). Interpretation of the data obtained from such sensors benefits from concurrent records of surface light that allow the causes of variation in underwater light to be resolved between surface and subsurface effects. These effects can be large and unpredictable because, for example, the consequences of clouds and rain for underwater light are reduced when they coincide with clear seawater and the reef is offshore and unaffected by runoff. Together with long‐period variation in light caused by solar declination (Brock 1981), and global dimming (Wild 2009), there are multiple mechanisms by which underwater light on a coral reef can vary over days‐years and at high frequency when clouds and wave focusing are considered (Wing and Patterson 1993). Because our light treatments are ecologically relevant (Figure 1B), it is reasonable to expect the in situ physiological responses of small *P. meandrina* to 31°C versus 28°C to vary by at least the extent we have described whenever light quantities are like those of our treatments. This can occur throughout the year (Figure 1), as well as in microhabitats with light reduced by the cardinal orientation of coral colonies (Brown et al. 2002) or topography and topographic complexity (Liesegang et al. 2025). Together, these trends indicate that the negative implications of high temperature for corals can be reduced through natural occurrences of reduced light.

The corals used in the present experiment did not show signs of bleaching and were a “normal” brown color when collected (Figure S1). Nonetheless, corals collected for the second trial had lost 40% of their biomass relative to corals collected 16 days before (Figure 2), indicating the outcome of ongoing thermal stress (Fitt et al. 2000; Szmant and Gassman 1990). When placed in the treatments, all of the corals bleached, particularly at high light levels, and by the 11th day *F*_o_ was inversely related to the quantity of light, declining ~33% as light increased from ~500 μmol photons m^−2^ s^−1^ to ~1300 μmol photons m^−2^ s^−1^; it was not, however, affected by temperature (Figure 3A). When measured with constant settings on the Diving PAM fluorometer, *F*_o_ is loosely related to the amount of chlorophyll (and number of algal cells) in coral tissue (Jones 2004; Jones et al. 2000; Ralph et al. 2016). Therefore, the declines in *F*_o_ with increasing light suggest that paling of the corals was associated with a loss of chlorophyll and algal cells. Although these corals would have been scored as “bleached” if encountered on the reef (PJ Edmunds pers. observation), *F*_v_/*F*_m_ showed limited evidence of damage because the best‐fit values against light remained ≳ 0.600 (Figure 3B). Declines in *F*_v_/*F*_m_ can reveal impaired PSII functionality (Warner et al. 1999), with values persistently < 0.500 associated with bleaching (Jones et al. 2000; Warner et al. 1999), which depresses the capacity for photoautotrophy (Porter et al. 1989). Although light accelerated the depression of *F*_v_/*F*_m_ at high temperature in the present study, the relatively high values of *F*_v_/*F*_m_ on Day 11 suggest that the relict algal populations remained functional at most light levels (Jones et al. 2000; Warner et al. 1999). Nonetheless, the negative response to 31°C was accentuated under the brightest light, under which *F*_v_/*F*_m_ rapidly declining to < 0.500 indicating physiologically damaged (Figure 4B). This effect probably contributed to the light‐dependent rise in partial and complete mortality at 31°C (Fitt et al. 2009) (Figure 5B).

This study was completed with an assemblage of *Pocillopora* spp. from the eastern shore of Moorea, and genetic analysis showed that it was dominated by *P. meandrina* (70% of corals), which is consistent with previous genetic analysis of *Pocillopora* spp. collected from around Moorea in December 2021 and 2022 (Burgess et al. 2024). The present analyses therefore focus on the most robust outcome for *P. meandrina* , with complementary analyses for *Pocillopora* spp. in the Supporting Information. While the complementary analyses reinforce the outcomes for *P. meandrina*, we recognize the limitations of small sample sizes for the less abundant *Pocillopora* spp. in our study. Previous work has revealed phenotypic differences among *Pocillopora* spp. in their bleaching susceptibility (Burgess et al. 2021), spawning times (Harnay et al. 2025), and responses to resource limitation (Edmunds et al. 2025), and it is reasonable to expect these kinds of differences to scale up to affect demographic rates. It is likely that future experiments with large enough sample sizes for all cryptic species will reveal phenotypic differences among cryptic species of *Pocillopora*, notably with respect to the bleaching response when challenged by high temperature and elevated light.

Demonstrating that light interacts with high temperature to enhance coral bleaching is confirmatory with respect to the categorical effects of light (i.e., high vs. low) (Fitt et al. 2001; Helgoe et al. 2024; Lesser 2011), but quantifying the effect of light as a continuous gradient on bleaching and mortality is an important advance for the field. Such relationships can be used to predict the effects of high temperature in causing bleaching when quantitative underwater light regimes are available. Our study also advances the discipline because it was developed in an ecologically relevant context provided by in situ measurements of light, and it focused on survival in an early coral life stage, which often has the greatest influence on population growth (Doropoulos et al. 2016). Further, by measuring mortality as a response variable, it is possible to begin to couple the inferences from our experiments to the consequences for fitness (Crone 2001). Given the wide suite of conditions causing underwater light to vary, accounting for in situ light regimes on reefs will benefit efforts to understand when corals will bleach (Helgoe et al. 2024; Skirving et al. 2017), cases where bleaching is less severe than expected (Lachs et al. 2023), and the constraints placed upon the survival of newly settled and small corals (Doropoulos et al. 2016; Hughes and Jackson 1985; Speare et al. 2025).

## Author Contributions

**P. J. Edmunds:** conceptualization (equal), data curation (equal), formal analysis (equal), funding acquisition (lead), investigation (lead), methodology (lead), project administration (lead), supervision (lead), validation (lead), visualization (equal), writing – original draft (equal), writing – review and editing (equal). **K. C. Scafidi:** data curation (equal), investigation (equal), methodology (equal), writing – review and editing (equal). **F. Cohn:** methodology (supporting), writing – review and editing (supporting). **K. Layagala:** methodology (supporting), writing – review and editing (supporting). **J. A. Sims:** methodology (supporting), writing – review and editing (supporting). **A. M. S. Correa:** funding acquisition (supporting), methodology (supporting), project administration (supporting), resources (supporting), supervision (supporting), writing – review and editing (supporting). **S. C. Burgess:** conceptualization (equal), formal analysis (lead), methodology (supporting), visualization (lead), writing – original draft (equal), writing – review and editing (equal).

## Funding

This project was funded through the Moorea Coral Reef LTER of the US National Science Foundation (OCE 22‐24254 to P.J.E., and OCE 18‐29867 to S.C.B) and a US NSF Career Award (OCE 2533138 to A.M.S.C.).

## Conflicts of Interest

The authors declare no conflicts of interest.

## Supporting information


**Figure S1:** Photographs of representative colonies of *Pocillopora* spp. collected from the back reef of Moorea to support Trial 1 (A) and Trial 2 (B).
**Figure S2:** Log_10_ relationship between area (cm^2^) and biomass (mg) for all *Pocillopora* spp. in Trial 1 ( *P. meandrina* , *n* = 12; *P. grandis* , *n* = 1; *P. tuahiniensis*, *n* = 2) and Trial 2 ( *P. meandrina* , *n* = 8; *P*. cf. *effusa*, *n* = 3; *P. grandis* , *n* = 3; *P. verrucosa* , *n* = 2, no ID, *n* = 2). Fitted lines and ribbons represent the mean and 95% confidence interval, respectively, estimated from a linear model with interactive effects of trial and area. Refer to Figure 2 for results from *P. meandrina* alone. Although based on a small sample size, biomass in the “other” *Pocillopora* followed the same trend as for *P. meandrina* , with mean values 16% higher in Trial 1 (9.6 ± 1.8 mg cm^−2^, *n* = 3) versus Trial 2 (8.3 ± 1.4 mg cm^−2^, *n* = 10).
**Figure S3:** Photophysiological performance of all *Pocillopora* spp. incubated for 11 days in combinations of two temperatures and six light levels in two trials. A randomly selected subset of corals was analyzed for photophysiology at each time point. For measurements on Day 11: Trial 1 included 40 *P. meandrina* , 1 *P. acuta* , 5 *P. grandis* , 4 *P. tuahiniensis*, 1 *P. verrucosa* , and 4 no ID; Trial 2 includes 37 *P. meandrina* , 2 *P. acuta* , *4 P*. cf. *effusa*, 30 *P. grandis* , 22 *P. tuahiniensis*, 6 *P. verrucosa* , and 7 no ID. Two response variables were measured: (A) minimum fluorescence yield (*F*_o_), and (B) maximum photochemical efficiency of open RCIIs (*F*_v_/*F*_m_). The mean (±SE) maximum irradiance at 3‐m depth (the collection depth for the corals) was 1667 ± 9 μmol photons m^−2^ s^−1^ (*n* = 1368 days, 19 May 2021 to 29 April 2025), as calculated from *K*_dPAR_ at 17 m depth on the fore reef of Moorea. Points show the mean ± SE for each tank (*n* = 2–6 by tank). Lines and shading show the fitted values and 95% CI from a linear mixed effects model with fixed effects of temperature and light, and random effects of trial and tank. On Day 11, there were no interactive effects of temperature and light on *F*_o_ ($\chi^{2}$ = 1.018, df = 2, *p* = 0.601), but *F*_o_ declined curvi‐linearly with light ($\chi^{2}$ = 18.779, df = 2, *p* < 0.001) and was similar in both temperatures ($\chi^{2}$= 0.248, df = 1, *p* = 0.618). *F*_v_/*F*_m_ was significantly affected by the interaction of temperature with light ($\chi^{2}$ = 4.648, df = 1, *p* = 0.031).
**Figure S4:** Maximum photochemical efficiency of open RCIIs (*F*_v_/*F*_m_) of *Pocillopora* spp. at 28°C (A) and 31°C (B), together with the frequency distribution of daily integrated light at the collection depth from 19 May 2021 to 29 April 2025 (C). Fitted lines and ribbons in A and B represent the mean and 95% CI estimated from a linear effects model with fixed effects of day, temperature and light (as a continuous variable), with tank and trial as random effects. Refer to Figure 4 for results for *Pocillopora meandrina* . *F*_v_/*F*_m_ was affected by the interaction of day, temperature, and light ($\chi^{2}$ = 28.166, df = 10, *p* = 0.002, Table S3). At 31°C, *F*_v_/*F*_m_ declined most rapidly at the highest light level, which commonly was reached at 3‐m depth.
**Figure S5:** The effect of light on the probability of bleaching (rank 1 + 2), and partial or full mortality (rank 2 + 3) in small *Pocillopora meandrina* by Days 8 (A, B) and 11 (C, D) at 28°C or 31°C and six light levels (*n* = 8–16 per tank) (Day 14 displayed in Figure 5). Lines and shading show the fitted values and 95% CI, respectively, from a binomial linear mixed effects plot with fixed affects of temperature and light and tank as a random effect.
**Figure S6:** The effect of light on: (a) the probability of bleaching (rank 1 + 2), and (b) partial or full mortality (rank 2 + 3) in all small *Pocillopora* spp. by Day 14 under 28°C or 31°C and six light levels (*n* = 12–18 per tank). Rugs on upper and lower side of plot frames show records by coral. Lines and shading show the fitted values and 95% CI, respectively, from a binomial linear mixed effects model with fixed effects of temperature and light and tank as a random effect. The probability of bleaching (condition rank 1 + 2) by Day 14 increased with light (*χ* ^2^ = 27.364, df = 1, *p* < 0.001), but was unaffected by temperature ($\chi^{2}$ = 1.785, df = 1, *p* = 0.182). The odds of bleaching rose by 81.56% (53.03–115.41, 95% CI) for every 100 μmol photons m^−2^ s^−1^ increase in light. There was no interactive effect of temperature and light on the probability of bleaching after 14 days (*χ* ^2^ = 0.159, df = 1, *p* = 0.690). The probability of partial or full mortality (condition rank 2 + 3) by Day 14 increased with light ($\chi^{2}$ = 16.429, df = 1, *p* < 0.001) and decreased with temperature ($\chi^{2}$ = 14.739, df = 1, *p* < 0.001). The odds of partial or full mortality by Day 14 rose by 27.22% (13.17–43.01, 95% CI) for every 100 μmol photons m^−2^ s^−1^ increase in light, and were 341% (2.31–8.44, 95% CI) higher at 31°C compared to 28°C (at all light levels). There were no interactive effects of temperature and light on the probability of partial or full mortality by Day 14 ($\chi^{2}$ = 2.179, df = 1, *p* = 0.140).
**Figure S7:** The effect of light on the probability of bleaching (rank 1 + 2), and partial or full mortality (rank 2 + 3) in all small *Pocillopora* spp. by Days 8 (A and B) and 11 (C and D) at 28°C or 31°C and six light levels (*n* = 12–18 per tank) (Day 14 displayed in Figure 5). Lines and shading show the fitted values and 95% CI, respectively, from a binomial linear mixed effects model with fixed effects of temperature and light and tank as a random effect.
**Table S1:** Statistical contrast of log_10_(biomass) of all *Pocillopora* spp. (Figure S2) between Trials with Log_10_(area) as the covariate. Refer to Figure 2 and Table 3 for the results for *P. meandrina* .
**Table S2:** Results of model selection for analysis of *Pocillopora meandrina* using LME or GLMM to test for variation in *F*_o_, *F*_v_/*F*_m_, and condition rank as a function of temperature and light with trial and tanks as random effects. Results for *F*_o_ and *F*_v_/*F*_m_ presented for Day 11, Day 1 through 14 for *F*_v_/*F*_m_, and for Day 14 for condition ranks; refer to Table S3 for results from other days. Analyses for bleaching contrasts condition rank 0 versus (1 + 2), severe bleaching contrasts condition rank 1 versus 2, and mortality contrasts condition rank 0 + 1 versus 2 + 3. DV, dependent variable; ΔAIC_c_, change relative to the lowest value of AIC_c_; *K*, number of parameters. Best supported models (with lowest AICc or those differing by ≲ 2.0) in bold, with significance of the terms within those models reported in Table 4.
**Table S3:** Results of model selection for analysis of all *Pocillopora* spp. using LME or GLMM to test for variation in *F*_o_, *F*_v_/*F*_m_, and condition rank as a function of temperature and light with tank as a random effect. Results for *F*_o_ and *F*_v_/*F*_m_ presented for Day 11, all days for *F*_v_/*F*_m_ at the three highest light levels, and for Day 14 for condition ranks. Analyses for bleaching contrasts condition rank 0 versus (1 + 2) and mortality contrasts condition rank 0 + 1 versus 2 + 3. DV, dependent variable; ΔAIC_c_, change relative to the lowest value of AIC_c_; *K*, number of parameters, and best supported models (with lowest AIC_c_ or those differing by ≲ 2.0) in bold.

**Data S1:** ece374505‐sup‐0002‐SupplementaryMaterial2.pdf.

**Data S2:** ece374505‐sup‐0003‐SupplementaryMaterial3.pdf.

## Data Availability

All data reported in this paper are available from http://bco‐dmo.org/10.6073/pasta/b77f4251e2877070d74e53fe29279ac0 (Moorea Coral Reef LTER and Edmunds 2026) and as Supporting Information (gel pictures).
