# Supplementary material for: The Role of Light in Mediating Physiological Stress in Small Corals Exposed to Elevated Temperature
Source: Ecol Evol. 2026 Nov 12;16(11):e74505. doi: 10.1002/ece3.74505 (PMC13756585; doi:10.1002/ece3.74505)
Supplement: Supplementary file 1 [file ECE3-16-e74505-s001.pdf]

Supplementary Material 3

The role of light in mediating physiological stress in small corals exposed to elevated temperature

Sample	Sample Type	NlaIV Gel	NlaIV Result	EcoRV Gel	EcoRV Result	Acil-BseYI Gel	Acil-BseYI Result	XhoI Gel	XhoI Result	Species ID
1	Experiment	Gel 1	Run EcoRV	Gel 12	Run XhoI			Gel 26	*P. grandis*	*P. grandis*
2	Experiment	Gel 1	Run EcoRV	Gel 12	Run XhoI			Gel 26	*P. meandrina*	*P. meandrina*
3	Experiment	Gel 1	Run EcoRV	Gel 12	Run XhoI			Gel 26	*P. meandrina*	*P. meandrina*
4	Experiment	Gel 1	Run EcoRV	Gel 12	Run XhoI			Gel 26	*P. grandis*	*P. grandis*
5	Experiment	Gel 1	Run EcoRV	Gel 12	Run XhoI			Gel 26	*P. meandrina*	*P. meandrina*
6	Experiment	Gel 1	Run EcoRV	Gel 12	Run XhoI			Gel 26	*P. meandrina*	*P. meandrina*
7	Experiment	Gel 42	*P. acuta*							*P. acuta*
8	Experiment	Gel 42	Run EcoRV	Gel 37	Run XhoI			Gel 26	*P. meandrina*	*P. meandrina*
9	Experiment	Gel 1	Run EcoRV	Gel 12	Run XhoI			Gel 26	*P. meandrina*	*P. meandrina*
10	Experiment	Gel 1	Run EcoRV	Gel 12	Run XhoI			Gel 26	*P. meandrina*	*P. meandrina*
12	Experiment	Gel 1	Run Acil-BseYI			Gel 23	*P. tuahiniensis*			*P. tuahiniensis*
14	Experiment	Gel 1	Run EcoRV	Gel 12	Run XhoI			Gel 26	*P. meandrina*	*P. meandrina*
15	Experiment	Gel 1	Run Acil-BseYI			Gel 23	*P. tuahiniensis*			*P. tuahiniensis*
16	Experiment	Gel 1	Run EcoRV	Gel 12	Run XhoI			Gel 26	*P. meandrina*	*P. meandrina*
17	Experiment	Gel 42	Run EcoRV	Gel 37	Run XhoI			Gel 26	*P. meandrina*	*P. meandrina*
18	Experiment	Gel 42	Run EcoRV	Gel 37	Run XhoI			Gel 26	*P. meandrina*	*P. meandrina*
19	Experiment	Gel 1	Run EcoRV	Gel 12	Run XhoI			Gel 26	*P. meandrina*	*P. meandrina*
20	Experiment	Gel 1	Run EcoRV	Gel 12	Run XhoI			Gel 26	*P. meandrina*	*P. meandrina*
21	Experiment	Gel 1	Run EcoRV	Gel 12	Run XhoI			Gel 26	*P. meandrina*	*P. meandrina*
22	Experiment	Gel 1	Run EcoRV	Gel 12	Run XhoI			Gel 26	*P. meandrina*	*P. meandrina*
23	Experiment	Gel 1	Run EcoRV	Gel 12	Run XhoI			Gel 26	*P. grandis*	*P. grandis*
24	Experiment	Gel 1	Run Acil-BseYI			Gel 28	*P. tuahiniensis*			*P. tuahiniensis*
25	Experiment	Gel 1	Run EcoRV	Gel 12	Run XhoI			Gel 26	*P. meandrina*	*P. meandrina*
26	Experiment	Gel 1	Run EcoRV	Gel 12	Run XhoI			Gel 26	*P. meandrina*	*P. meandrina*
27	Experiment	Gel 1	*P. acuta*							*P. acuta*
28	Experiment	Gel 1	Run EcoRV	Gel 12	Run XhoI			Gel 26	*P. meandrina*	*P. meandrina*
29	Experiment	Gel 1	Run EcoRV	Gel 12	Run XhoI			Gel 26	*P. meandrina*	*P. meandrina*
30	Experiment	Gel 1	Run EcoRV	Gel 12	Run XhoI			Gel 26	*P. meandrina*	*P. meandrina*
31	Experiment	Gel 1	Run EcoRV	Gel 12	Run XhoI			Gel 26	*P. meandrina*	*P. meandrina*
33	Experiment	Gel 1	Run EcoRV	Gel 12	Run XhoI			Gel 26	*P. grandis*	*P. grandis*
34	Experiment	Gel 1	Run EcoRV	Gel 12	Run XhoI			Gel 26	*P. meandrina*	*P. meandrina*
35	Experiment	Gel 10	Run EcoRV	Gel 12	Run XhoI			Gel 26	*P. meandrina*	*P. meandrina*
36	Experiment	Gel 10	Run EcoRV	Gel 12	Run XhoI			Gel 26	*P. meandrina*	*P. meandrina*
37	Experiment	Gel 10	Run EcoRV	Gel 12	Run XhoI			Gel 26	*P. meandrina*	*P. meandrina*
38	Experiment	Gel 10	Run EcoRV	Gel 12	Run XhoI			Gel 26	*P. meandrina*	*P. meandrina*
39	Experiment	Gel 10	Run EcoRV	Gel 12	Run XhoI			Gel 26	*P. meandrina*	*P. meandrina*
40	Experiment	Gel 10	Run EcoRV	Gel 12	Run XhoI			Gel 40	*P. meandrina*	*P. meandrina*
41	Experiment	Gel 1	Run Acil-BseYI			Gel 23	*P. verrucosa*			*P. verrucosa*
42	Experiment	Gel 1	Run EcoRV	Gel 12	Run XhoI			Gel 40	*P. grandis*	*P. grandis*
43	Experiment	Gel 1	Run EcoRV	Gel 12	Run XhoI			Gel 26	*P. meandrina*	*P. meandrina*
44	Experiment	Gel 1	Run EcoRV	Gel 12	Run XhoI			Gel 26	*P. meandrina*	*P. meandrina*
45	Experiment	Gel 1	Run Acil-BseYI			Gel 28	*P. tuahiniensis*			*P. tuahiniensis*
46	Experiment	Gel 1	Run EcoRV	Gel 12	Run XhoI			Gel 40	*P. meandrina*	*P. meandrina*
47	Experiment	Gel 1	Run EcoRV	Gel 12	Run XhoI			Gel 31	*P. meandrina*	*P. meandrina*
48	Experiment	Gel 1	Run EcoRV	Gel 12	Run XhoI			Gel 31	*P. meandrina*	*P. meandrina*
49	Experiment	Gel 1	Run EcoRV	Gel 12	Run XhoI			Gel 31	*P. meandrina*	*P. meandrina*
50	Experiment	Gel 2	Run EcoRV	Gel 12	Run XhoI			Gel 31	*P. meandrina*	*P. meandrina*
51	Experiment	Gel 2	Run EcoRV	Gel 12	Run XhoI			Gel 31	*P. grandis*	*P. grandis*
52	Experiment	Gel 2	Run EcoRV	Gel 12	Run XhoI			Gel 31	*P. meandrina*	*P. meandrina*
53	Experiment	Gel 2	Run EcoRV	Gel 12	Run XhoI			Gel 31	*P. meandrina*	*P. meandrina*
54	Experiment	Gel 2	Run EcoRV	Gel 12	Run XhoI			Gel 27	*P. meandrina*	*P. meandrina*
55	Experiment	Gel 2	Run EcoRV	Gel 12	Run XhoI			Gel 27	*P. meandrina*	*P. meandrina*
56	Experiment	Gel 2	Run EcoRV	Gel 12	Run XhoI			Gel 27	*P. meandrina*	*P. meandrina*
58	Experiment	Gel 2	Run EcoRV	Gel 12	Run XhoI			Gel 27	*P. meandrina*	*P. meandrina*
59	Experiment	Gel 2	Run EcoRV	Gel 13	Run XhoI			Gel 27	*P. meandrina*	*P. meandrina*
60	Experiment	Gel 2	Run EcoRV	Gel 13	Run XhoI			Gel 27	*P. meandrina*	*P. meandrina*
62	Experiment	Gel 2	Run EcoRV	Gel 13	Run XhoI			Gel 27	*P. meandrina*	*P. meandrina*
63	Experiment	Gel 2	Run EcoRV	Gel 13	Run XhoI			Gel 27	*P. meandrina*	*P. meandrina*
64	Experiment	Gel 2	Run EcoRV	Gel 13	Run XhoI			Gel 27	*P. meandrina*	*P. meandrina*
65	Experiment	Gel 2	Run EcoRV	Gel 13	Run XhoI			Gel 27	*P. meandrina*	*P. meandrina*
66	Experiment	Gel 2	Run EcoRV	Gel 13	Run XhoI			Gel 27	*P. meandrina*	*P. meandrina*
67	Experiment	Gel 2	Run EcoRV	Gel 13	Run XhoI			Gel 27	*P. meandrina*	*P. meandrina*
68	Experiment	Gel 2	Run EcoRV	Gel 13	Run XhoI			Gel 27	*P. meandrina*	*P. meandrina*
69	Experiment	Gel 2	Run EcoRV	Gel 13	Run XhoI			Gel 27	*P. meandrina*	*P. meandrina*
70	Experiment	Gel 2	Run EcoRV	Gel 13	Run XhoI			Gel 27	*P. meandrina*	*P. meandrina*
72	Experiment	Gel 2	*P. acuta*							*P. acuta*
73	Experiment	Gel 2	Run EcoRV	Gel 13	Run XhoI			Gel 27	*P. meandrina*	*P. meandrina*
74	Experiment	Gel 2	Run EcoRV	Gel 13	Run XhoI			Gel 27	*P. meandrina*	*P. meandrina*
75	Experiment	Gel 2	Run EcoRV	Gel 13	Run XhoI			Gel 27	*P. meandrina*	*P. meandrina*
76	Experiment	Gel 2	Run EcoRV	Gel 13	Run XhoI			Gel 27	*P. meandrina*	*P. meandrina*
77	Experiment	Gel 2	Run EcoRV	Gel 13	Run XhoI			Gel 27	*P. meandrina*	*P. meandrina*
78	Experiment	Gel 2	Run EcoRV	Gel 13	Run XhoI			Gel 27	*P. meandrina*	*P. meandrina*
79	Experiment	Gel 2	Run Acil-BseYI			Gel 28	*P. tuahiniensis*			*P. tuahiniensis*
80	Experiment	Gel 2	Run EcoRV	Gel 37	Run XhoI			Gel 40	*P. meandrina*	*P. meandrina*
81	Experiment	Gel 2	Run EcoRV	Gel 13	Run XhoI			Gel 27	*P. meandrina*	*P. meandrina*

82	Experiment	Gel 2	Run EcoRV	Gel 13	Run XhoI			Gel 27	*P. meandrina*	*P. meandrina*
84	Experiment	Gel 2	Run EcoRV	Gel 13	Run XhoI			Gel 27	*P. meandrina*	*P. meandrina*
85	Experiment	Gel 2	Run EcoRV	Gel 13	Run XhoI			Gel 27	*P. meandrina*	*P. meandrina*
86	Experiment	Gel 2	Run EcoRV	Gel 13	Run XhoI					No ID
87	Experiment	Gel 2	Run EcoRV	Gel 13	Run XhoI			Gel 27	*P. meandrina*	*P. meandrina*
88	Experiment	Gel 2	Run EcoRV	Gel 13	Run XhoI			Gel 27	*P. meandrina*	*P. meandrina*
89	Experiment	Gel 2	Run EcoRV	Gel 13	Run XhoI			Gel 27	*P. grandis*	*P. grandis*
90	Experiment	Gel 2	Run EcoRV	Gel 13	Run XhoI			Gel 27	*P. meandrina*	*P. meandrina*
91	Experiment	Gel 2	Run EcoRV	Gel 13	Run XhoI			Gel 27	*P. meandrina*	*P. meandrina*
92	Experiment	Gel 2	Run EcoRV	Gel 13	Run XhoI			Gel 27	*P. meandrina*	*P. meandrina*
94	Experiment	Gel 2	Run EcoRV	Gel 13	Run XhoI			Gel 27	*P. grandis*	*P. grandis*
95	Experiment	Gel 2	Run EcoRV	Gel 13	Run XhoI					No ID
96	Experiment	Gel 2	Run EcoRV	Gel 13	Run XhoI			Gel 27	*P. meandrina*	*P. meandrina*
97	Experiment	Gel 2	Run EcoRV	Gel 13	Run XhoI			Gel 27	*P. meandrina*	*P. meandrina*
98	Experiment	Gel 2	Run EcoRV	Gel 13	Run XhoI			Gel 27	*P. grandis*	*P. grandis*
99	Experiment	Gel 2	Run EcoRV	Gel 13	Run XhoI			Gel 27	*P. meandrina*	*P. meandrina*
100	Experiment	Gel 2	Run EcoRV	Gel 13	Run XhoI			Gel 27	*P. meandrina*	*P. meandrina*
101	Experiment	Gel 2	Run EcoRV	Gel 13	Run XhoI			Gel 27	*P. meandrina*	*P. meandrina*
102	Experiment	Gel 10	Run EcoRV	Gel 13	Run XhoI			Gel 27	*P. meandrina*	*P. meandrina*
103	Experiment	Gel 10	Run EcoRV	Gel 13	Run XhoI			Gel 27	*P. meandrina*	*P. meandrina*
104	Experiment	Gel 10	Run EcoRV	Gel 13	Run XhoI			Gel 27	*P. meandrina*	*P. meandrina*
106	Experiment	Gel 10	Run EcoRV	Gel 13	Run XhoI			Gel 29	*P. meandrina*	*P. meandrina*
107	Experiment	Gel 10	Run EcoRV	Gel 13	Run XhoI			Gel 29	*P. meandrina*	*P. meandrina*
108	Experiment	Gel 10	Run EcoRV	Gel 13	Run XhoI			Gel 29	*P. meandrina*	*P. meandrina*
109	Experiment	Gel 10	Run EcoRV	Gel 13	Run XhoI			Gel 29	*P. grandis*	*P. grandis*
110	Experiment	Gel 10	Run EcoRV	Gel 14	Run XhoI			Gel 29	*P. meandrina*	*P. meandrina*
111	Experiment	Gel 10	*P. acuta*							*P. acuta*
112	Experiment	Gel 10	Run EcoRV	Gel 14	Run XhoI			Gel 29	*P. meandrina*	*P. meandrina*
114	Experiment	Gel 10	Run EcoRV	Gel 14	Run XhoI			Gel 29	*P. meandrina*	*P. meandrina*
115	Experiment	Gel 10	Run EcoRV	Gel 14	Run XhoI			Gel 29	*P. meandrina*	*P. meandrina*
116	Experiment	Gel 10	Run EcoRV	Gel 14	Run XhoI			Gel 43	*P. meandrina*	*P. meandrina*
117	Experiment	Gel 10	Run EcoRV	Gel 14	Run XhoI			Gel 29	*P. meandrina*	*P. meandrina*
118	Experiment	Gel 10	Run EcoRV	Gel 14	Run XhoI			Gel 29	*P. meandrina*	*P. meandrina*
119	Experiment	Gel 42	Run EcoRV	Gel 37	Run XhoI			Gel 40	*P. meandrina*	*P. meandrina*
124	Experiment	Gel 11	Run AciI-BseYI			Gel 23	*P. tuahiniensis*			*P. tuahiniensis*
125	Experiment	Gel 11	Run AciI-BseYI			Gel 23	*P. tuahiniensis*			*P. tuahiniensis*
126	Experiment	Gel 3	Run EcoRV	Gel 14	Run XhoI			Gel 41	*P. meandrina*	*P. meandrina*
127	Experiment	Gel 3	Run EcoRV					Gel 29		No ID
129	Experiment	Gel 3	Run EcoRV	Gel 37	Run XhoI			Gel 29	*P. meandrina*	*P. meandrina*
130	Experiment	Gel 3	Run EcoRV	Gel 37	Run XhoI			Gel 29	*P. meandrina*	*P. meandrina*
131	Experiment	Gel 3	Run EcoRV	Gel 37	Run XhoI			Gel 29	*P. meandrina*	*P. meandrina*
133	Experiment	Gel 42	*P. acuta*							*P. acuta*
134	Experiment	Gel 3	Run EcoRV	Gel 14	Run XhoI			Gel 29	*P. meandrina*	*P. meandrina*
135	Experiment	Gel 3	Run EcoRV	Gel 14	Run XhoI			Gel 29	*P. meandrina*	*P. meandrina*
136	Experiment	Gel 3	Run EcoRV	Gel 14	Run XhoI			Gel 29	*P. meandrina*	*P. meandrina*
137	Experiment	Gel 3	Run EcoRV	Gel 14	Run XhoI			Gel 29	*P. meandrina*	*P. meandrina*
138	Experiment	Gel 44	Run EcoRV	Gel 44	Run XhoI					No ID
140	Experiment	Gel 3	Run EcoRV	Gel 14	Run XhoI			Gel 29	*P. meandrina*	*P. meandrina*
141	Experiment	Gel 3	Run EcoRV	Gel 14	*P. effusa*					*P. effusa*
142	Experiment	Gel 44	Run EcoRV	Gel 44	Run XhoI					No ID
143	Experiment	Gel 3	Run EcoRV	Gel 14	Run XhoI			Gel 29	*P. meandrina*	*P. meandrina*
144	Experiment	Gel 3	Run EcoRV	Gel 14	Run XhoI			Gel 29	*P. meandrina*	*P. meandrina*
145	Experiment	Gel 44	Run EcoRV	Gel 44	Run XhoI					No ID
146	Experiment	Gel 3	Run EcoRV	Gel 14	Run XhoI			Gel 29	*P. meandrina*	*P. meandrina*
147	Experiment	Gel 44	Run EcoRV	Gel 44	Run XhoI					No ID
148	Experiment	Gel 3	Run EcoRV	Gel 14	Run XhoI			Gel 29	*P. grandis*	*P. grandis*
149	Experiment	Gel 3	Run EcoRV	Gel 14	Run XhoI			Gel 29	*P. meandrina*	*P. meandrina*
150	Experiment	Gel 3	Run EcoRV	Gel 14	Run XhoI			Gel 29	*P. meandrina*	*P. meandrina*
151	Experiment	Gel 3	Run EcoRV	Gel 14	Run XhoI			Gel 29	*P. meandrina*	*P. meandrina*
152	Experiment	Gel 3	Run EcoRV	Gel 14	Run XhoI			Gel 29	*P. meandrina*	*P. meandrina*
153	Experiment	Gel 3	Run EcoRV	Gel 14	Run XhoI			Gel 29	*P. meandrina*	*P. meandrina*
154	Experiment	Gel 3	Run EcoRV	Gel 14	Run XhoI			Gel 29	*P. meandrina*	*P. meandrina*
155	Experiment	Gel 44	Run EcoRV	Gel 44	*P. effusa*					*P. effusa*
156	Experiment	Gel 3	Run EcoRV	Gel 14	Run XhoI			Gel 29	*P. meandrina*	*P. meandrina*
157	Experiment	Gel 3	Run EcoRV	Gel 14	Run XhoI			Gel 29	*P. grandis*	*P. grandis*
158	Experiment	Gel 3	Run EcoRV	Gel 14	Run XhoI			Gel 29	*P. grandis*	*P. grandis*
160	Experiment	Gel 3	Run EcoRV	Gel 14	Run XhoI			Gel 29	*P. meandrina*	*P. meandrina*
164	Experiment	Gel 3	Run EcoRV	Gel 14	Run XhoI			Gel 29	*P. meandrina*	*P. meandrina*
165	Experiment	Gel 3	Run EcoRV	Gel 14	Run XhoI			Gel 29	*P. meandrina*	*P. meandrina*
168	Experiment	Gel 3	Run EcoRV	Gel 14	Run XhoI			Gel 29	*P. meandrina*	*P. meandrina*
169	Experiment	Gel 3	Run EcoRV	Gel 14	Run XhoI			Gel 29	*P. meandrina*	*P. meandrina*
170	Experiment	Gel 3	Run EcoRV	Gel 14	Run XhoI			Gel 29	*P. meandrina*	*P. meandrina*
177	Experiment	Gel 3	Run EcoRV	Gel 14	Run XhoI			Gel 29	*P. meandrina*	*P. meandrina*
178	Experiment	Gel 3	Run EcoRV	Gel 15	Run XhoI			Gel 29	*P. meandrina*	*P. meandrina*
179	Experiment	Gel 3	Run EcoRV	Gel 15	Run XhoI			Gel 29	*P. meandrina*	*P. meandrina*
180	Experiment	Gel 3	Run EcoRV	Gel 14	Run XhoI			Gel 30		No ID
181	Experiment	Gel 3	Run EcoRV	Gel 14	Run XhoI			Gel 30	*P. meandrina*	*P. meandrina*
182	Experiment	Gel 3	Run EcoRV	Gel 14	Run XhoI			Gel 30	*P. meandrina*	*P. meandrina*
183	Experiment	Gel 4	Run EcoRV	Gel 14	Run XhoI			Gel 30	*P. meandrina*	*P. meandrina*
184	Experiment	Gel 4	Run EcoRV	Gel 15	Run XhoI			Gel 30	*P. meandrina*	*P. meandrina*
185	Experiment	Gel 4	Run EcoRV	Gel 15	Run XhoI			Gel 30	*P. meandrina*	*P. meandrina*

186	Experiment	Gel 4	Run EcoRV	Gel 15	Run XhoI			Gel 30	*P. meandrina*	*P. meandrina*
187	Experiment	Gel 4	Run AciI-BseYI			Gel 23	*P. tuahiniensis*			*P. tuahiniensis*
188	Experiment	Gel 4	Run EcoRV	Gel 15	Run XhoI			Gel 30	*P. meandrina*	*P. meandrina*
189	Experiment	Gel 4	Run EcoRV	Gel 15	Run XhoI			Gel 30	*P. meandrina*	*P. meandrina*
190	Experiment	Gel 4	Run EcoRV	Gel 15	Run XhoI			Gel 30	*P. meandrina*	*P. meandrina*
191	Experiment	Gel 4	Run EcoRV	Gel 15	Run XhoI			Gel 30	*P. meandrina*	*P. meandrina*
192	Experiment	Gel 4	Run EcoRV	Gel 15	Run XhoI			Gel 30	*P. meandrina*	*P. meandrina*
193	Experiment	Gel 4	Run EcoRV	Gel 15	Run XhoI			Gel 30	*P. meandrina*	*P. meandrina*
194	Experiment	Gel 4	Run EcoRV	Gel 15	Run XhoI			Gel 30	*P. meandrina*	*P. meandrina*
195	Experiment	Gel 4	Run EcoRV	Gel 15	Run XhoI			Gel 30	*P. meandrina*	*P. meandrina*
196	Experiment	Gel 4	Run EcoRV	Gel 15	Run XhoI			Gel 30	*P. meandrina*	*P. meandrina*
197	Experiment	Gel 4	Run EcoRV	Gel 15	Run XhoI			Gel 30	*P. meandrina*	*P. meandrina*
198	Experiment	Gel 4	Run EcoRV	Gel 15	Run XhoI			Gel 30	*P. meandrina*	*P. meandrina*
199	Experiment	Gel 4	Run EcoRV	Gel 15	Run XhoI			Gel 30	*P. meandrina*	*P. meandrina*
200	Experiment	Gel 4	Run EcoRV	Gel 15	Run XhoI			Gel 30	*P. meandrina*	*P. meandrina*
201	Experiment	Gel 4	Run EcoRV	Gel 15	Run XhoI			Gel 30	*P. meandrina*	*P. meandrina*
202	Experiment	Gel 4	Run EcoRV	Gel 15	Run XhoI			Gel 30	*P. meandrina*	*P. meandrina*
203	Experiment	Gel 4	Run EcoRV	Gel 15	Run XhoI			Gel 30	*P. meandrina*	*P. meandrina*
204	Experiment	Gel 4	Run EcoRV	Gel 15	Run XhoI			Gel 30	*P. meandrina*	*P. meandrina*
205	Experiment	Gel 4	Run EcoRV	Gel 15	Run XhoI			Gel 30	*P. meandrina*	*P. meandrina*
206	Experiment	Gel 4	Run EcoRV	Gel 15	Run XhoI			Gel 30	*P. meandrina*	*P. meandrina*
207	Experiment	Gel 4	Run AciI-BseYI			Gel 43	*P. tuahiniensis*			*P. tuahiniensis*
208	Experiment	Gel 4	Run AciI-BseYI			Gel 23	*P. tuahiniensis*			*P. tuahiniensis*
209	Experiment	Gel 4	Run EcoRV	Gel 15	Run XhoI			Gel 30	*P. grandis*	*P. grandis*
210	Experiment	Gel 4	Run EcoRV	Gel 15	Run XhoI			Gel 30	*P. meandrina*	*P. meandrina*
211	Experiment	Gel 4	Run EcoRV	Gel 15	Run XhoI			Gel 30	*P. meandrina*	*P. meandrina*
212	Experiment	Gel 4	Run EcoRV	Gel 15	Run XhoI			Gel 30	*P. meandrina*	*P. meandrina*
213	Experiment	Gel 4	Run EcoRV	Gel 15	Run XhoI			Gel 30	*P. grandis*	*P. grandis*
214	Experiment	Gel 4	Run AciI-BseYI			Gel 23	*P. tuahiniensis*			*P. tuahiniensis*
216	Experiment	Gel 4	Run EcoRV	Gel 15	Run XhoI			Gel 30	*P. meandrina*	*P. meandrina*
217	Experiment	Gel 4	Run EcoRV	Gel 15	Run XhoI			Gel 30	*P. meandrina*	*P. meandrina*
218	Experiment	Gel 4	Run AciI-BseYI			Gel 23	*P. tuahiniensis*			*P. tuahiniensis*
219	Experiment			Gel 37	Run XhoI					No ID
220	Experiment	Gel 4	Run EcoRV	Gel 15	Run XhoI			Gel 30	*P. meandrina*	*P. meandrina*
221	Experiment	Gel 4	Run EcoRV	Gel 15	Run XhoI			Gel 30	*P. meandrina*	*P. meandrina*
222	Experiment	Gel 11	Run EcoRV	Gel 15	Run XhoI			Gel 30	*P. grandis*	*P. grandis*
223	Experiment	Gel 4	Run EcoRV	Gel 15	Run XhoI			Gel 30	*P. meandrina*	*P. meandrina*
224	Experiment	Gel 4	Run EcoRV	Gel 15	Run XhoI			Gel 30	*P. meandrina*	*P. meandrina*
225	Experiment	Gel 4	Run EcoRV	Gel 15	Run XhoI			Gel 30	*P. meandrina*	*P. meandrina*
226	Experiment	Gel 5	Run EcoRV	Gel 15	Run XhoI			Gel 30	*P. grandis*	*P. grandis*
227	Experiment	Gel 5	Run EcoRV	Gel 15	Run XhoI			Gel 30	*P. meandrina*	*P. meandrina*
228	Experiment	Gel 5	Run EcoRV	Gel 15	Run XhoI			Gel 30	*P. meandrina*	*P. meandrina*
229	Experiment	Gel 5	Run EcoRV	Gel 15	Run XhoI			Gel 30	*P. meandrina*	*P. meandrina*
230	Experiment	Gel 11	Run EcoRV	Gel 15	Run XhoI					No ID
231	Experiment	Gel 5	*P. acuta*							*P. acuta*
232	Experiment	Gel 5	Run EcoRV	Gel 15	Run XhoI			Gel 31	*P. meandrina*	*P. meandrina*
233	Experiment	Gel 5	Run EcoRV					Gel 31	*P. grandis*	*P. grandis*
234	Experiment	Gel 5	Run EcoRV	Gel 16	Run XhoI			Gel 31	*P. meandrina*	*P. meandrina*
235	Experiment	Gel 5	Run EcoRV	Gel 16	Run XhoI			Gel 31	*P. meandrina*	*P. meandrina*
236	Experiment	Gel 5	Run EcoRV	Gel 16	Run XhoI			Gel 31	*P. meandrina*	*P. meandrina*
237	Experiment	Gel 5	Run EcoRV	Gel 16	Run XhoI			Gel 31	*P. meandrina*	*P. meandrina*
238	Experiment	Gel 5	Run EcoRV	Gel 16	Run XhoI			Gel 31	*P. grandis*	*P. grandis*
239	Experiment	Gel 5	Run EcoRV	Gel 16	Run XhoI			Gel 31	*P. meandrina*	*P. meandrina*
242	Experiment	Gel 5	Run EcoRV	Gel 16	Run XhoI			Gel 31	*P. meandrina*	*P. meandrina*
244	Experiment	Gel 5	Run EcoRV	Gel 25	Run XhoI			Gel 40	*P. grandis*	*P. grandis*
245	Experiment	Gel 11	*P. acuta*							*P. acuta*
247	Experiment	Gel 5	Run AciI-BseYI			Gel 23	*P. tuahiniensis*			*P. tuahiniensis*
248	Experiment	Gel 5	Run EcoRV	Gel 16	Run XhoI			Gel 31	*P. meandrina*	*P. meandrina*
249	Experiment	Gel 5	Run EcoRV	Gel 16	Run XhoI			Gel 31	*P. grandis*	*P. grandis*
250	Experiment	Gel 5	Run EcoRV	Gel 16	Run XhoI			Gel 31	*P. meandrina*	*P. meandrina*
251	Experiment	Gel 11	Run EcoRV	Gel 16	Blank			Gel 31	*P. grandis*	*P. grandis*
252	Experiment	Gel 5	Run AciI-BseYI			Gel 23	*P. tuahiniensis*			*P. tuahiniensis*
253	Experiment	Gel 5	Run AciI-BseYI			Gel 23	*P. tuahiniensis*			*P. tuahiniensis*
254	Experiment	Gel 11	Run EcoRV	Gel 37	Run XhoI			Gel 31	*P. meandrina*	*P. meandrina*
255	Experiment	Gel 5	Run AciI-BseYI			Gel 23	*P. tuahiniensis*			*P. tuahiniensis*
256	Experiment	Gel 11	Run EcoRV	Gel 16	Run XhoI			Gel 31	*P. meandrina*	*P. meandrina*
257	Experiment	Gel 11	Run EcoRV	Gel 37	Run XhoI			Gel 31	*P. meandrina*	*P. meandrina*
258	Experiment	Gel 5	Run AciI-BseYI			Gel 23	*P. tuahiniensis*			*P. tuahiniensis*
259	Experiment	Gel 11	Run EcoRV	Gel 16	Run XhoI			Gel 31	*P. meandrina*	*P. meandrina*
260	Experiment	Gel 11	Run EcoRV	Gel 16	Run XhoI			Gel 32	*P. grandis*	*P. grandis*
261	Experiment	Gel 5	Run AciI-BseYI			Gel 23	*P. verrucosa*			*P. verrucosa*
262	Experiment	Gel 11	Run EcoRV	Gel 16	Run XhoI			Gel 32	*P. meandrina*	*P. meandrina*
263	Experiment	Gel 11	Run EcoRV	Gel 16	Run XhoI			Gel 32	*P. meandrina*	*P. meandrina*
264	Experiment	Gel 5	Run AciI-BseYI			Gel 23	*P. tuahiniensis*			*P. tuahiniensis*
265	Experiment	Gel 11	Run EcoRV	Gel 16	Run XhoI			Gel 32	*P. meandrina*	*P. meandrina*
266	Experiment	Gel 11	Run EcoRV	Gel 16	Run XhoI			Gel 32	*P. grandis*	*P. grandis*
267	Experiment	Gel 11	Run EcoRV	Gel 16	Run XhoI			Gel 32	*P. grandis*	*P. grandis*
268	Experiment	Gel 11	Run EcoRV	Gel 16	Run XhoI			Gel 32	*P. meandrina*	*P. meandrina*
269	Experiment	Gel 11	Run EcoRV	Gel 16	Run XhoI			Gel 32	*P. grandis*	*P. grandis*
271	Experiment	Gel 11	Run EcoRV	Gel 16	Run XhoI			Gel 32	*P. meandrina*	*P. meandrina*
272	Experiment	Gel 5	Run AciI-BseYI			Gel 23	*P. verrucosa*			*P. verrucosa*

273	Experiment	Gel 11	Run EcoRV	Gel 16	Run XhoI			Gel 32	*P. meandrina*	*P. meandrina*
274	Experiment	Gel 6	Run AciI-BseYI			Gel 23	*P. tuahiniensis*			*P. tuahiniensis*
275	Experiment	Gel 11	Run EcoRV	Gel 16	Run XhoI			Gel 32	*P. grandis*	*P. grandis*
276	Experiment	Gel 11	Run EcoRV	Gel 16	Run XhoI			Gel 32	*P. meandrina*	*P. meandrina*
277	Experiment	Gel 11	Run EcoRV	Gel 16	Run XhoI			Gel 32	*P. meandrina*	*P. meandrina*
278	Experiment	Gel 11	Run EcoRV	Gel 16	Run XhoI			Gel 32	*P. meandrina*	*P. meandrina*
279	Experiment	Gel 6	Run AciI-BseYI			Gel 23	*P. tuahiniensis*			*P. tuahiniensis*
280	Experiment	Gel 11	Run EcoRV	Gel 16	Run XhoI			Gel 32	*P. meandrina*	*P. meandrina*
281	Experiment	Gel 6	Run AciI-BseYI			Gel 23	*P. verrucosa*			*P. verrucosa*
282	Experiment	Gel 11	Run EcoRV	Gel 16	Run XhoI			Gel 32	*P. meandrina*	*P. meandrina*
283	Experiment	Gel 11	Run EcoRV	Gel 16	Run XhoI			Gel 32	*P. meandrina*	*P. meandrina*
284	Experiment	Gel 6	Run AciI-BseYI			Gel 23	*P. tuahiniensis*			*P. tuahiniensis*
285	Experiment	Gel 11	Run EcoRV	Gel 16	Run XhoI			Gel 32	*P. meandrina*	*P. meandrina*
286	Experiment	Gel 11	Run EcoRV	Gel 16	Run XhoI			Gel 32	*P. meandrina*	*P. meandrina*
288	Experiment	Gel 11	Run EcoRV	Gel 16	Run XhoI			Gel 32	*P. meandrina*	*P. meandrina*
289	Experiment	Gel 11	Run EcoRV	Gel 16	Run XhoI			Gel 32	*P. meandrina*	*P. meandrina*
290	Experiment	Gel 11	Run EcoRV	Gel 16	Run XhoI			Gel 32	*P. meandrina*	*P. meandrina*
291	Experiment	Gel 11	Run EcoRV	Gel 16	Run XhoI			Gel 32	*P. meandrina*	*P. meandrina*
293	Experiment	Gel 11	Run EcoRV	Gel 16	Run XhoI			Gel 32	*P. meandrina*	*P. meandrina*
295	Experiment	Gel 22	Run EcoRV	Gel 16	Run XhoI			Gel 32	*P. meandrina*	*P. meandrina*
296	Experiment	Gel 6	Run AciI-BseYI			Gel 23	*P. verrucosa*			*P. verrucosa*
297	Experiment	Gel 20	Run EcoRV	Gel 16	Run XhoI			Gel 32	*P. meandrina*	*P. meandrina*
298	Experiment	Gel 20	Run EcoRV	Gel 16	Run XhoI			Gel 32	*P. meandrina*	*P. meandrina*
299	Experiment	Gel 20	Run EcoRV	Gel 17	Run XhoI			Gel 32	*P. meandrina*	*P. meandrina*
300	Experiment	Gel 20	Run EcoRV	Gel 17	Run XhoI			Gel 32	*P. meandrina*	*P. meandrina*
301	Experiment	Gel 20	Run EcoRV	Gel 17	Run XhoI			Gel 32	*P. meandrina*	*P. meandrina*
302	Experiment	Gel 20	Run EcoRV	Gel 17	Run XhoI			Gel 32	*P. meandrina*	*P. meandrina*
303	Experiment	Gel 42	Run EcoRV	Gel 17	Run XhoI			Gel 32	*P. meandrina*	*P. meandrina*
304	Experiment	Gel 20	Run EcoRV	Gel 17	Run XhoI			Gel 32	*P. grandis*	*P. grandis*
305	Experiment	Gel 20	Run AciI-BseYI			Gel 23	*P. tuahiniensis*			*P. tuahiniensis*
306	Experiment	Gel 42	Run EcoRV	Gel 17	Run XhoI			Gel 41	*P. grandis*	*P. grandis*
307	Experiment	Gel 20	Run EcoRV	Gel 17	Run XhoI			Gel 32	*P. meandrina*	*P. meandrina*
308	Experiment	Gel 42	Run EcoRV	Gel 17	Run XhoI			Gel 32	*P. meandrina*	*P. meandrina*
309	Experiment	Gel 42	Run EcoRV	Gel 17	Run XhoI			Gel 32	*P. meandrina*	*P. meandrina*
310	Experiment	Gel 42	Run EcoRV	Gel 17	Run XhoI			Gel 32	*P. meandrina*	*P. meandrina*
311	Experiment	Gel 20	Run EcoRV	Gel 17	Run XhoI			Gel 32	*P. meandrina*	*P. meandrina*
313	Experiment	Gel 20	Run EcoRV	Gel 17	Run XhoI			Gel 32	*P. meandrina*	*P. meandrina*
314	Experiment	Gel 20	Run EcoRV	Gel 17	Run XhoI			Gel 32	*P. meandrina*	*P. meandrina*
315	Experiment	Gel 20	Run EcoRV	Gel 17	Run XhoI			Gel 32	*P. meandrina*	*P. meandrina*
316	Experiment	Gel 20	Run EcoRV	Gel 17	Run XhoI			Gel 32	*P. meandrina*	*P. meandrina*
317	Experiment	Gel 20	Run EcoRV	Gel 17	Run XhoI			Gel 32	*P. meandrina*	*P. meandrina*
318	Experiment	Gel 20	Run EcoRV	Gel 17	Run XhoI			Gel 33	*P. meandrina*	*P. meandrina*
319	Experiment	Gel 7	Run EcoRV	Gel 17	Run XhoI			Gel 33	*P. meandrina*	*P. meandrina*
320	Experiment	Gel 7	Run EcoRV	Gel 17	Run XhoI			Gel 33	*P. meandrina*	*P. meandrina*
321	Experiment	Gel 7	Run EcoRV	Gel 17	Run XhoI			Gel 33	*P. meandrina*	*P. meandrina*
322	Experiment	Gel 7	Run EcoRV	Gel 17	Run XhoI			Gel 33	*P. meandrina*	*P. meandrina*
323	Experiment	Gel 7	Run EcoRV	Gel 17	Run XhoI			Gel 33	*P. meandrina*	*P. meandrina*
324	Experiment	Gel 7	Run EcoRV	Gel 17	Run XhoI			Gel 33	*P. meandrina*	*P. meandrina*
326	Experiment	Gel 7	Run EcoRV	Gel 17	Run XhoI			Gel 33	*P. grandis*	*P. grandis*
327	Experiment	Gel 7	Run EcoRV	Gel 17	Run XhoI			Gel 33	*P. meandrina*	*P. meandrina*
328	Experiment	Gel 7	Run EcoRV	Gel 17	Run XhoI			Gel 33	*P. meandrina*	*P. meandrina*
329	Experiment	Gel 7	Run EcoRV	Gel 17	Run XhoI			Gel 33	*P. meandrina*	*P. meandrina*
330	Experiment	Gel 7	Run EcoRV	Gel 17	Run XhoI			Gel 33	*P. meandrina*	*P. meandrina*
331	Experiment	Gel 42	Run EcoRV	Gel 17	Run XhoI			Gel 33	*P. meandrina*	*P. meandrina*
333	Experiment	Gel 7	Run EcoRV	Gel 17	Run XhoI			Gel 33	*P. meandrina*	*P. meandrina*
334	Experiment	Gel 7	Run EcoRV	Gel 17	Run XhoI			Gel 33	*P. meandrina*	*P. meandrina*
335	Experiment	Gel 7	Run EcoRV	Gel 17	Run XhoI			Gel 33	*P. meandrina*	*P. meandrina*
337	Experiment	Gel 7	Run EcoRV	Gel 17	Run XhoI			Gel 33	*P. meandrina*	*P. meandrina*
338	Experiment	Gel 7	Run EcoRV	Gel 17	Run XhoI			Gel 33	*P. meandrina*	*P. meandrina*
340	Experiment	Gel 7	Run EcoRV	Gel 17	Run XhoI			Gel 33	*P. grandis*	*P. grandis*
341	Experiment	Gel 42	Run EcoRV	Gel 17	Run XhoI			Gel 33	*P. meandrina*	*P. meandrina*
342	Experiment	Gel 7	*P. acuta*							*P. acuta*
343	Experiment	Gel 7	Run EcoRV	Gel 17	Run XhoI			Gel 33	*P. grandis*	*P. grandis*
344	Experiment	Gel 7	Run EcoRV	Gel 17	Run XhoI			Gel 33	*P. meandrina*	*P. meandrina*
345	Experiment	Gel 7	Run AciI-BseYI			Gel 23	*P. tuahiniensis*			*P. tuahiniensis*
346	Experiment	Gel 20	Run EcoRV	Gel 17	*P. effusa*					*P. effusa*
348	Experiment	Gel 20	Run EcoRV	Gel 17	Run XhoI			Gel 33	*P. meandrina*	*P. meandrina*
349	Experiment	Gel 20	Run EcoRV	Gel 17	Run XhoI			Gel 33	*P. meandrina*	*P. meandrina*
350	Experiment	Gel 7	Run AciI-BseYI			Gel 23	*P. verrucosa*			*P. verrucosa*
351	Experiment	Gel 20	Run EcoRV	Gel 17	Run XhoI			Gel 33	*P. meandrina*	*P. meandrina*
353	Experiment	Gel 20	Run EcoRV	Gel 18	Run XhoI			Gel 33	*P. meandrina*	*P. meandrina*
354	Experiment	Gel 20	Run EcoRV	Gel 18	Run XhoI			Gel 33	*P. grandis*	*P. grandis*
355	Experiment	Gel 20	Run EcoRV	Gel 18	Run XhoI			Gel 33	*P. meandrina*	*P. meandrina*
356	Experiment	Gel 20	Run EcoRV	Gel 18	Run XhoI			Gel 33	*P. meandrina*	*P. meandrina*
357	Experiment	Gel 20	Run EcoRV	Gel 18	Run XhoI			Gel 33	*P. meandrina*	*P. meandrina*
358	Experiment	Gel 20	Run EcoRV	Gel 18	Run XhoI			Gel 33	*P. grandis*	*P. grandis*
359	Experiment	Gel 20	Run EcoRV	Gel 18	Run XhoI			Gel 33	*P. meandrina*	*P. meandrina*
360	Experiment	Gel 7	Run AciI-BseYI			Gel 23	*P. tuahiniensis*			*P. tuahiniensis*
361	Experiment	Gel 20	Run EcoRV	Gel 18	Run XhoI			Gel 33	*P. grandis*	*P. grandis*
362	Experiment	Gel 20	Run EcoRV	Gel 18	Run XhoI			Gel 33	*P. meandrina*	*P. meandrina*
363	Experiment	Gel 20	Run EcoRV	Gel 18	Run XhoI			Gel 33	*P. meandrina*	*P. meandrina*

367	Experiment	Gel 7	Run AciI-BseYI			Gel 23	*P. tuahiniensis*			*P. tuahiniensis*
368	Experiment	Gel 20	Run EcoRV	Gel 18	Run XhoI			Gel 33	*P. meandrina*	*P. meandrina*
369	Experiment	Gel 20	Run EcoRV	Gel 18	Run XhoI			Gel 33	*P. grandis*	*P. grandis*
370	Experiment	Gel 20	Run EcoRV	Gel 18	Run XhoI			Gel 33	*P. meandrina*	*P. meandrina*
371	Experiment	Gel 8	Run AciI-BseYI			Gel 23	*P. tuahiniensis*			*P. tuahiniensis*
372	Experiment	Gel 20	Run EcoRV	Gel 25	Run XhoI			Gel 33	*P. meandrina*	*P. meandrina*
373	Experiment	Gel 20	Run EcoRV	Gel 25	Run XhoI			Gel 33	*P. meandrina*	*P. meandrina*
374	Experiment	Gel 8	Run EcoRV	Gel 18	Run XhoI			Gel 33	*P. grandis*	*P. grandis*
375	Experiment	Gel 8	Run EcoRV	Gel 18	Run XhoI			Gel 33	*P. grandis*	*P. grandis*
376	Experiment	Gel 8	Run EcoRV	Gel 18	Run XhoI			Gel 33	*P. meandrina*	*P. meandrina*
378	Experiment	Gel 8	Run EcoRV	Gel 18	Run XhoI			Gel 34	*P. meandrina*	*P. meandrina*
379	Experiment	Gel 8	Run EcoRV							No ID
380	Experiment	Gel 8	Run EcoRV	Gel 18	Run XhoI			Gel 34	*P. meandrina*	*P. meandrina*
381	Experiment	Gel 42	Run EcoRV	Gel 18	Run XhoI			Gel 34	*P. meandrina*	*P. meandrina*
382	Experiment	Gel 42	Run EcoRV	Gel 18	Run XhoI			Gel 34	*P. meandrina*	*P. meandrina*
383	Experiment	Gel 42	Run EcoRV	Gel 18	Run XhoI			Gel 34	*P. meandrina*	*P. meandrina*
384	Experiment	Gel 8	Run EcoRV	Gel 18	Run XhoI			Gel 34	*P. meandrina*	*P. meandrina*
385	Experiment	Gel 8	Run EcoRV	Gel 18	Run XhoI			Gel 34	*P. grandis*	*P. grandis*
387	Experiment	Gel 8	Run AciI-BseYI			Gel 23	*P. tuahiniensis*			*P. tuahiniensis*
390	Experiment	Gel 8	Run EcoRV	Gel 18	Run XhoI			Gel 34	*P. meandrina*	*P. meandrina*
391	Experiment	Gel 8	Run EcoRV	Gel 18	Run XhoI			Gel 34	*P. meandrina*	*P. meandrina*
392	Experiment	Gel 8	Run EcoRV	Gel 18	Run XhoI			Gel 34	*P. meandrina*	*P. meandrina*
393	Experiment	Gel 8	Run EcoRV	Gel 18	Run XhoI			Gel 34	*P. meandrina*	*P. meandrina*
395	Experiment	Gel 8	Run EcoRV	Gel 18	Run XhoI					No ID
396	Experiment	Gel 8	Run AciI-BseYI			Gel 23	*P. tuahiniensis*			*P. tuahiniensis*
397	Experiment	Gel 8	Run AciI-BseYI			Gel 23	*P. tuahiniensis*			*P. tuahiniensis*
398	Experiment	Gel 8	Run EcoRV	Gel 18	Run XhoI			Gel 34	*P. meandrina*	*P. meandrina*
400	Experiment	Gel 8	Run EcoRV	Gel 18	Run XhoI			Gel 34	*P. meandrina*	*P. meandrina*
401	Experiment	Gel 8	Run EcoRV	Gel 18	Run XhoI			Gel 34	*P. meandrina*	*P. meandrina*
402	Experiment	Gel 8	Run EcoRV	Gel 18	Run XhoI			Gel 34	*P. meandrina*	*P. meandrina*
403	Experiment	Gel 8	Run EcoRV	Gel 18	Run XhoI			Gel 34	*P. meandrina*	*P. meandrina*
404	Experiment	Gel 8	Run EcoRV	Gel 18	Run XhoI			Gel 34	*P. meandrina*	*P. meandrina*
405	Experiment	Gel 8	Run AciI-BseYI			Gel 23	*P. tuahiniensis*			*P. tuahiniensis*
407	Experiment	Gel 8	Run EcoRV	Gel 18	Run XhoI			Gel 34	*P. meandrina*	*P. meandrina*
408	Experiment	Gel 8	Run EcoRV	Gel 18	Run XhoI			Gel 34	*P. meandrina*	*P. meandrina*
411	Experiment	Gel 8	Run EcoRV	Gel 18	Run XhoI			Gel 34	*P. meandrina*	*P. meandrina*
412	Experiment	Gel 8	Run EcoRV	Gel 18	Run XhoI			Gel 34	*P. meandrina*	*P. meandrina*
413	Experiment	Gel 8	Run EcoRV	Gel 18	Run XhoI			Gel 34	*P. meandrina*	*P. meandrina*
414	Experiment	Gel 8	Run EcoRV	Gel 18	Run XhoI			Gel 34	*P. meandrina*	*P. meandrina*
415	Experiment	Gel 8	Run AciI-BseYI			Gel 23	*P. verrucosa*			*P. verrucosa*
416	Experiment	Gel 8	Run EcoRV	Gel 18	Run XhoI			Gel 34	*P. meandrina*	*P. meandrina*
417	Experiment	Gel 8	Run EcoRV	Gel 18	Run XhoI			Gel 34	*P. meandrina*	*P. meandrina*
418	Experiment	Gel 8	Run EcoRV	Gel 18	Run XhoI			Gel 34	*P. meandrina*	*P. meandrina*
419	Experiment	Gel 8	Run EcoRV	Gel 18	*P. effusa*					*P. effusa*
421	Experiment	Gel 8	Run EcoRV	Gel 19	Run XhoI			Gel 34	*P. meandrina*	*P. meandrina*
422	Experiment	Gel 8	Run EcoRV	Gel 19	Run XhoI			Gel 34	*P. grandis*	*P. grandis*
423	Experiment	Gel 8	Run EcoRV	Gel 19	Run XhoI			Gel 34	*P. meandrina*	*P. meandrina*
424	Experiment	Gel 24	Run EcoRV	Gel 19	Run XhoI			Gel 34	*P. grandis*	*P. grandis*
425	Experiment	Gel 24	Run EcoRV	Gel 19	Run XhoI			Gel 34	*P. meandrina*	*P. meandrina*
426	Experiment	Gel 24	Run EcoRV	Gel 19	Run XhoI			Gel 34	*P. meandrina*	*P. meandrina*
427	Experiment	Gel 24	Run EcoRV	Gel 19	Run XhoI			Gel 34	*P. meandrina*	*P. meandrina*
428	Experiment	Gel 24	Run EcoRV	Gel 19	Run XhoI			Gel 34	*P. meandrina*	*P. meandrina*
429	Experiment	Gel 24	Run EcoRV	Gel 19	Run XhoI			Gel 34	*P. meandrina*	*P. meandrina*
430	Experiment	Gel 9	Run AciI-BseYI			Gel 23	*P. tuahiniensis*			*P. tuahiniensis*
431	Experiment	Gel 24	Run EcoRV	Gel 19	Run XhoI			Gel 34	*P. meandrina*	*P. meandrina*
432	Experiment	Gel 24	Run EcoRV	Gel 19	Run XhoI			Gel 34	*P. meandrina*	*P. meandrina*
433	Experiment	Gel 24	Run EcoRV	Gel 19	Run XhoI			Gel 35	*P. meandrina*	*P. meandrina*
435	Experiment	Gel 24	Run EcoRV	Gel 19	Run XhoI			Gel 35	*P. meandrina*	*P. meandrina*
436	Experiment	Gel 24	Run EcoRV	Gel 19	Run XhoI			Gel 35	*P. meandrina*	*P. meandrina*
437	Experiment	Gel 24	Run EcoRV	Gel 19	Run XhoI			Gel 35	*P. meandrina*	*P. meandrina*
438	Experiment	Gel 24	Run EcoRV	Gel 19	Run XhoI			Gel 35	*P. meandrina*	*P. meandrina*
439	Experiment	Gel 42	Run EcoRV	Gel 19	Run XhoI			Gel 35	*P. meandrina*	*P. meandrina*
440	Experiment	Gel 42	Run EcoRV	Gel 19	Run XhoI			Gel 35	*P. grandis*	*P. grandis*
441	Experiment	Gel 42	Run EcoRV	Gel 19	Run XhoI			Gel 35	*P. meandrina*	*P. meandrina*
442	Experiment	Gel 42	Run EcoRV	Gel 19	Run XhoI			Gel 35	*P. meandrina*	*P. meandrina*
443	Experiment	Gel 42	Run EcoRV	Gel 19	Run XhoI			Gel 35	*P. meandrina*	*P. meandrina*
444	Experiment	Gel 42	Run EcoRV	Gel 19	Run XhoI			Gel 35	*P. meandrina*	*P. meandrina*
445	Experiment			Gel 19	Run XhoI			Gel 35		No ID
446	Experiment	Gel 9	Run EcoRV	Gel 19	Run XhoI			Gel 35	*P. meandrina*	*P. meandrina*
447	Experiment	Gel 9	Run EcoRV	Gel 19	Run XhoI			Gel 35	*P. meandrina*	*P. meandrina*
448	Experiment	Gel 9	Run EcoRV	Gel 19	Run XhoI			Gel 35	*P. meandrina*	*P. meandrina*
449	Experiment	Gel 9	Run EcoRV	Gel 19	Run XhoI			Gel 35	*P. meandrina*	*P. meandrina*
450	Experiment	Gel 43	Run EcoRV	Gel 19	Run XhoI			Gel 35	*P. grandis*	*P. grandis*
451	Experiment	Gel 9	Run EcoRV	Gel 19	Run XhoI			Gel 35	*P. meandrina*	*P. meandrina*
452	Experiment	Gel 9	Run AciI-BseYI			Gel 23	*P. tuahiniensis*			*P. tuahiniensis*
454	Experiment	Gel 9	Run EcoRV	Gel 19	Run XhoI			Gel 35	*P. meandrina*	*P. meandrina*
456	Experiment	Gel 9	Run EcoRV	Gel 19	Run XhoI			Gel 35	*P. meandrina*	*P. meandrina*
457	Experiment	Gel 9	Run EcoRV	Gel 19	Run XhoI			Gel 35	*P. meandrina*	*P. meandrina*
458	Experiment	Gel 43	Run EcoRV	Gel 19	Run XhoI			Gel 35	*P. meandrina*	*P. meandrina*
459	Experiment	Gel 9	Run EcoRV	Gel 19	Run XhoI			Gel 35	*P. meandrina*	*P. meandrina*
460	Experiment	Gel 9	Run EcoRV	Gel 19	Run XhoI			Gel 35	*P. grandis*	*P. grandis*

462	Experiment	Gel 9	Run AciI-BseYI			Gel 23	*P. tuahiniensis*			*P. tuahiniensis*
463	Experiment	Gel 43	Run EcoRV	Gel 19	Run XhoI			Gel 35	*P. meandrina*	*P. meandrina*
464	Experiment	Gel 9	Run EcoRV	Gel 19	Run XhoI			Gel 35	*P. meandrina*	*P. meandrina*
465	Experiment	Gel 9	Run AciI-BseYI			Gel 23	*P. tuahiniensis*			*P. tuahiniensis*
466	Experiment	Gel 9	Run EcoRV	Gel 19	Run XhoI			Gel 35	*P. meandrina*	*P. meandrina*
467	Experiment	Gel 43	Run EcoRV	Gel 19	Run XhoI			Gel 35	*P. meandrina*	*P. meandrina*
469	Experiment	Gel 43	Run EcoRV	Gel 19	Run XhoI			Gel 35	*P. meandrina*	*P. meandrina*
470	Experiment	Gel 9	Run EcoRV	Gel 19	Run XhoI			Gel 35	*P. grandis*	*P. grandis*
471	Experiment									No ID
472	Experiment	Gel 43	Run EcoRV	Gel 19	*P. effusa*					*P. effusa*
473	Experiment	Gel 43	*P. acuta*							*P. acuta*
474	Experiment	Gel 10	Run EcoRV	Gel 21	Run XhoI			Gel 35	*P. grandis*	*P. grandis*
475	Experiment			Gel 21	Run XhoI			Gel 35		No ID
478	Experiment	Gel 10	Run EcoRV	Gel 21	Run XhoI			Gel 35	*P. grandis*	*P. grandis*
479	Experiment	Gel 10	Run EcoRV	Gel 21	Run XhoI			Gel 35	*P. meandrina*	*P. meandrina*
480	Experiment	Gel 10	Run EcoRV	Gel 21	Run XhoI			Gel 35	*P. grandis*	*P. grandis*
481	Experiment	Gel 10	Run EcoRV	Gel 21	Run XhoI			Gel 35	*P. grandis*	*P. grandis*
482	Experiment	Gel 10	Run EcoRV	Gel 21	Run XhoI			Gel 35	*P. meandrina*	*P. meandrina*
484	Experiment	Gel 10	Run EcoRV	Gel 21	Run XhoI			Gel 35	*P. grandis*	*P. grandis*
485	Experiment	Gel 10	Run AciI-BseYI			Gel 23	*P. tuahiniensis*			*P. tuahiniensis*
487	Experiment	Gel 10	Run EcoRV	Gel 21	Run XhoI			Gel 35	*P. meandrina*	*P. meandrina*
488	Experiment	Gel 10	Run EcoRV	Gel 21	Run XhoI			Gel 35	*P. meandrina*	*P. meandrina*
489	Experiment	Gel 10	Run EcoRV	Gel 21	Run XhoI			Gel 35	*P. meandrina*	*P. meandrina*
490	Experiment	Gel 10	Run EcoRV	Gel 21	Run XhoI			Gel 35	*P. meandrina*	*P. meandrina*
491	Experiment							Gel 36		No ID
492	Experiment	Gel 10	Run EcoRV	Gel 21	Run XhoI			Gel 36	*P. meandrina*	*P. meandrina*
493	Experiment	Gel 10	Run EcoRV	Gel 21	Run XhoI			Gel 36	*P. meandrina*	*P. meandrina*
494	Experiment	Gel 10	Run EcoRV	Gel 21	Run XhoI			Gel 36	*P. grandis*	*P. grandis*
495	Experiment							Gel 36		No ID
496	Experiment	Gel 10	Run EcoRV	Gel 21	*P. effusa*					*P. effusa*
497	Experiment	Gel 10	Run EcoRV	Gel 21	Run XhoI			Gel 36	*P. meandrina*	*P. meandrina*
498	Experiment	Gel 10	Run EcoRV	Gel 21	Run XhoI			Gel 36	*P. meandrina*	*P. meandrina*
499	Experiment	Gel 10	Run EcoRV	Gel 21	Run XhoI			Gel 36	*P. meandrina*	*P. meandrina*
11	Biomass	Gel 11	Run EcoRV	Gel 21	Run XhoI			Gel 36	*P. meandrina*	*P. meandrina*
13	Biomass	Gel 11	Run AciI-BseYI			Gel 25	*P. tuahiniensis*			*P. tuahiniensis*
57	Biomass	Gel 11	Run EcoRV	Gel 21	Run XhoI			Gel 36	*P. meandrina*	*P. meandrina*
61	Biomass	Gel 11	Run EcoRV	Gel 21	Run XhoI			Gel 36	*P. meandrina*	*P. meandrina*
71	Biomass	Gel 11	Run EcoRV	Gel 21	Run XhoI			Gel 36	*P. meandrina*	*P. meandrina*
83	Biomass	Gel 11	Run EcoRV	Gel 21	Run XhoI			Gel 36	*P. meandrina*	*P. meandrina*
93	Biomass	Gel 11	Run EcoRV	Gel 21	Run XhoI			Gel 36	*P. meandrina*	*P. meandrina*
113	Biomass	Gel 11	Run EcoRV	Gel 21	Run XhoI			Gel 36	*P. meandrina*	*P. meandrina*
120	Biomass	Gel 11	Run EcoRV	Gel 21	Run XhoI			Gel 36	*P. meandrina*	*P. meandrina*
128	Biomass	Gel 20	Run EcoRV	Gel 21	Run XhoI			Gel 36	*P. meandrina*	*P. meandrina*
139	Biomass	Gel 20	Run EcoRV	Gel 21	Run XhoI			Gel 36	*P. meandrina*	*P. meandrina*
159	Biomass	Gel 20	Run EcoRV	Gel 21	Run XhoI			Gel 36	*P. grandis*	*P. grandis*
167	Biomass	Gel 20	Run EcoRV	Gel 21	Run XhoI			Gel 36	*P. meandrina*	*P. meandrina*
215	Biomass	Gel 22	Run EcoRV	Gel 21	Run XhoI			Gel 36	*P. meandrina*	*P. meandrina*
240	Biomass	Gel 22	Run EcoRV	Gel 21	Run XhoI			Gel 36	*P. meandrina*	*P. meandrina*
243	Biomass	Gel 22	Run AciI-BseYI			Gel 25	*P. tuahiniensis*			*P. tuahiniensis*
246	Biomass	Gel 22	Run EcoRV	Gel 21	Run XhoI			Gel 36	*P. meandrina*	*P. meandrina*
270	Biomass	Gel 22	Run EcoRV	Gel 21	*P. effusa*					*P. effusa*
292	Biomass	Gel 22	Run EcoRV	Gel 21	Run XhoI			Gel 36	*P. meandrina*	*P. meandrina*
312	Biomass	Gel 22	Run EcoRV	Gel 21	*P. effusa*					*P. effusa*
336	Biomass	Gel 22	Run EcoRV	Gel 21	Run XhoI			Gel 36	*P. meandrina*	*P. meandrina*
339	Biomass	Gel 22	Run EcoRV	Gel 21	Run XhoI			Gel 40	*P. meandrina*	*P. meandrina*
347	Biomass	Gel 22	Run EcoRV	Gel 21	Run XhoI			Gel 40	*P. meandrina*	*P. meandrina*
365	Biomass	Gel 22	Run EcoRV	Gel 21	Run XhoI			Gel 36	*P. grandis*	*P. grandis*
394	Biomass							Gel 40		No ID
409	Biomass	Gel 22	Run AciI-BseYI			Gel 25	*P. verrucosa*			*P. verrucosa*
410	Biomass	Gel 22	Run EcoRV	Gel 25	*P. effusa*					*P. effusa*
420	Biomass	Gel 22	Run EcoRV	Gel 25	Run XhoI			Gel 40	*P. grandis*	*P. grandis*
434	Biomass	Gel 22	Run EcoRV	Gel 25	Run XhoI					No ID
453	Biomass	Gel 22	Run EcoRV	Gel 25	Run XhoI			Gel 40	*P. meandrina*	*P. meandrina*
455	Biomass	Gel 22	Run EcoRV	Gel 25	Run XhoI			Gel 40	*P. meandrina*	*P. meandrina*
476	Biomass	Gel 22	Run EcoRV	Gel 25	Run XhoI			Gel 40	*P. meandrina*	*P. meandrina*
477	Biomass	Gel 22	Run AciI-BseYI			Gel 37	*P. verrucosa*			*P. verrucosa*
483	Biomass	Gel 22	Run EcoRV	Gel 25	Run XhoI			Gel 40	*P. meandrina*	*P. meandrina*
500	Biomass	Gel 43	Run EcoRV	Gel 25	Run XhoI			Gel 40	*P. grandis*	*P. grandis*
