# Supplementary material for: The Role of Light in Mediating Physiological Stress in Small Corals Exposed to Elevated Temperature
Source: Ecol Evol. 2026 Nov 12;16(11):e74505. doi: 10.1002/ece3.74505 (PMC13756585; doi:10.1002/ece3.74505)

SUPPLEMENTARY MATERIAL 2

The role of light in mediating physiological stress in small corals exposed to elevated temperature

README

This supplemental material contains all gel images used to identify the coral samples. Digested PCR amplicons and Quick-Load Purple 50 bp DNA Ladder were run on 2% agarose gels for 45–70 minutes at 100V and visualized using SYBR Safe DNA Gel Stain on a blue light transilluminator. Species identifications were determined based on banding patterns from all digests run for that sample, as described in Cohn et al. (2026). The size range guide for the ladder used in all gel images is included to the right, for reference. See Supplemental Materials and Methods 1 for a list of all samples, their species identification, and the gel images used to identify each of them.

Coral samples were numbered 1–500 in this dataset, and gel images are annotated with the sample number corresponding to each lane. Lanes labeled with “X” were not used for identification because: 1) they were blank, 2) the image was too faint or blurry for confident interpretation, or 3) the sample was run in duplicate and is interpreted elsewhere.

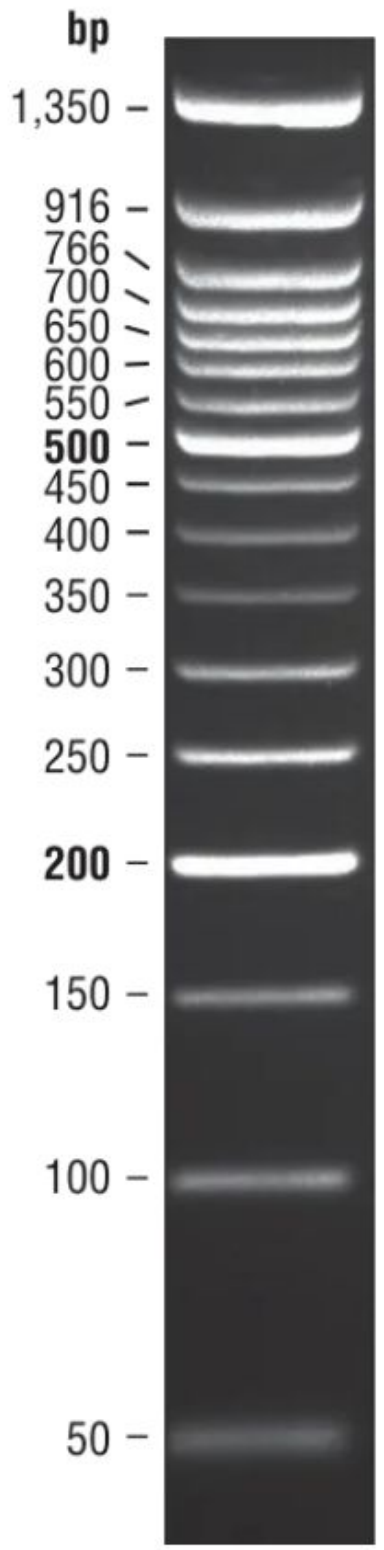

Gel 1
NlaIV
19 Feb 26

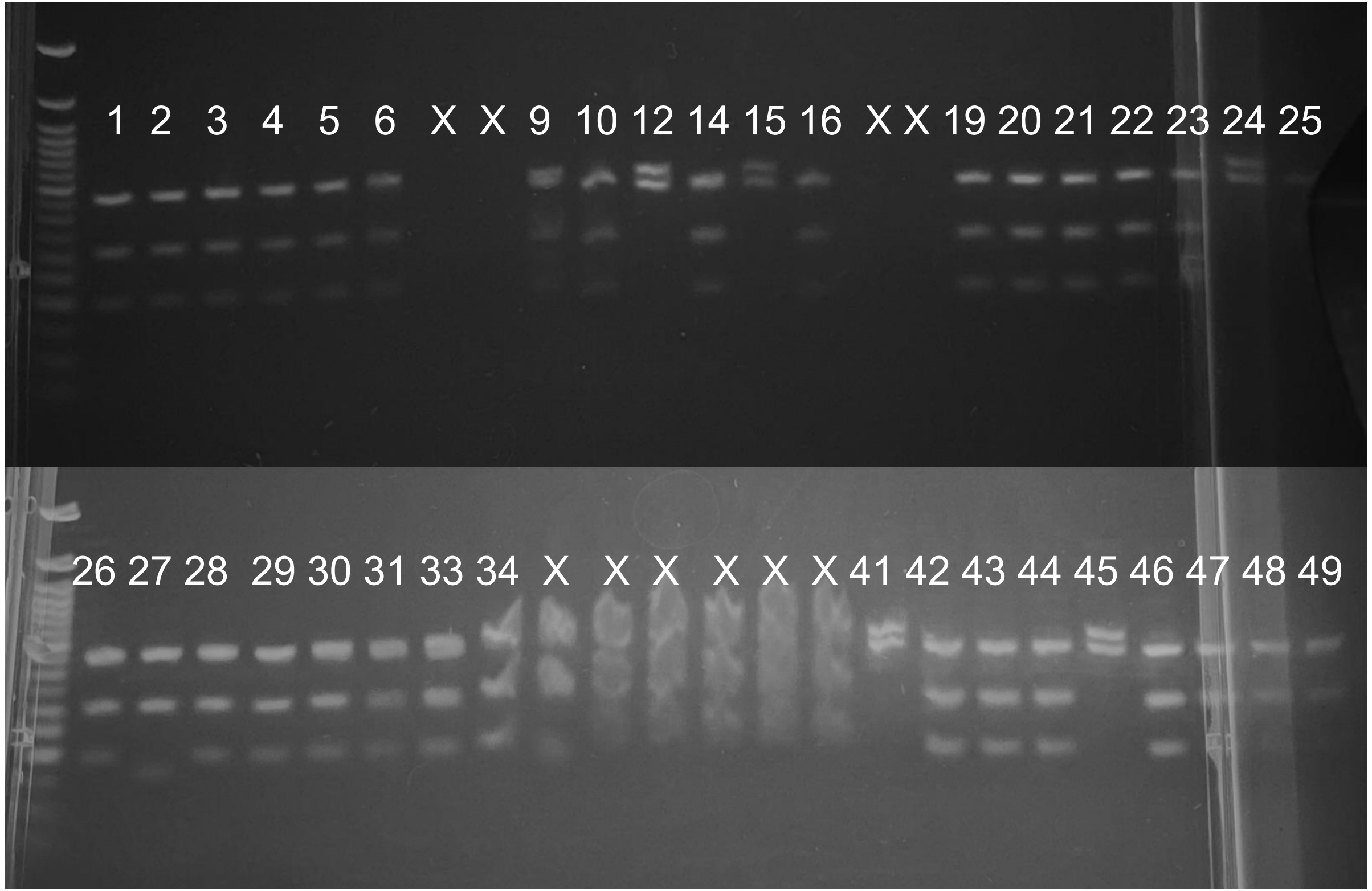

Gel 2
NlaIV
19 Feb 26

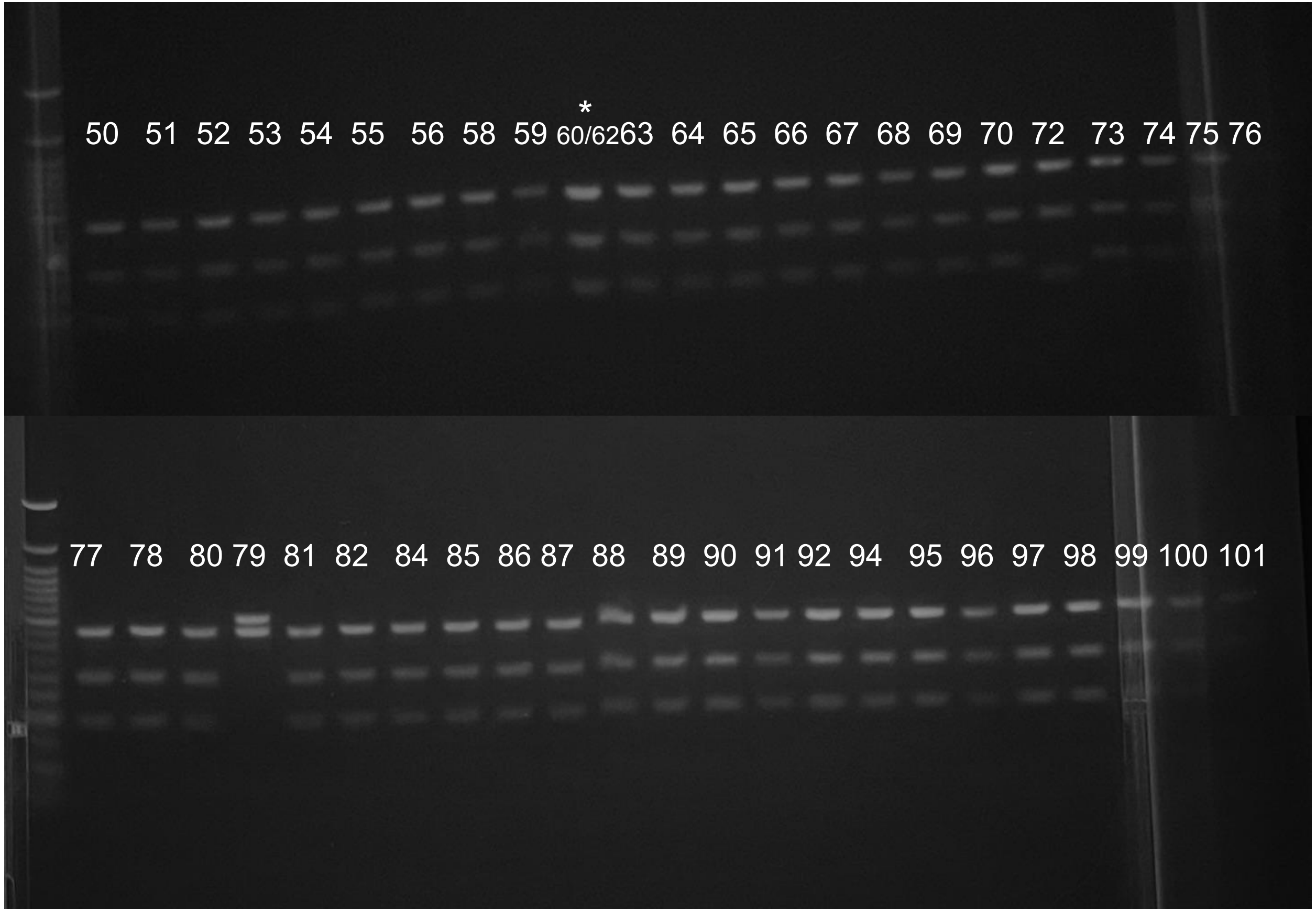

*Both Sample 60 and Sample 62 were loaded into the same well

Gel 3
NlaIV
20 Feb 26

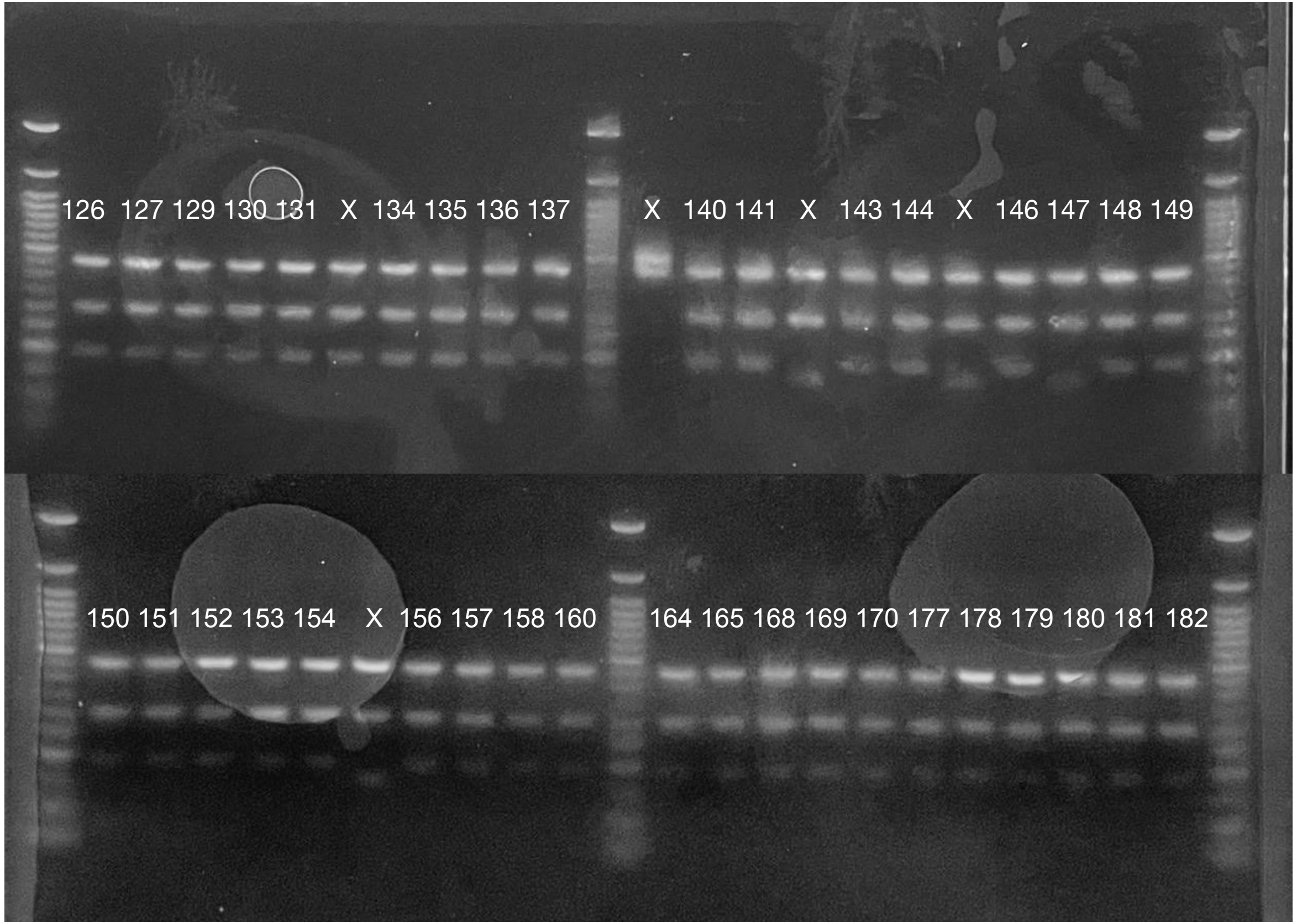

Gel 4
NlaIV
20 Feb 26

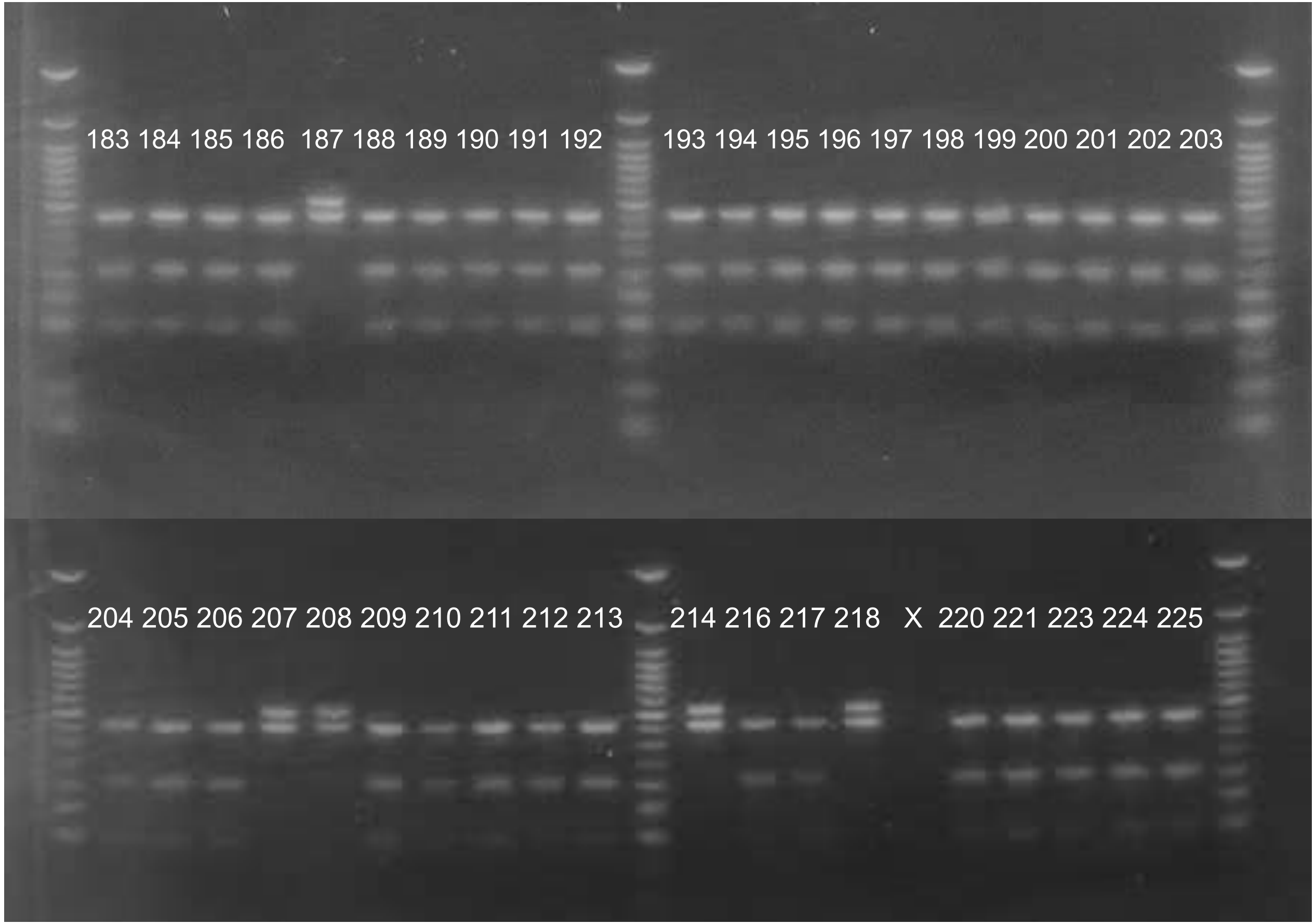

Gel 5
NlaIV
20 Feb 26

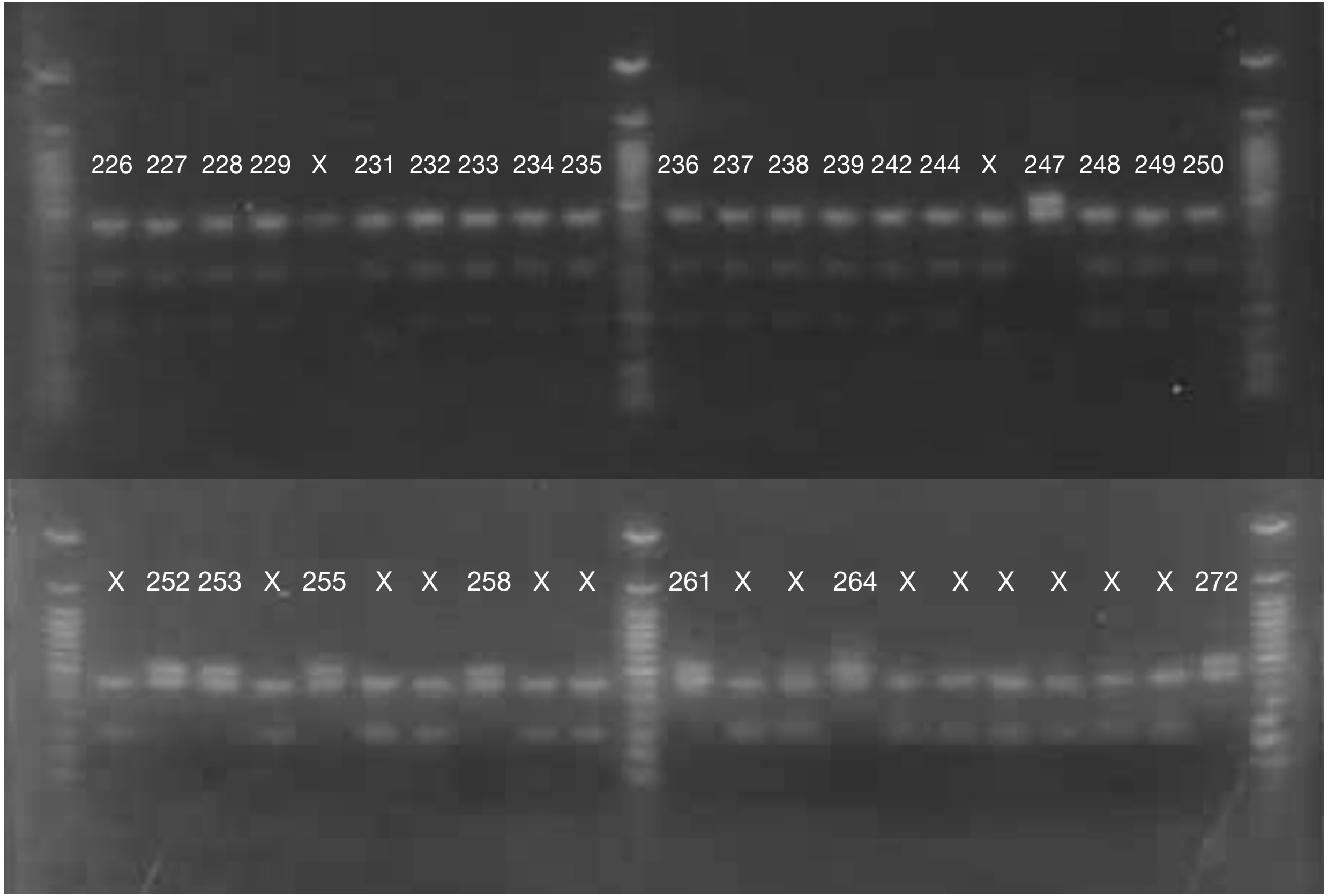

Gel 6
NlaIV
20 Feb 26

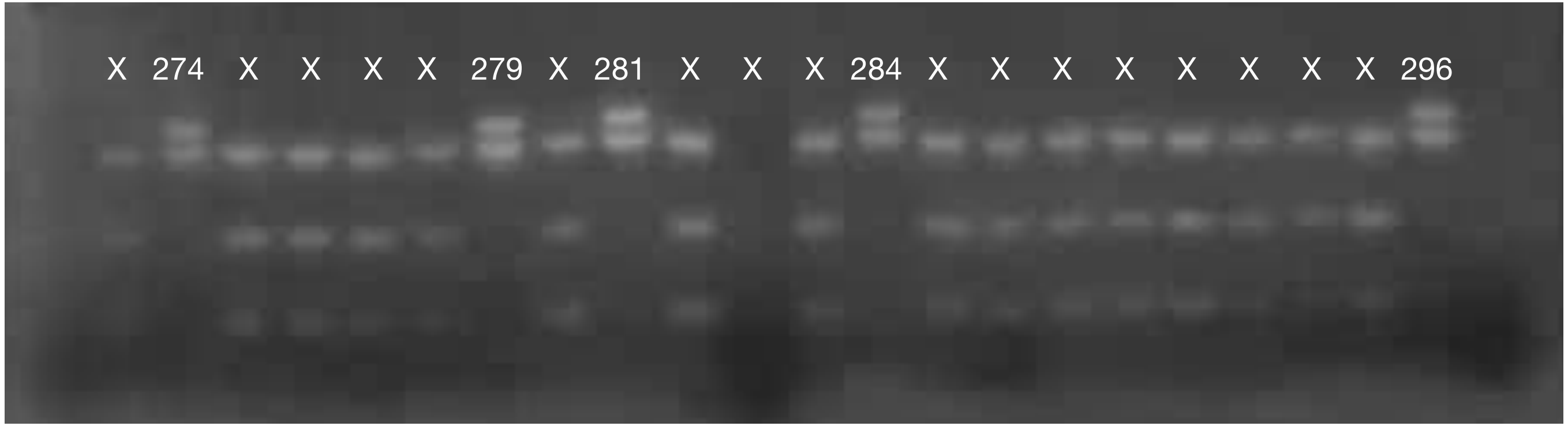

* Loading dye loaded instead of ladder

Gel 7
NlaIV
20 Feb 26

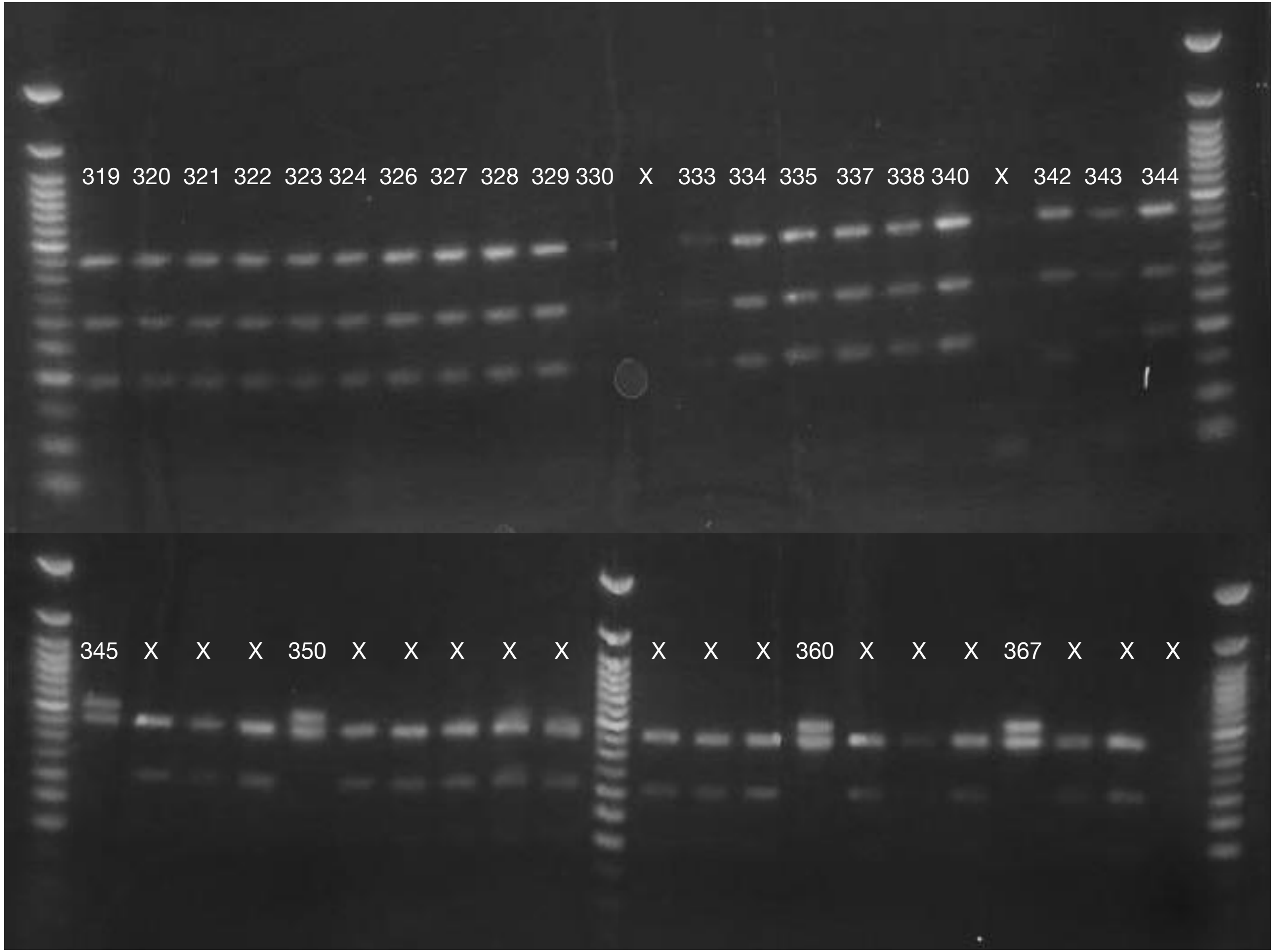

Gel 8
NlaIV
20 Feb 26

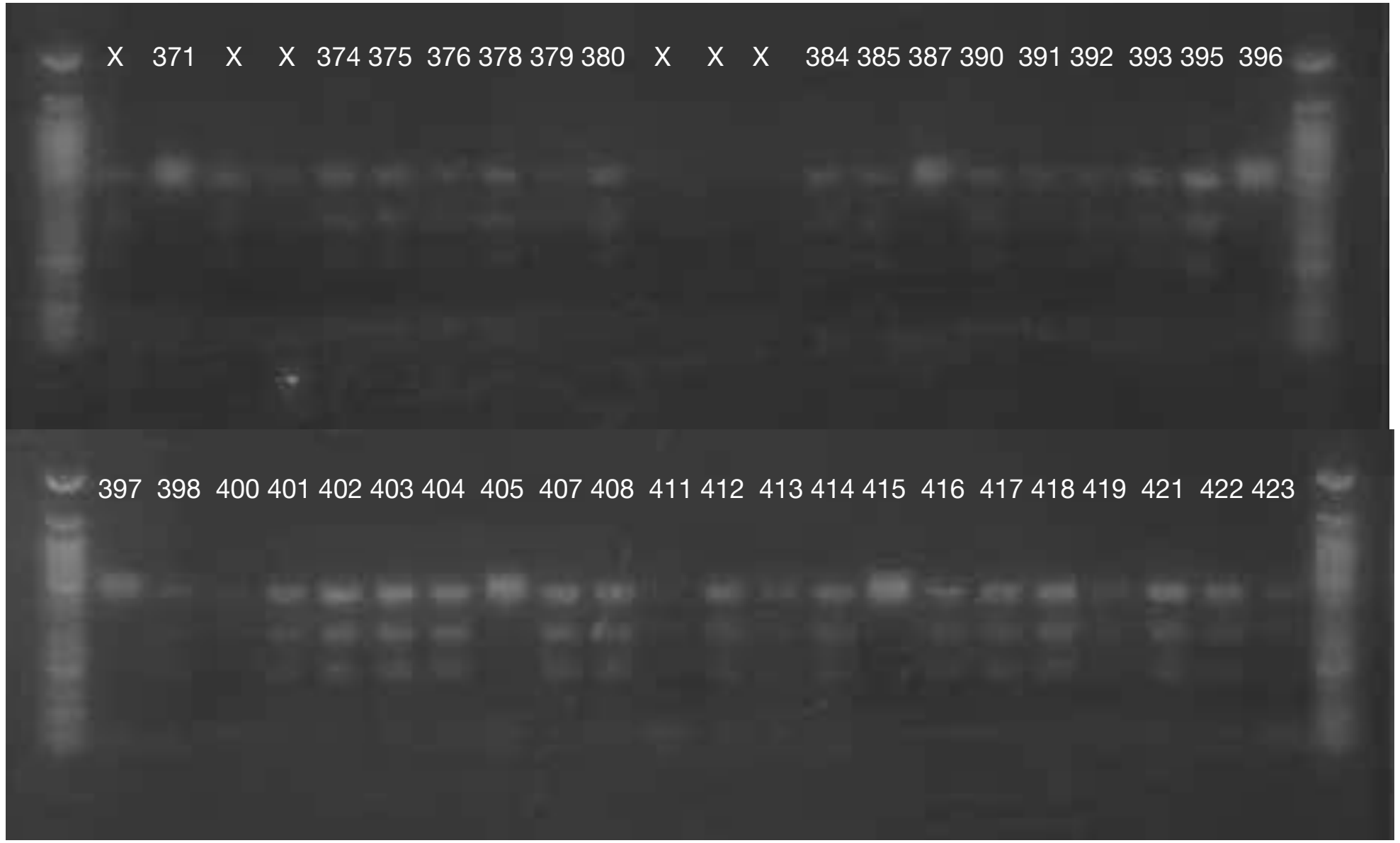

Gel 9
NlaIV
20 Feb 26

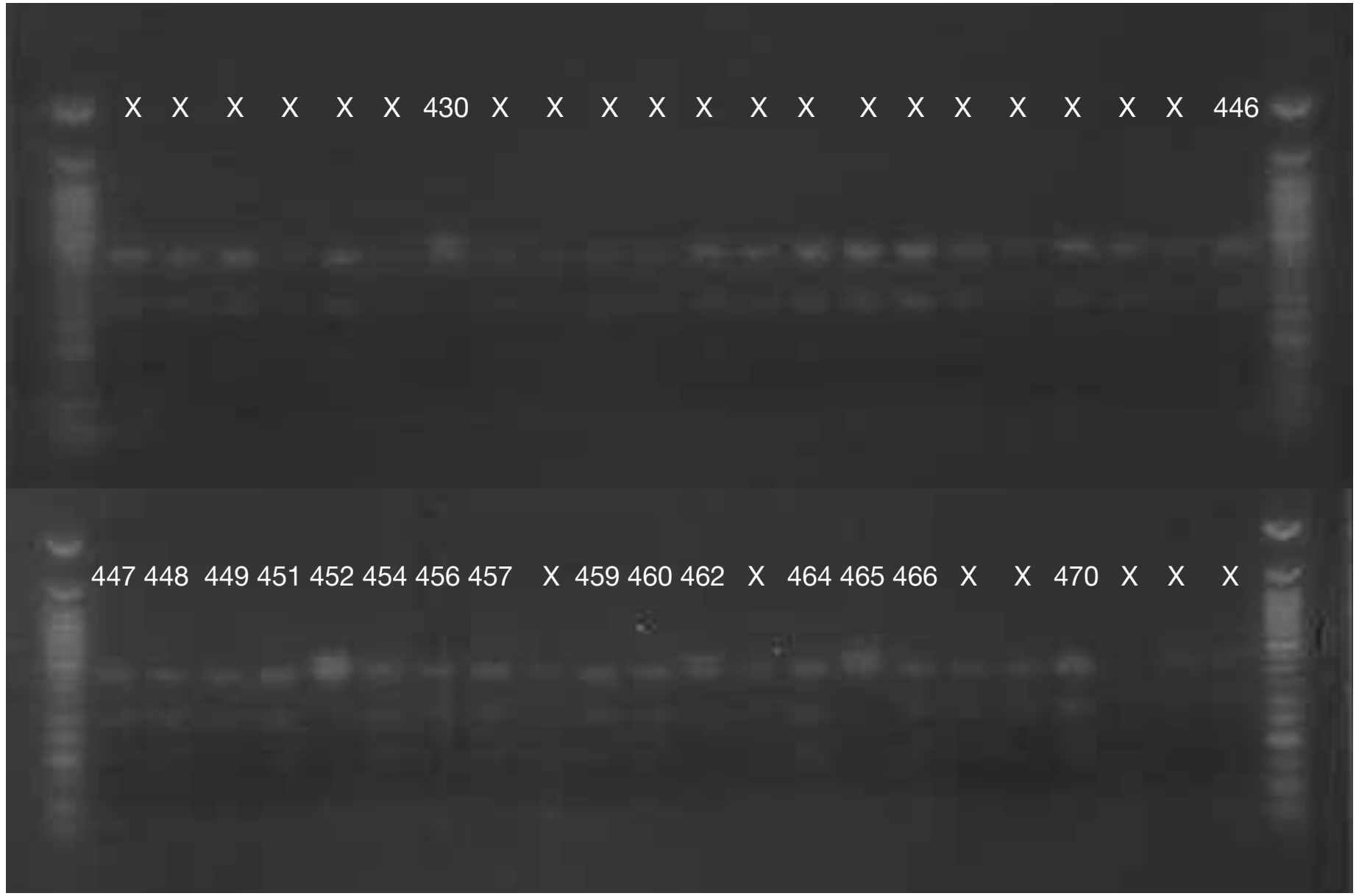

Gel 10
NlaIV
20 Feb 26

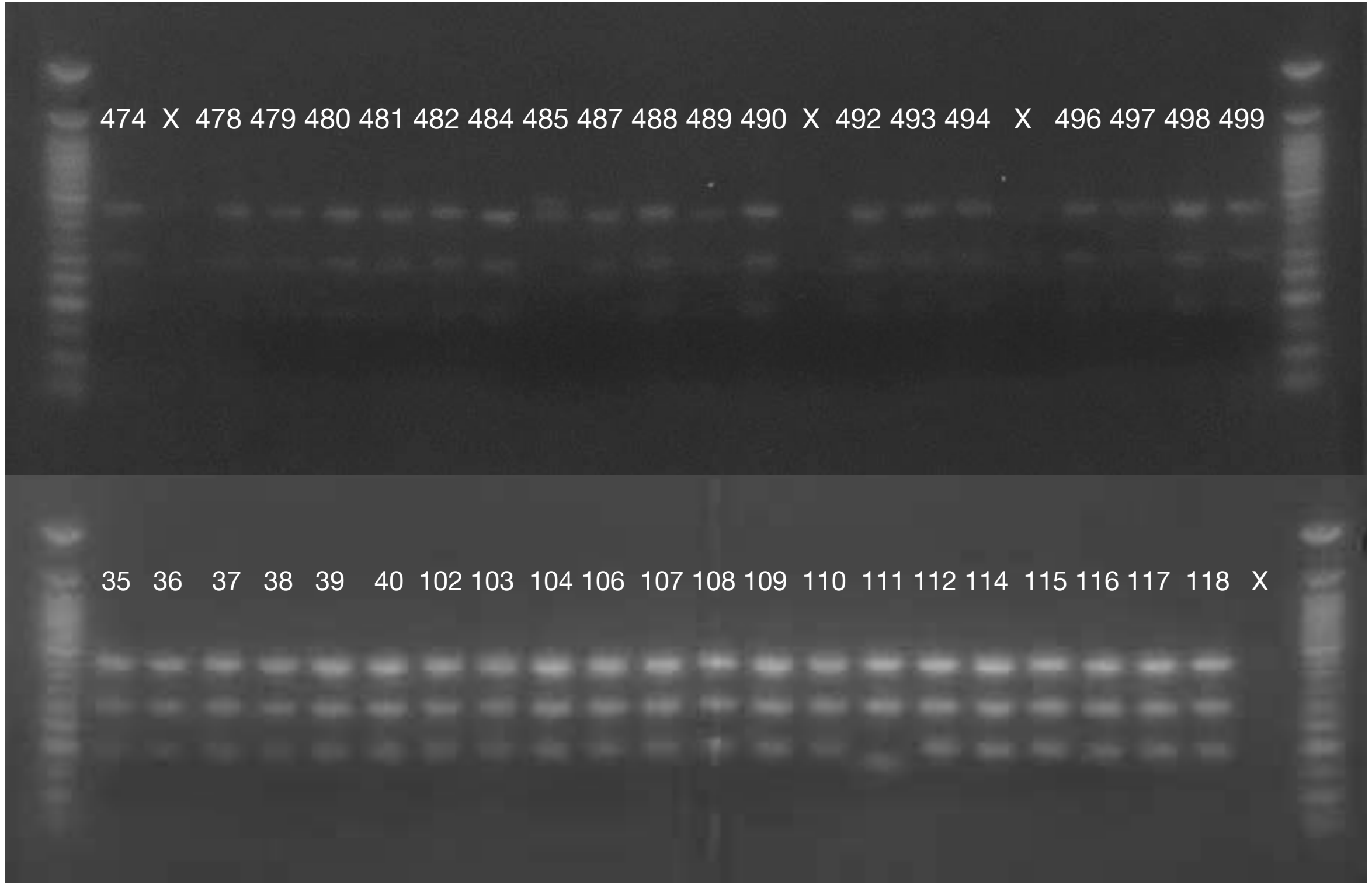

Gel 11
NlaIV
20 Feb 26

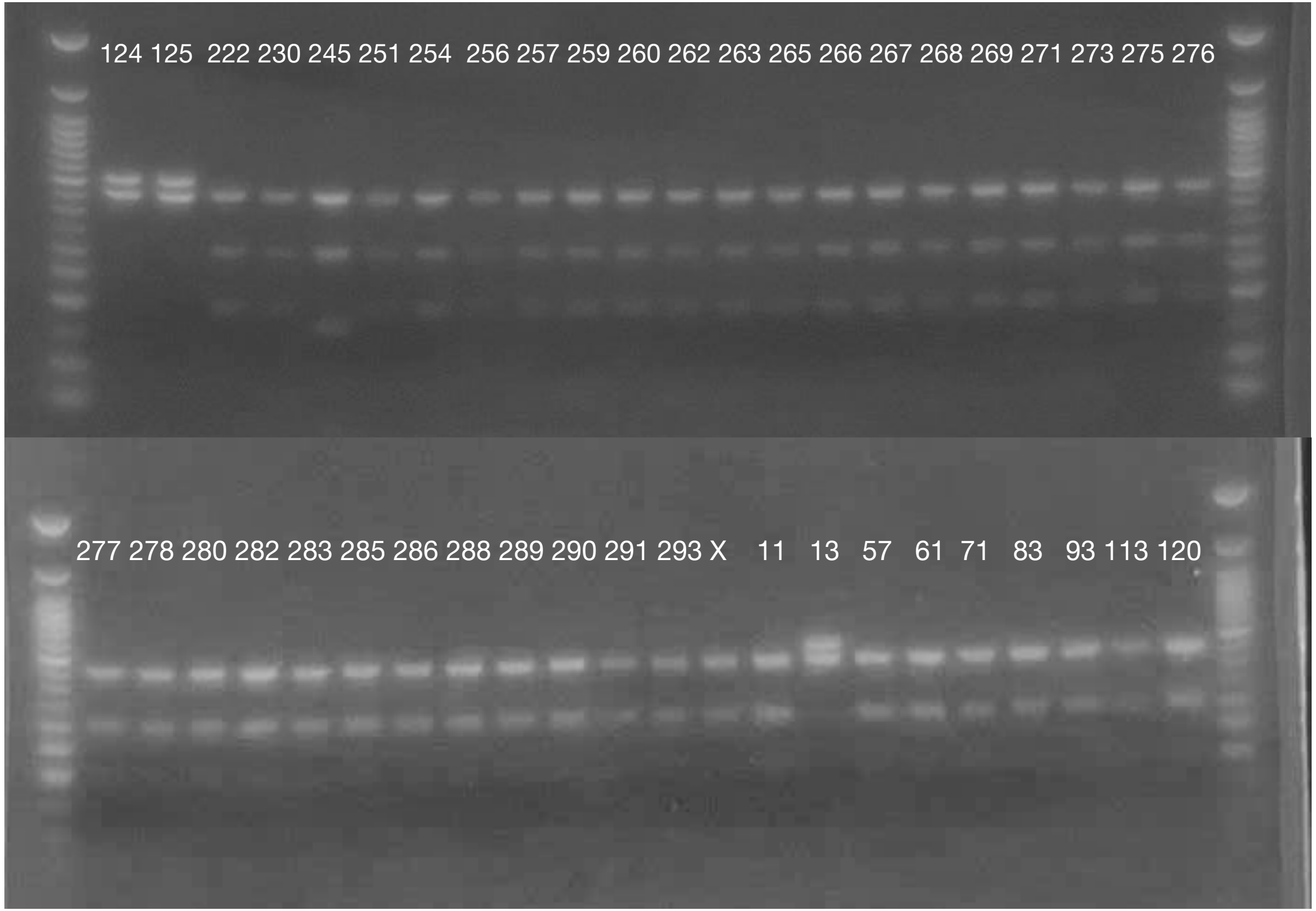

Gel 12
EcoRV-HF
21 Feb 26

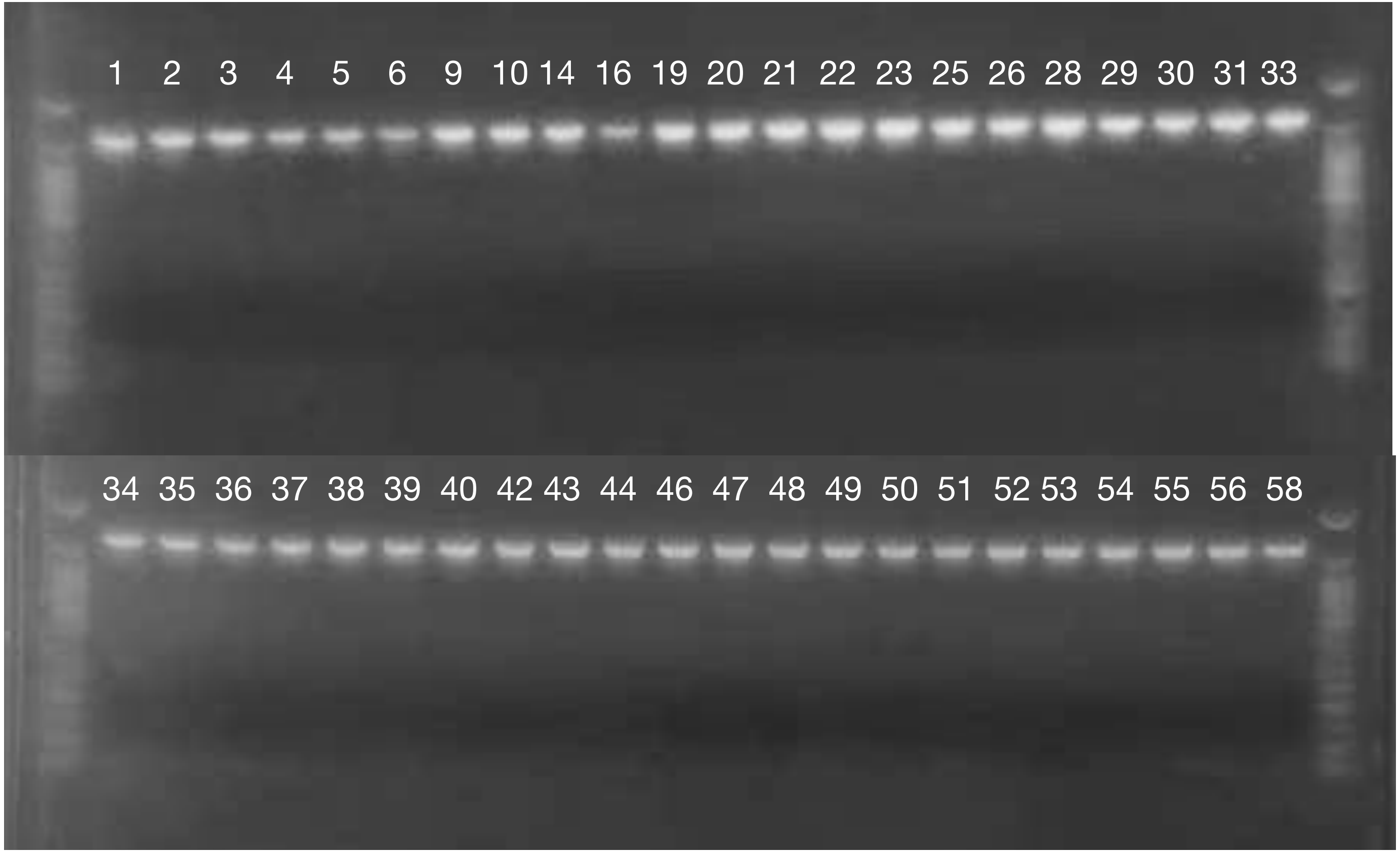

Gel 13
EcoRV-HF
21 Feb 26

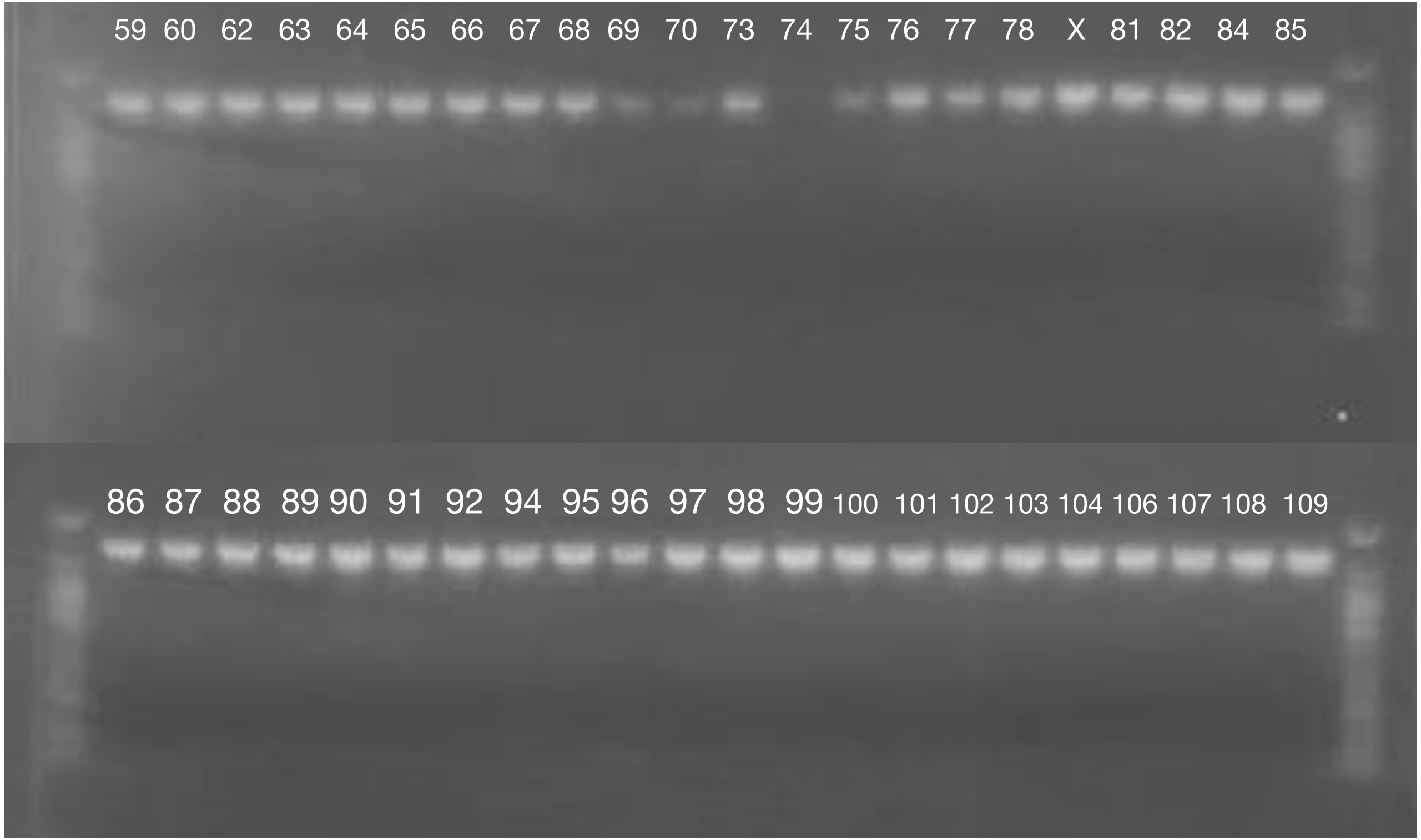

Gel 14
EcoRV-HF
21 Feb 26

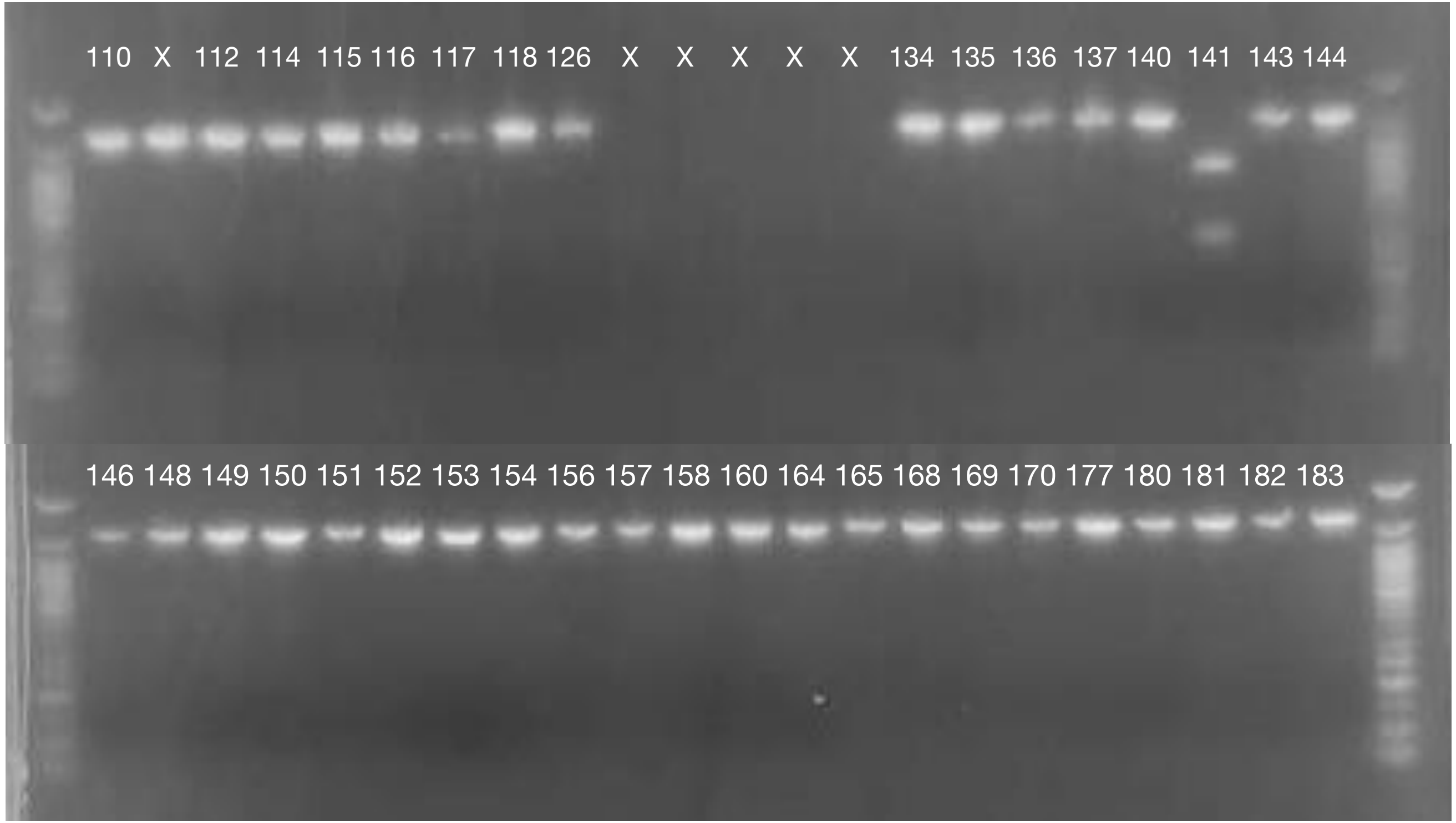

Gel 15
EcoRV-HF
21 Feb 26

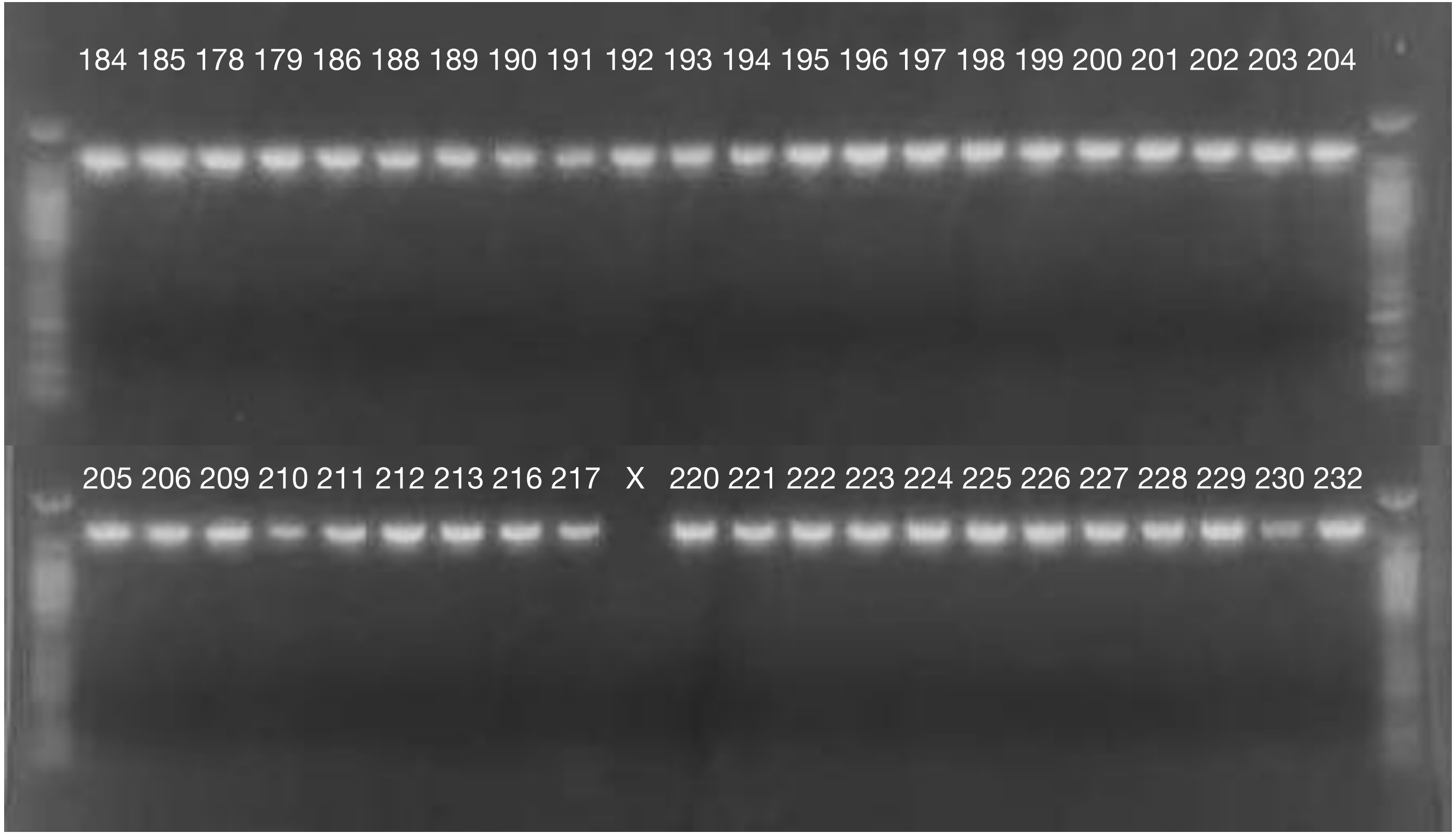

Gel 16
EcoRV-HF
21 Feb 26

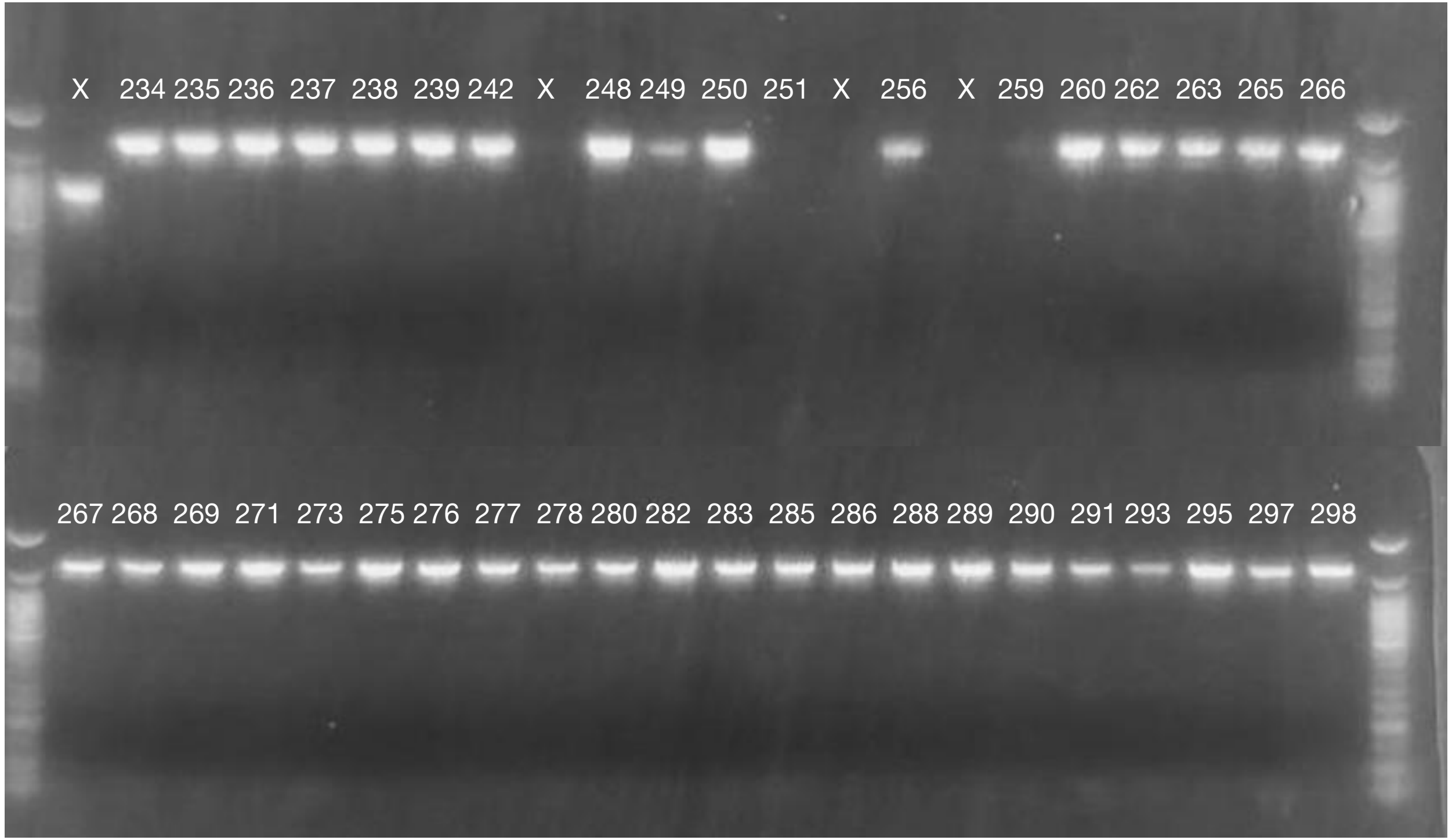

Gel 17
EcoRV-HF
21 Feb 26

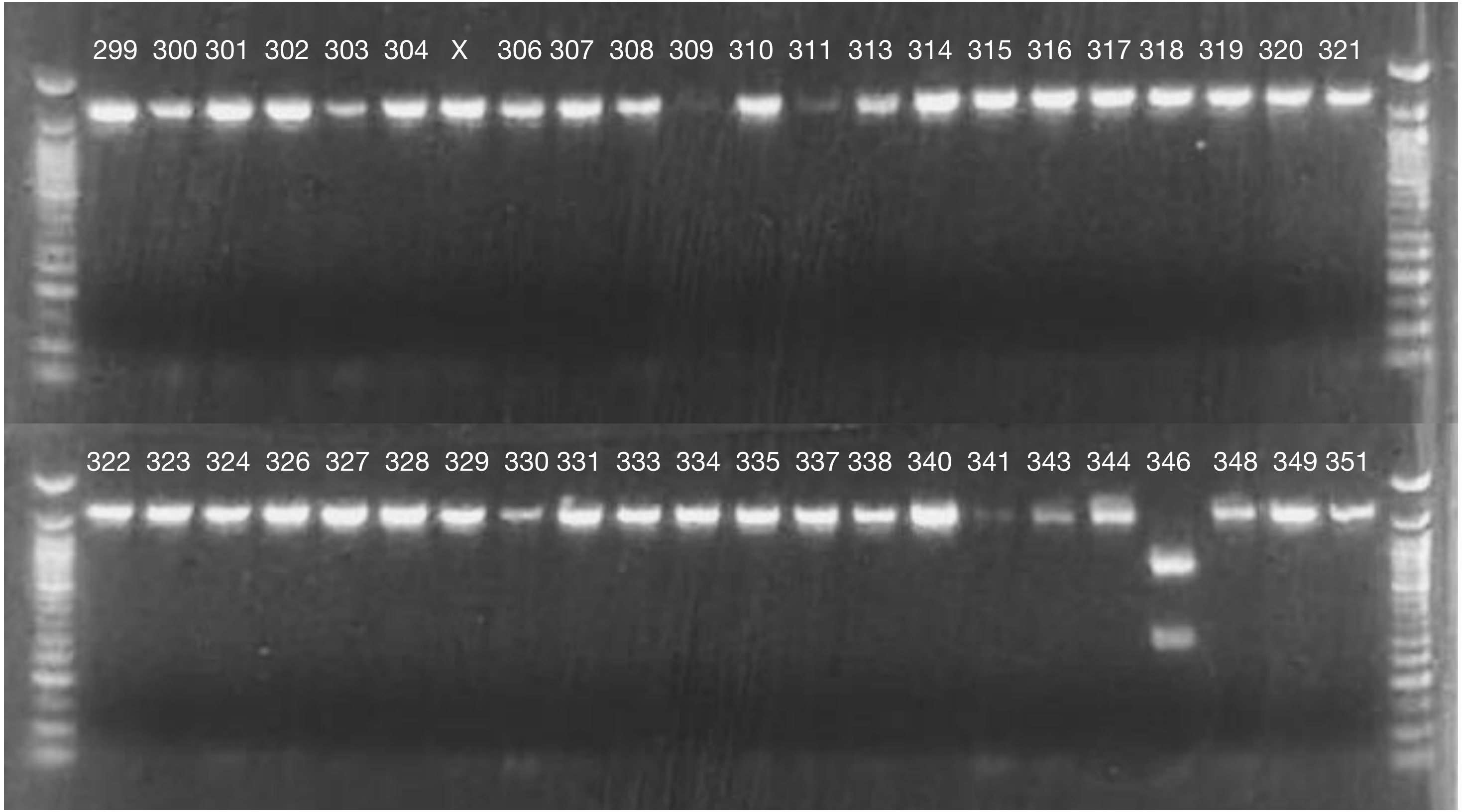

Gel 18
EcoRV-HF
18 Feb 26

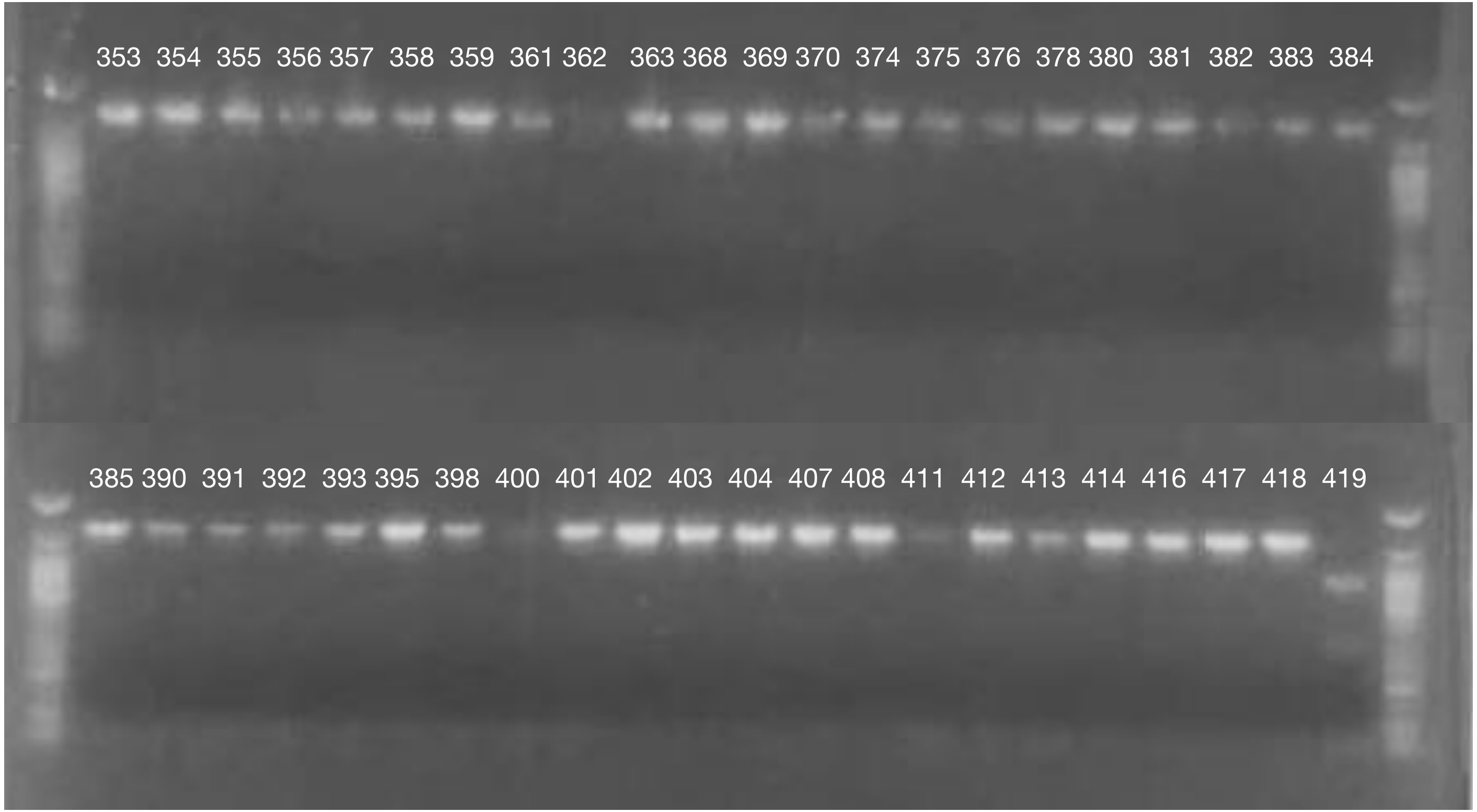

Gel 19
EcoRV-HF
21 Feb 26

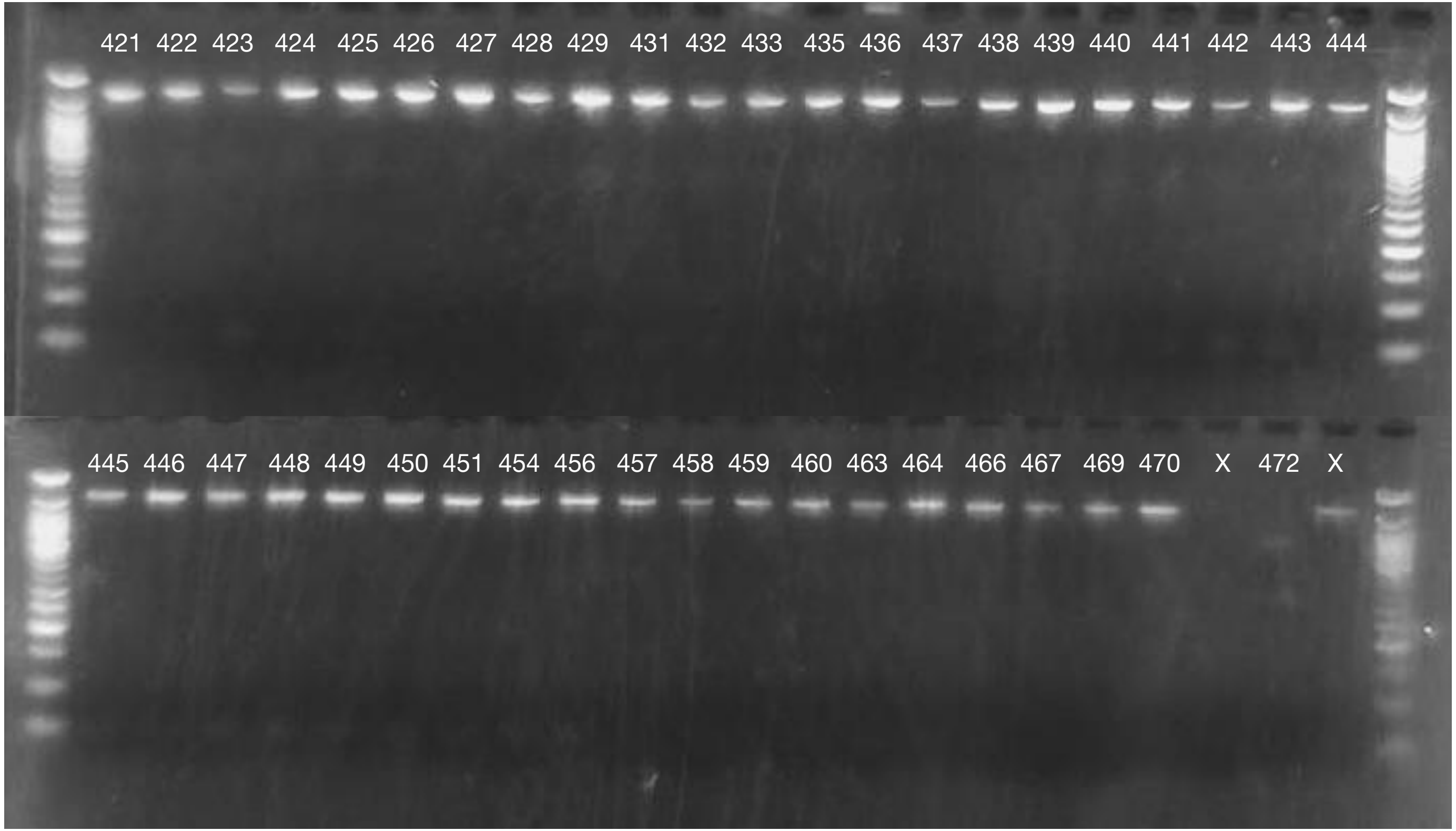

Gel 20
NlaIV
22 Feb 26

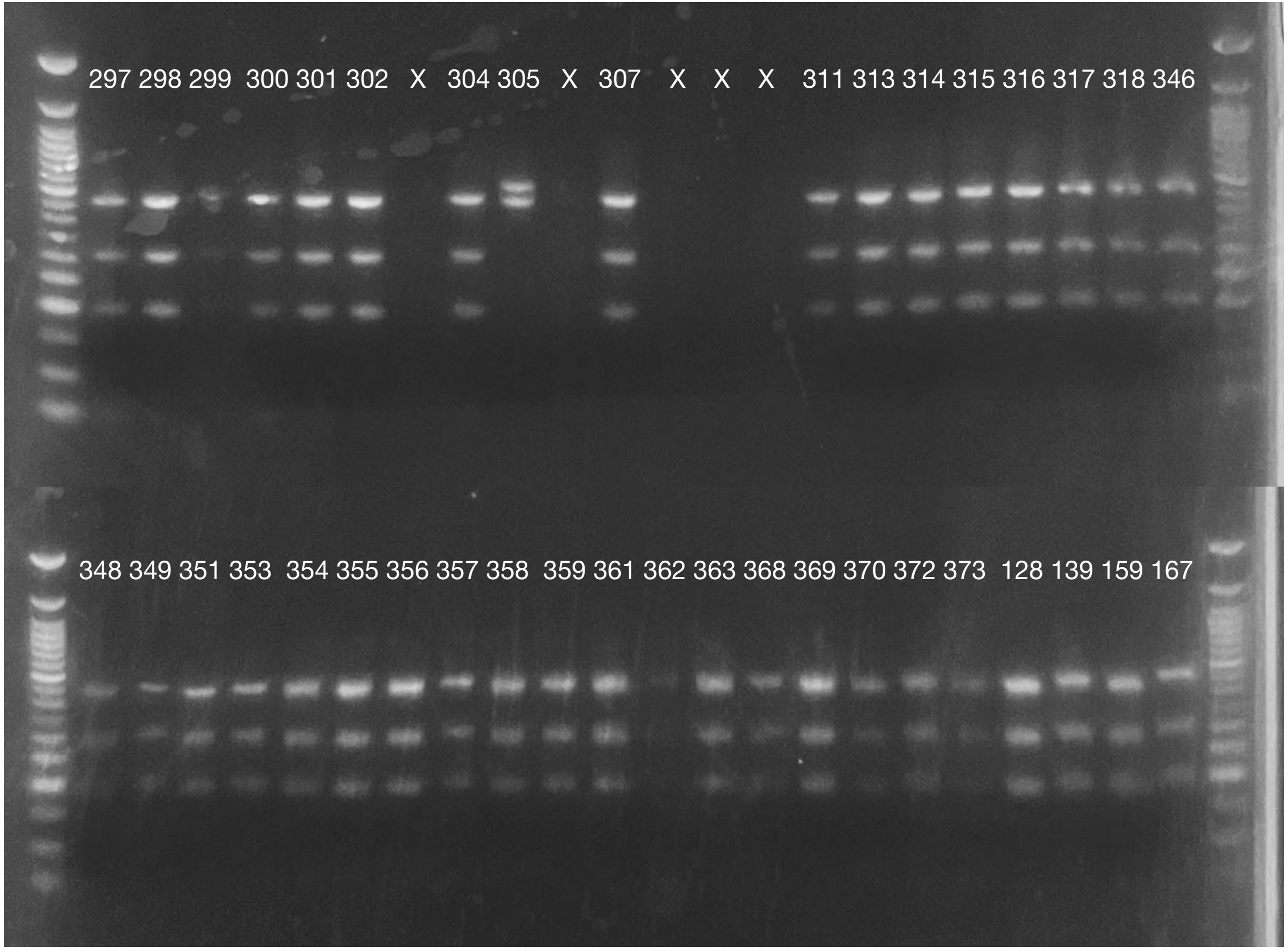

Gel 21
EcoRV-HF
22 Feb 26

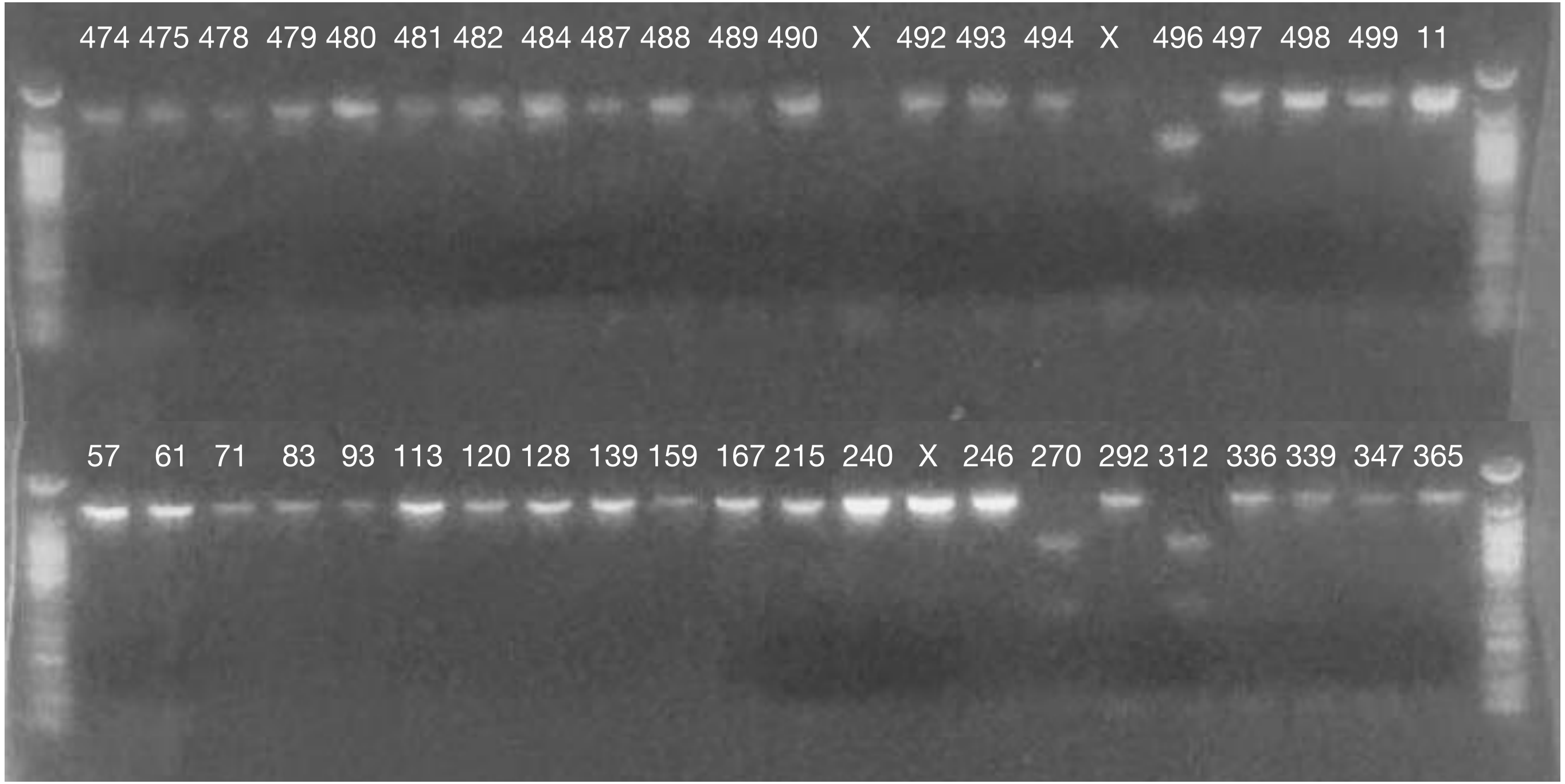

Gel 22
NlaIV
22 Feb 26

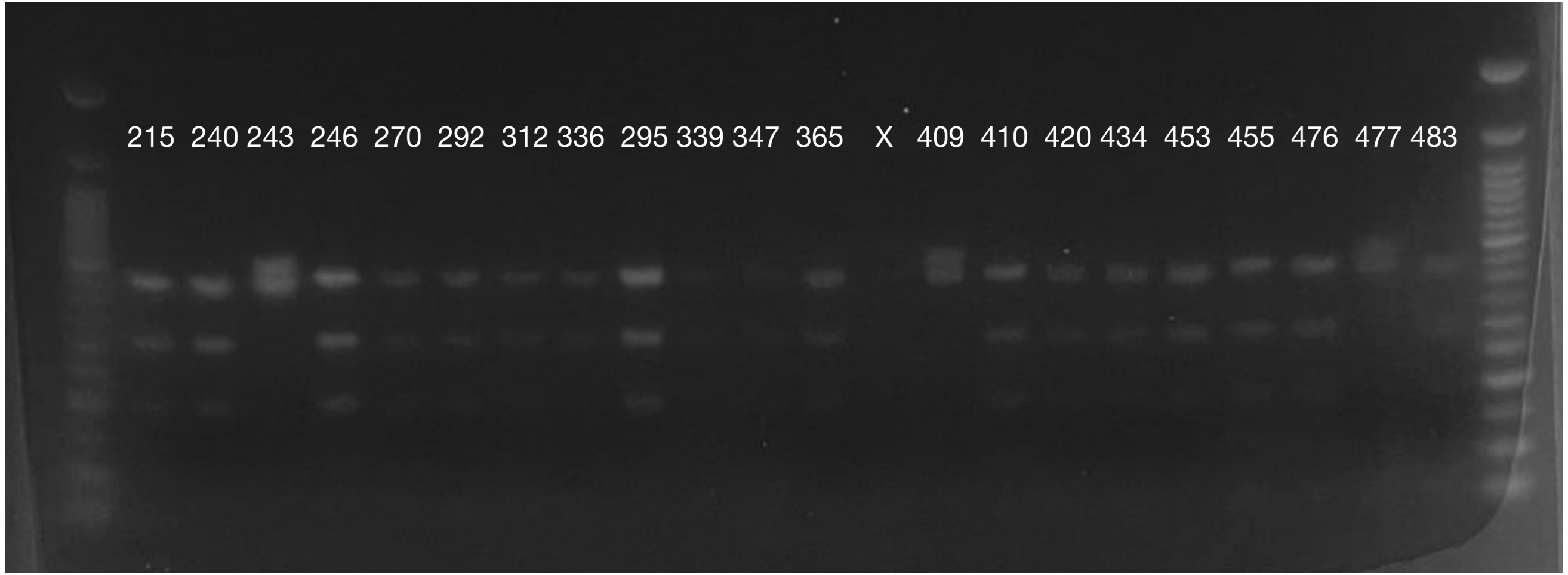

Gel 23
AciI-BseYI
22 Feb 26

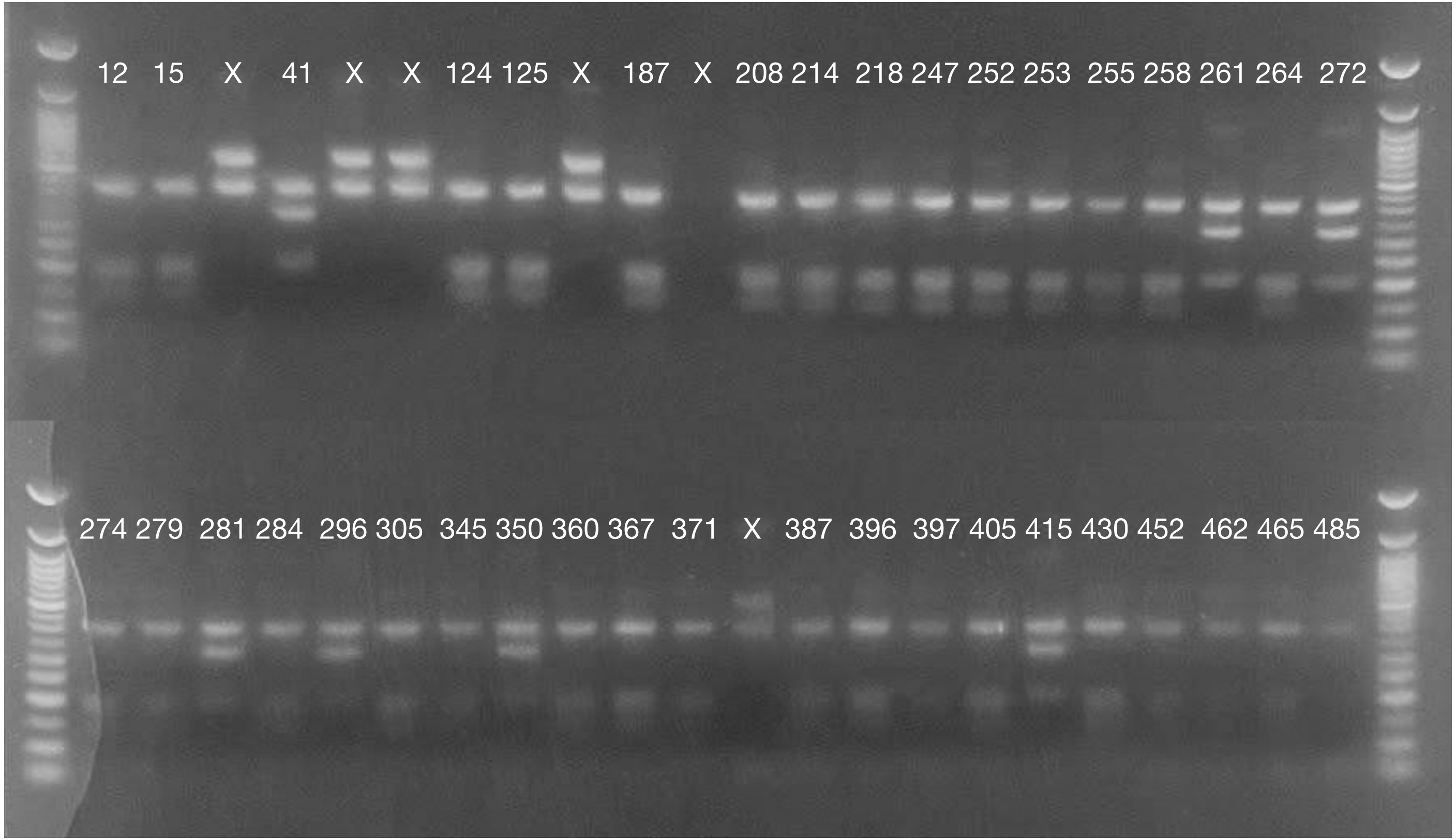

Gel 24
NlaIV
22 Feb 26

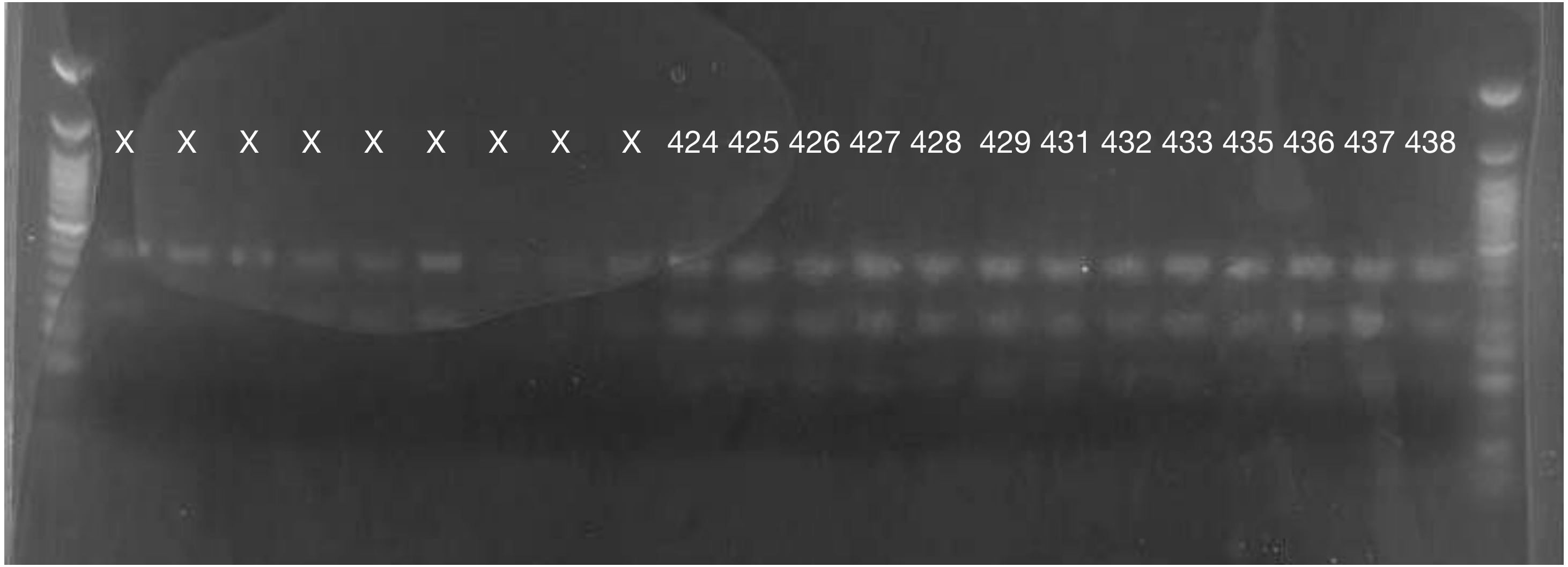

Gel 25
EcoRV-HF, AciI-BseYI
22 Feb 26

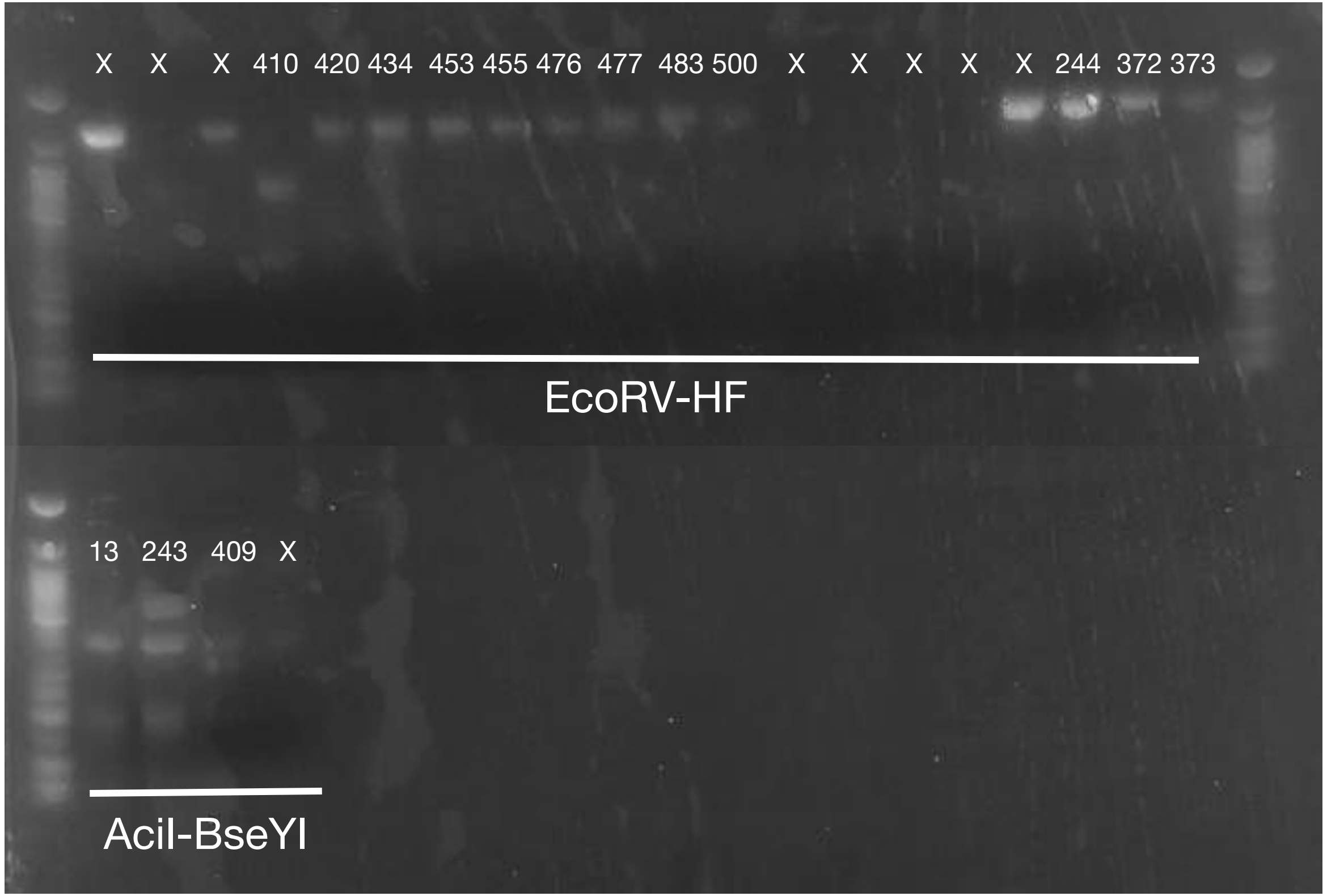

Gel 26
XhoI
23 Feb 26

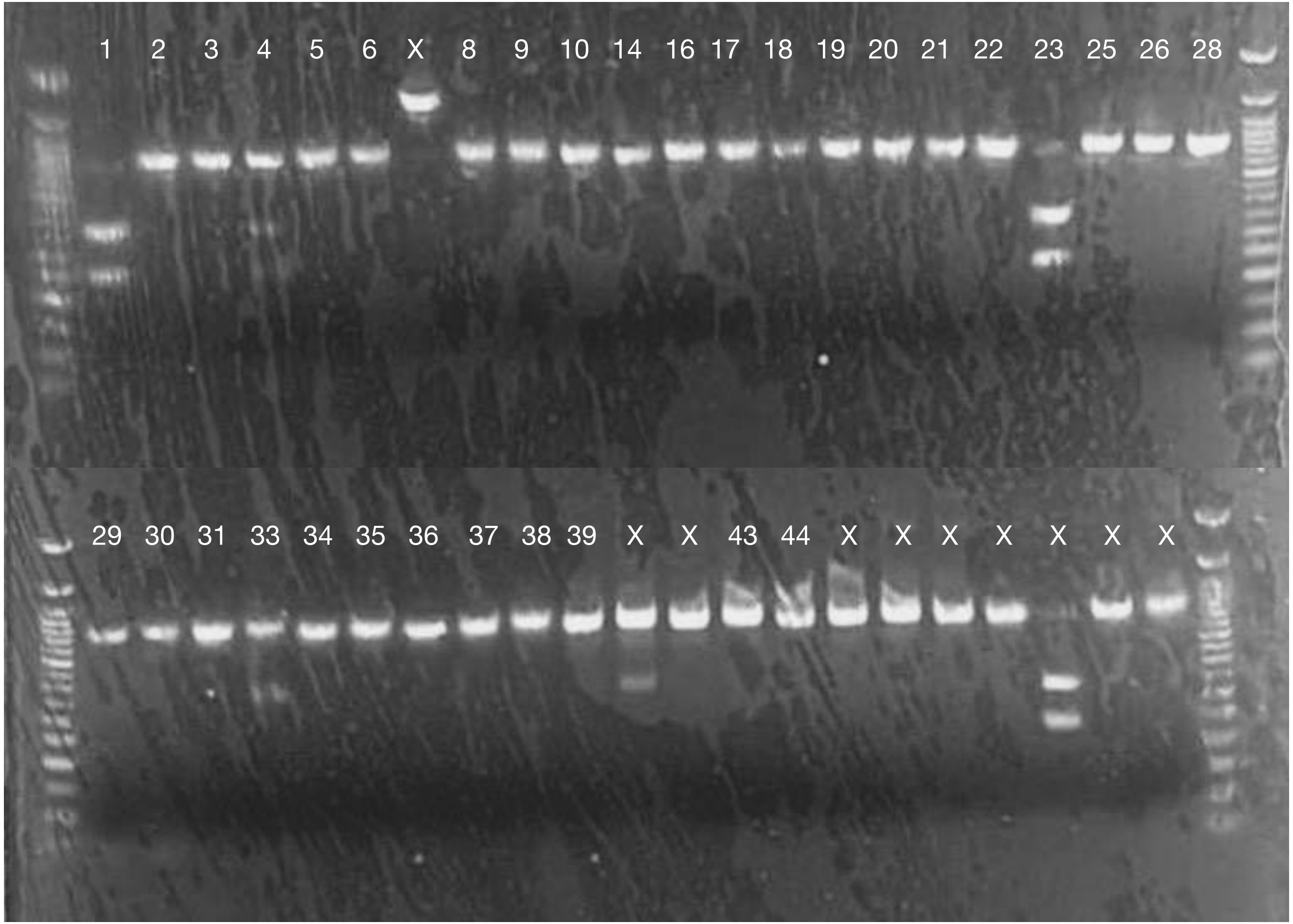

Gel 27
XhoI
23 Feb 26

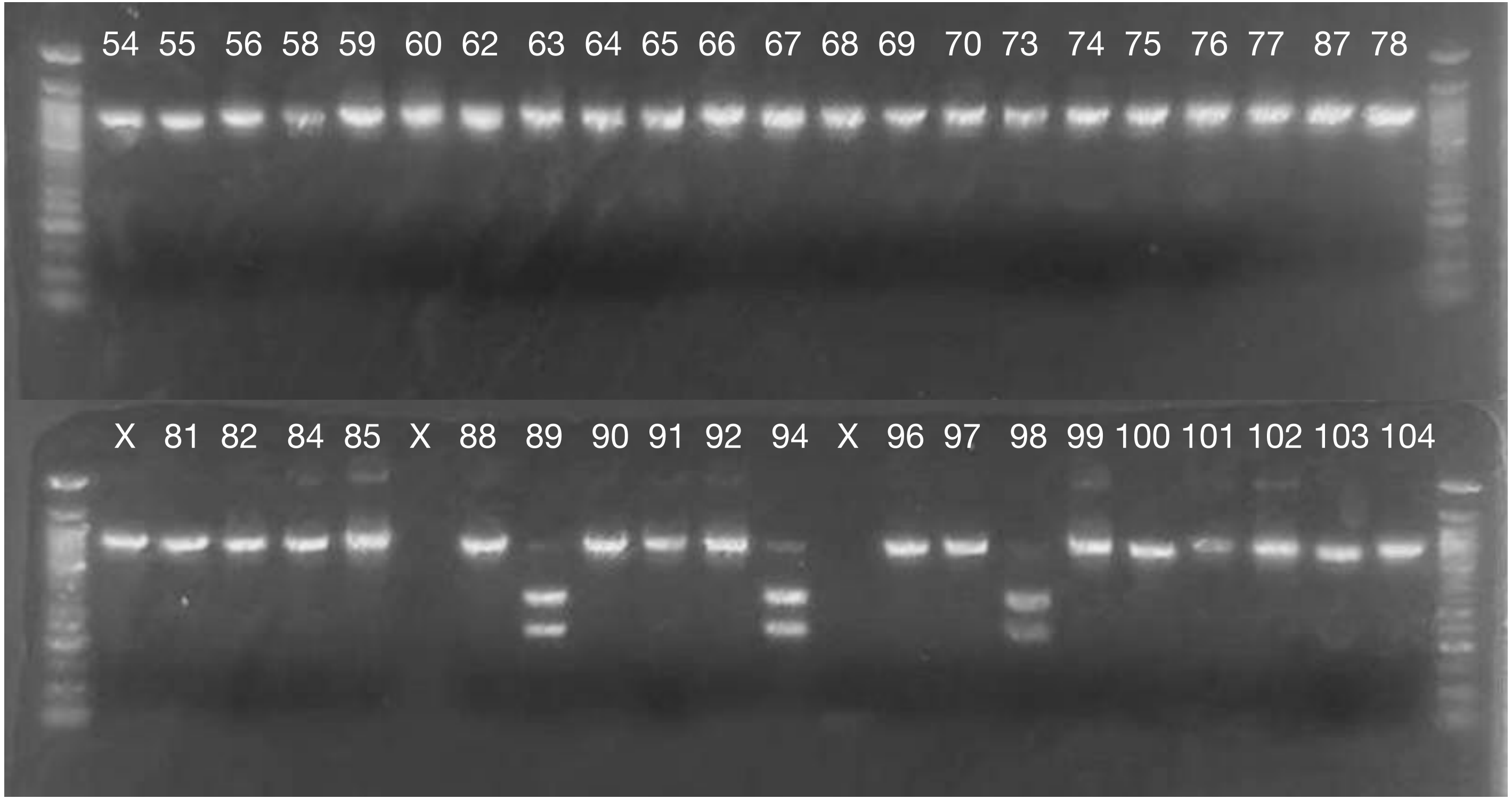

Gel 28
AciI-BseYI
23 Feb 26

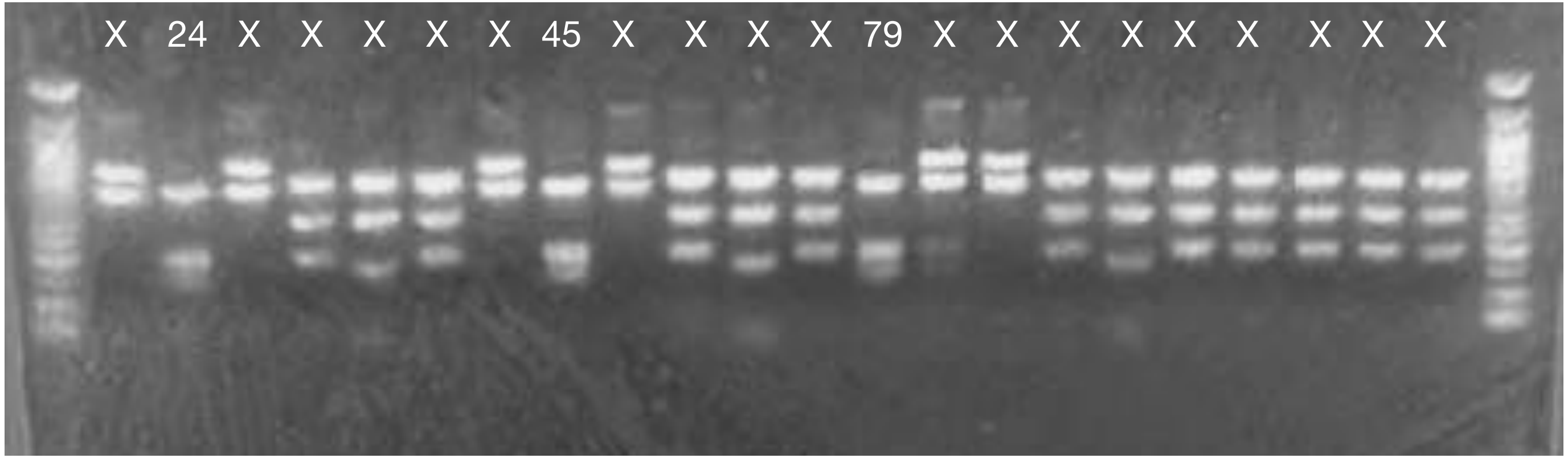

Gel 29
XhoI
23 Feb 26

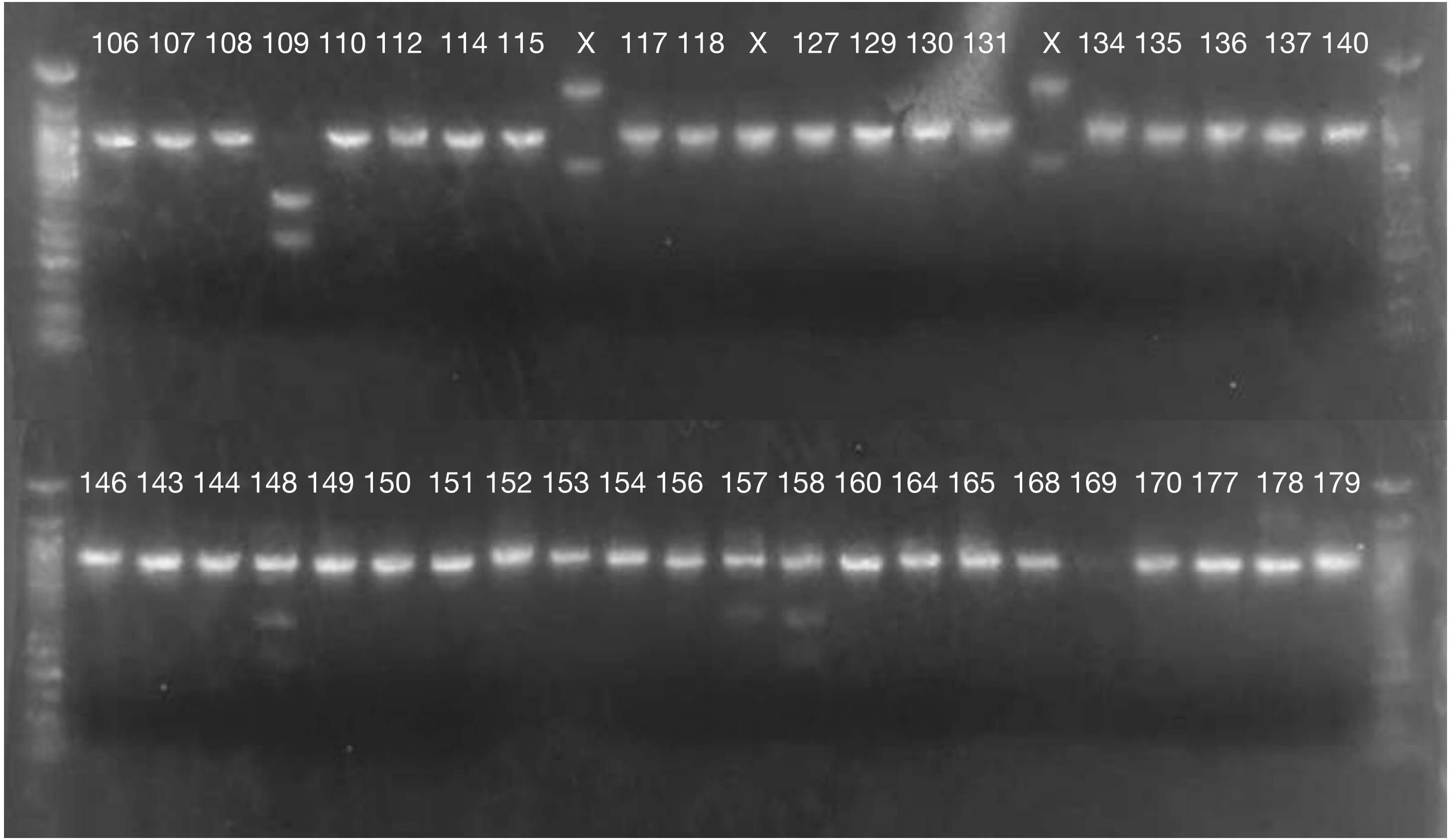

Gel 30
XhoI
23 Feb 26

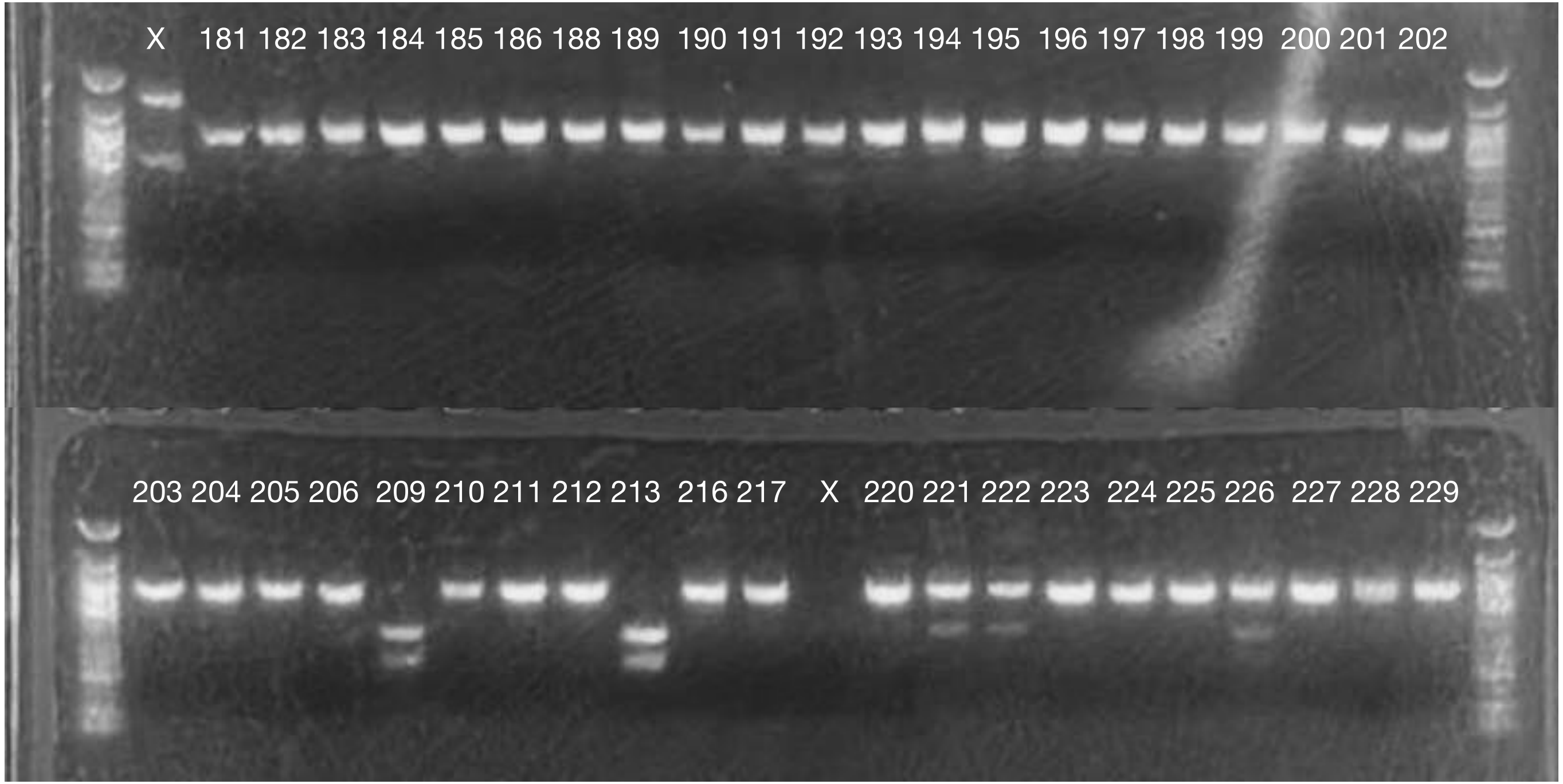

Gel 31
XhoI
23 Feb 26

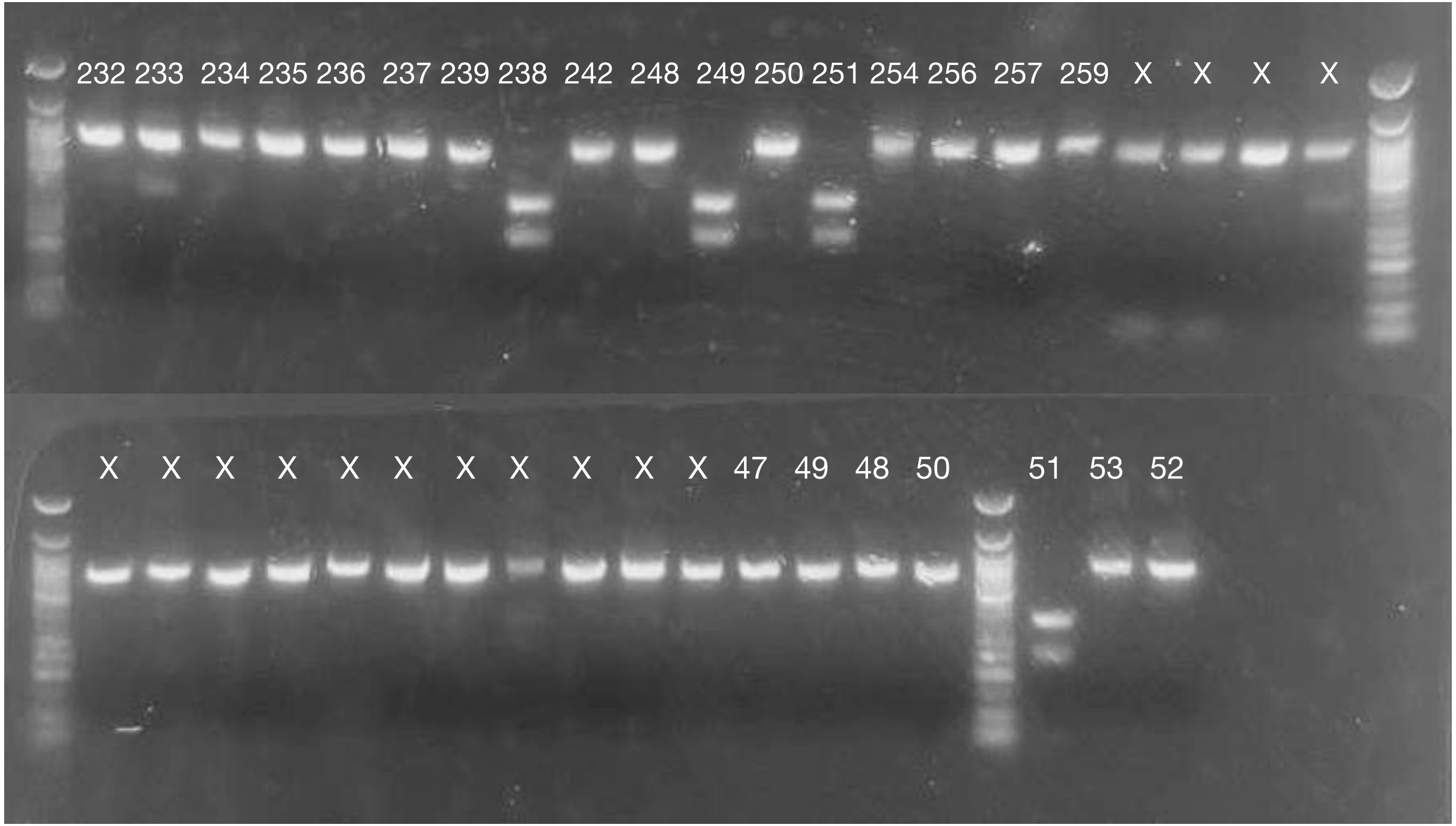

Gel 32
XhoI
23 Feb 26

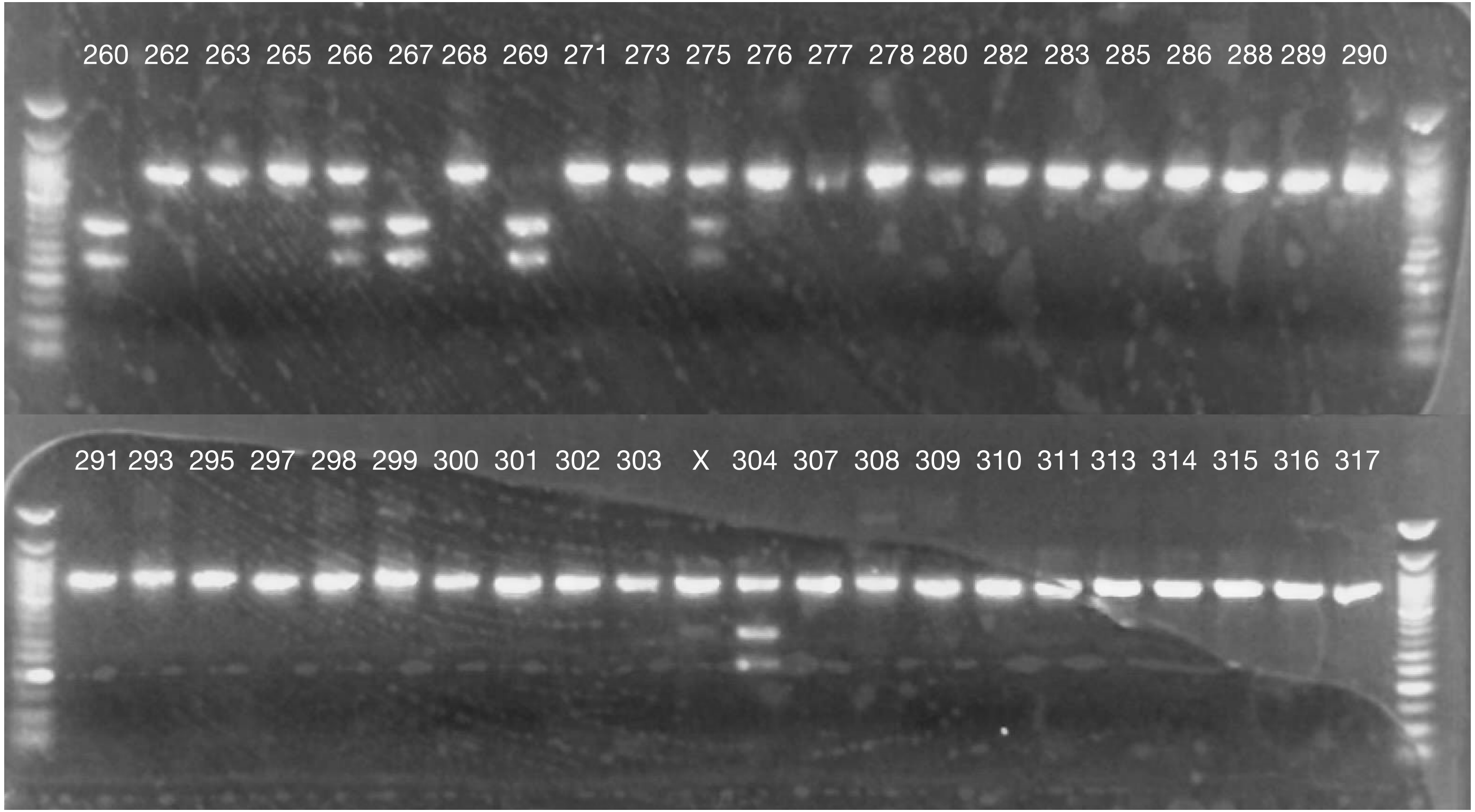

Gel 33
XhoI
23 Feb 26

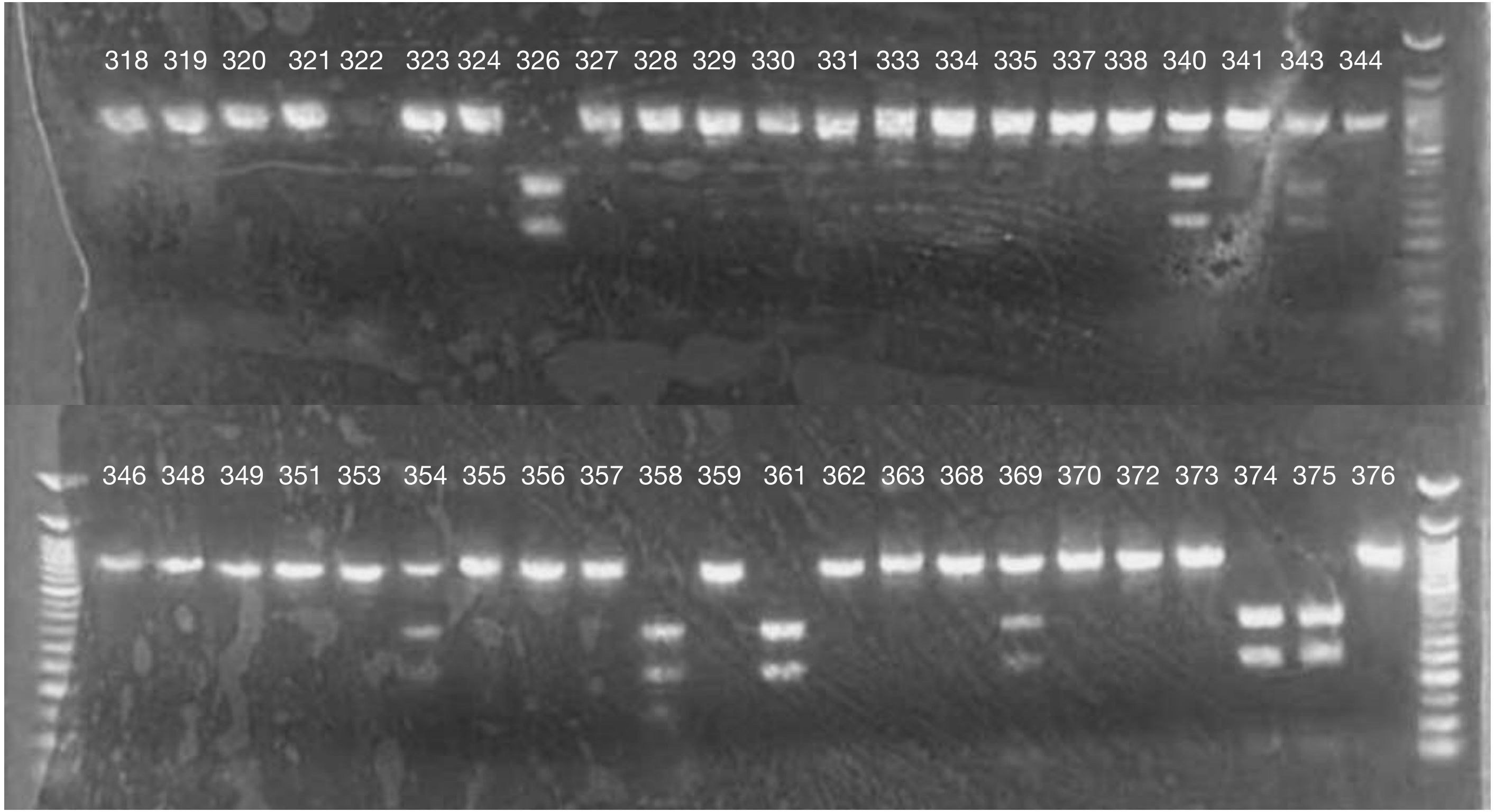

Gel 34
XhoI
23 Feb 26

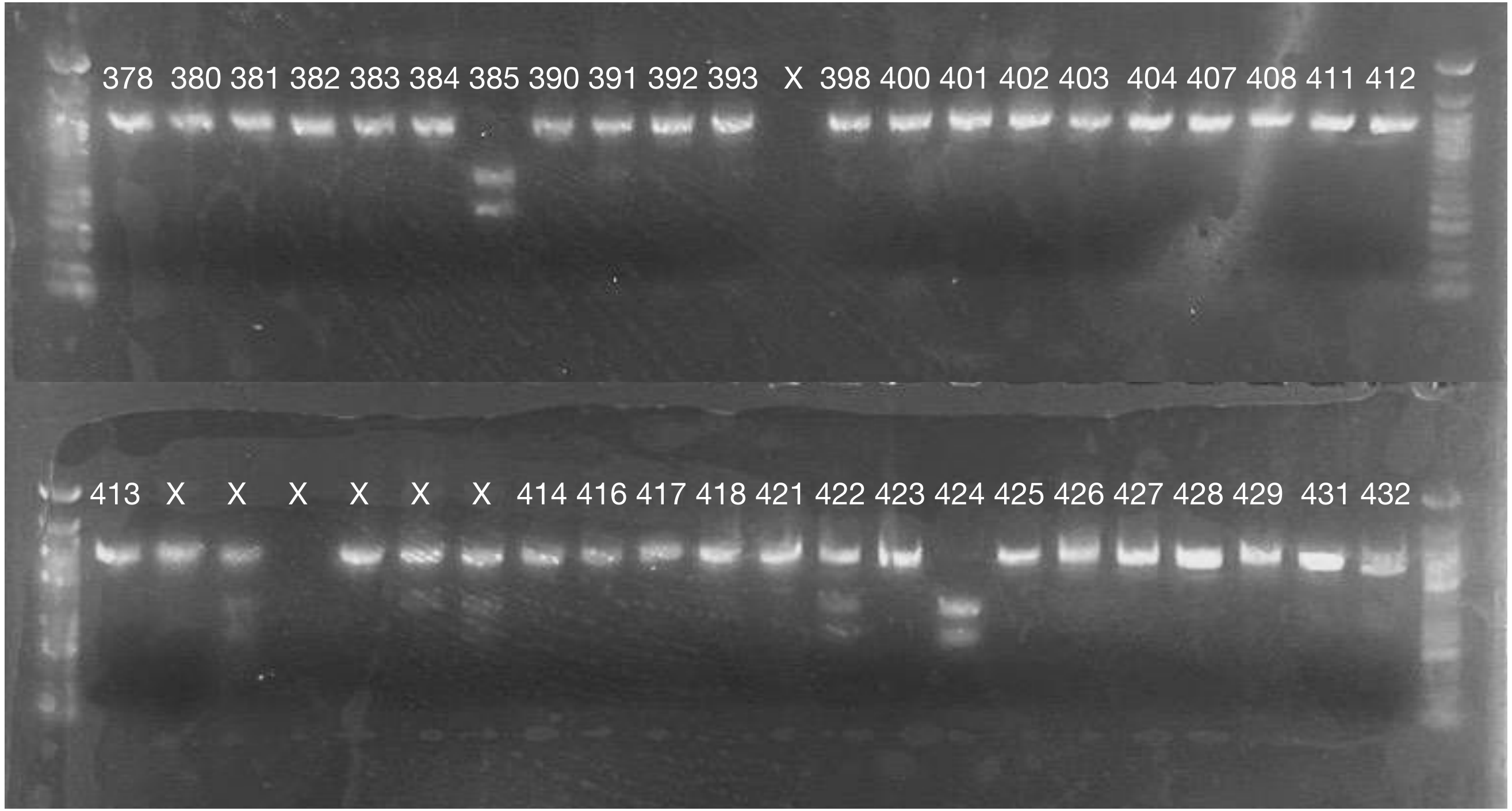

Gel 35
XhoI
23 Feb 26

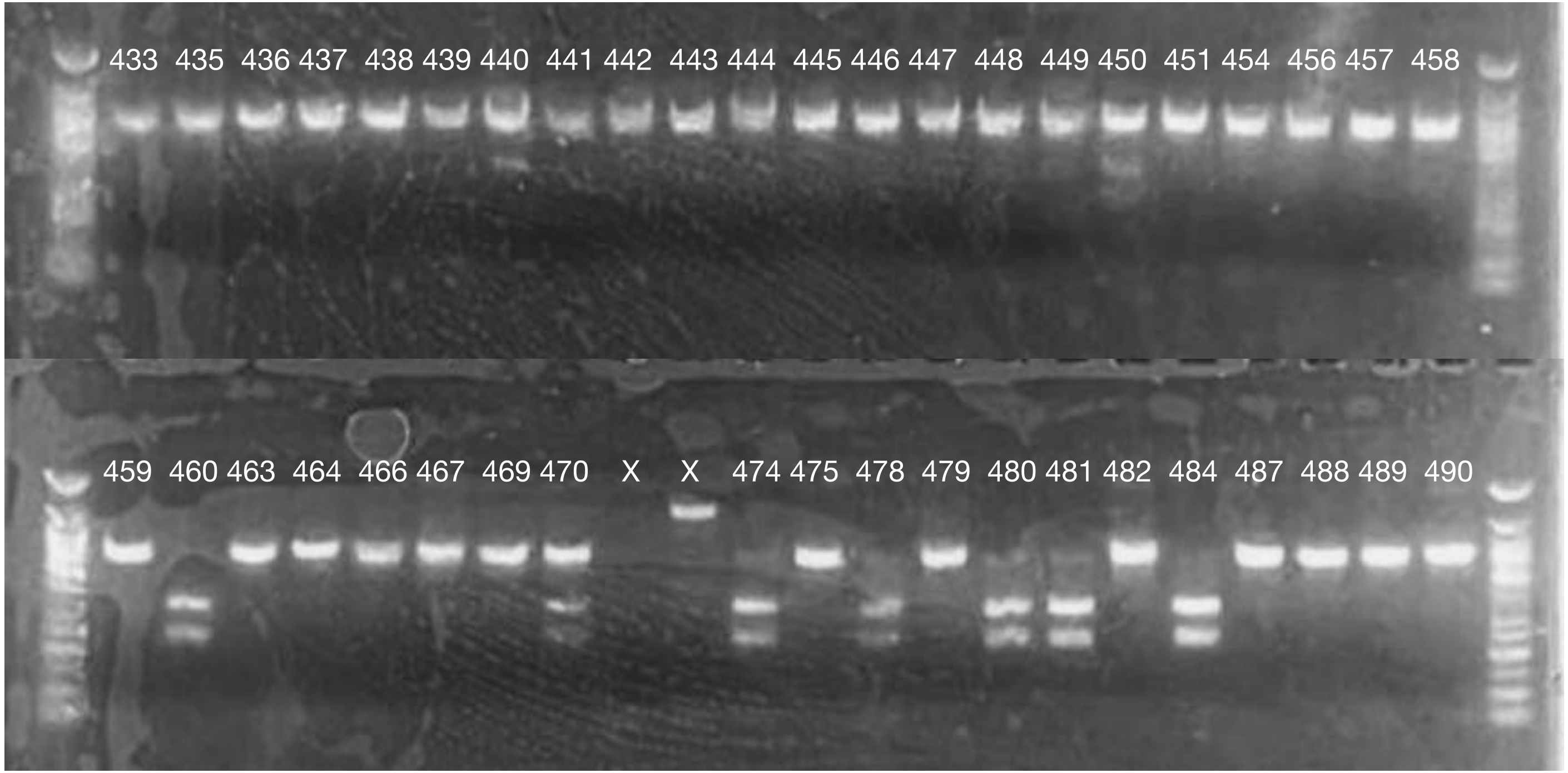

Gel 36
XhoI
23 Feb 26

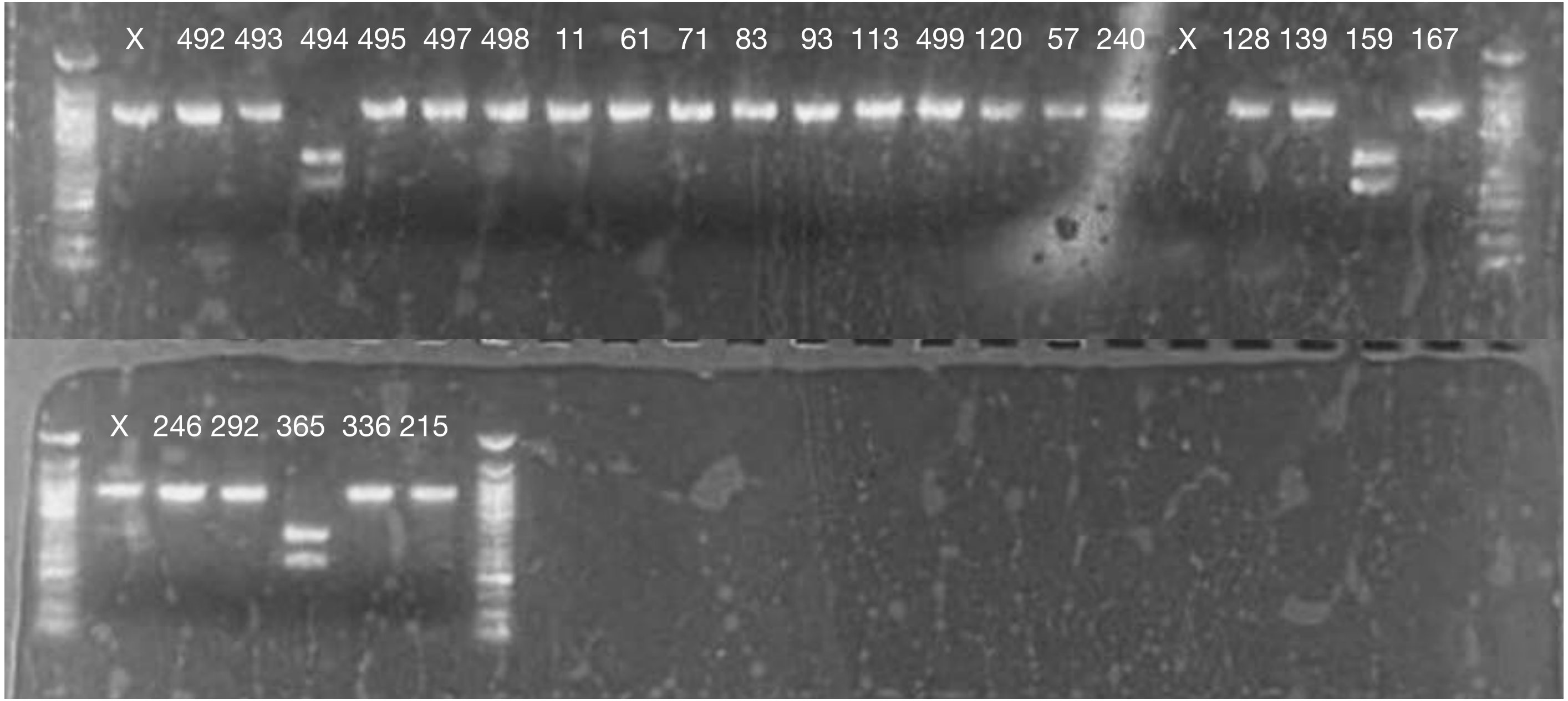

Gel 37
EcoRV-HF, AciI-BseYI (477)
3 Mar 26

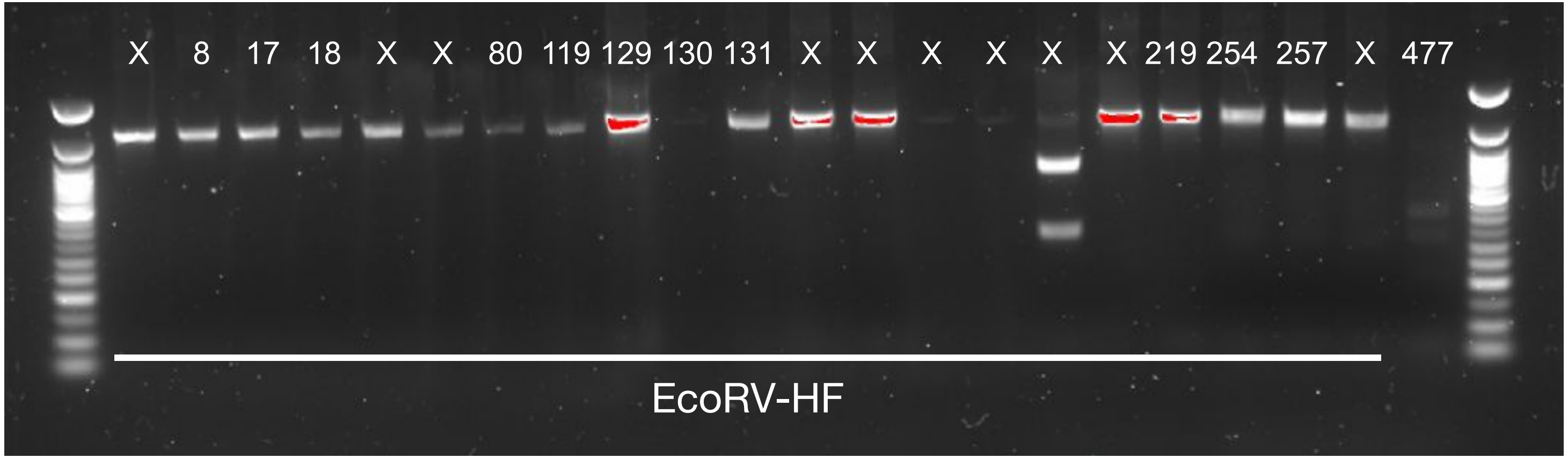

Gel 40
XhoI
8 Mar 26

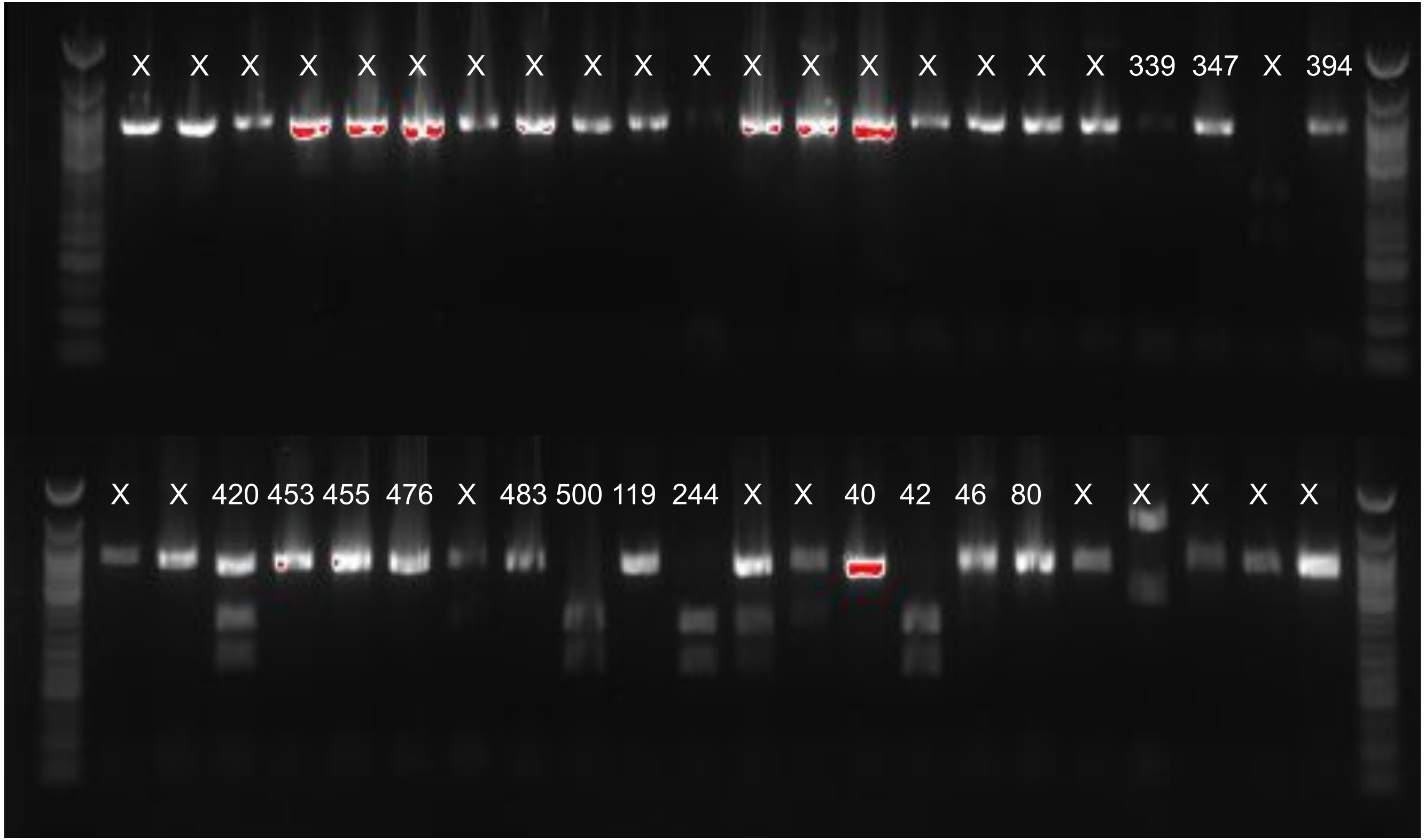

Gel 41
XhoI
11 Mar 26

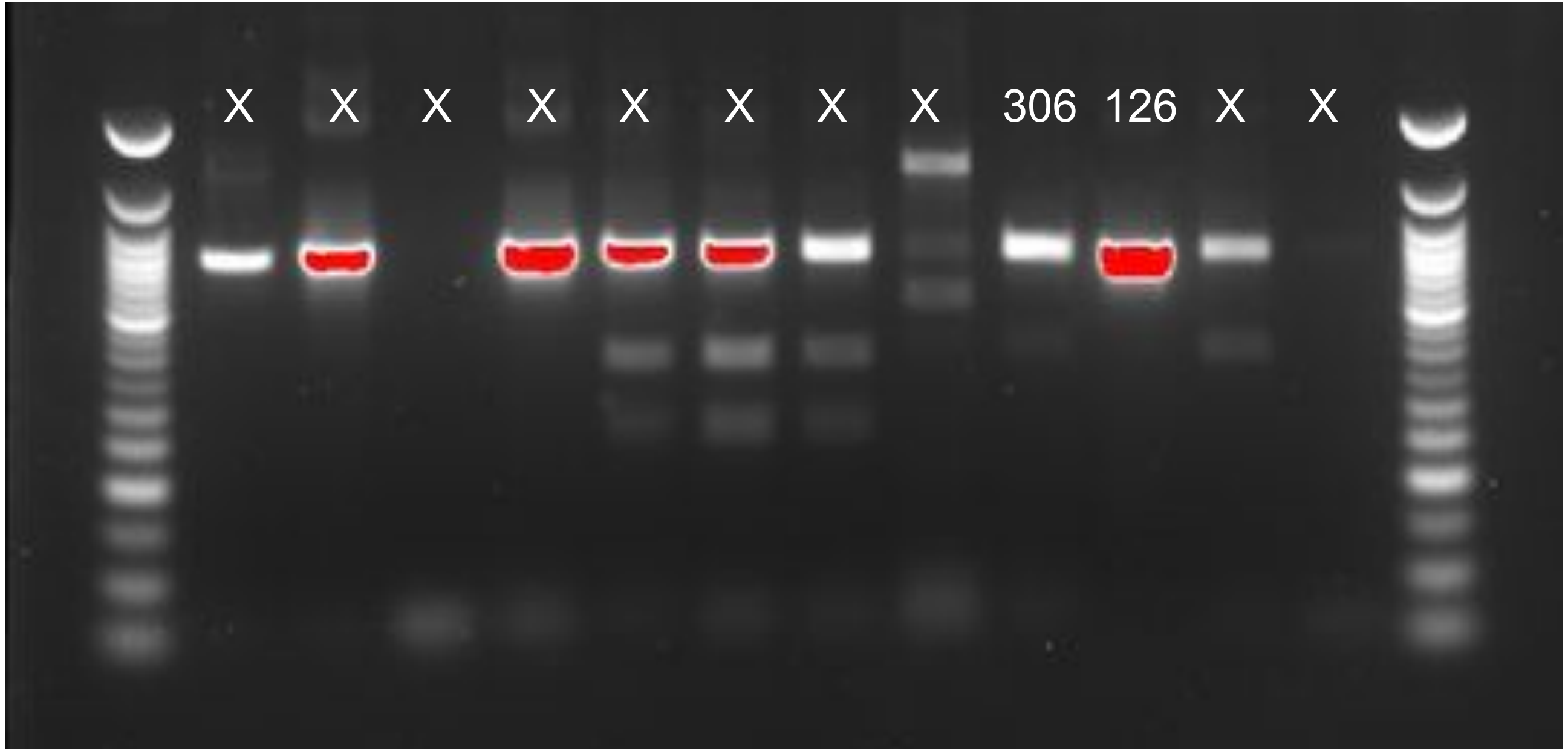

Gel 42
NlaIV
11 Mar 26

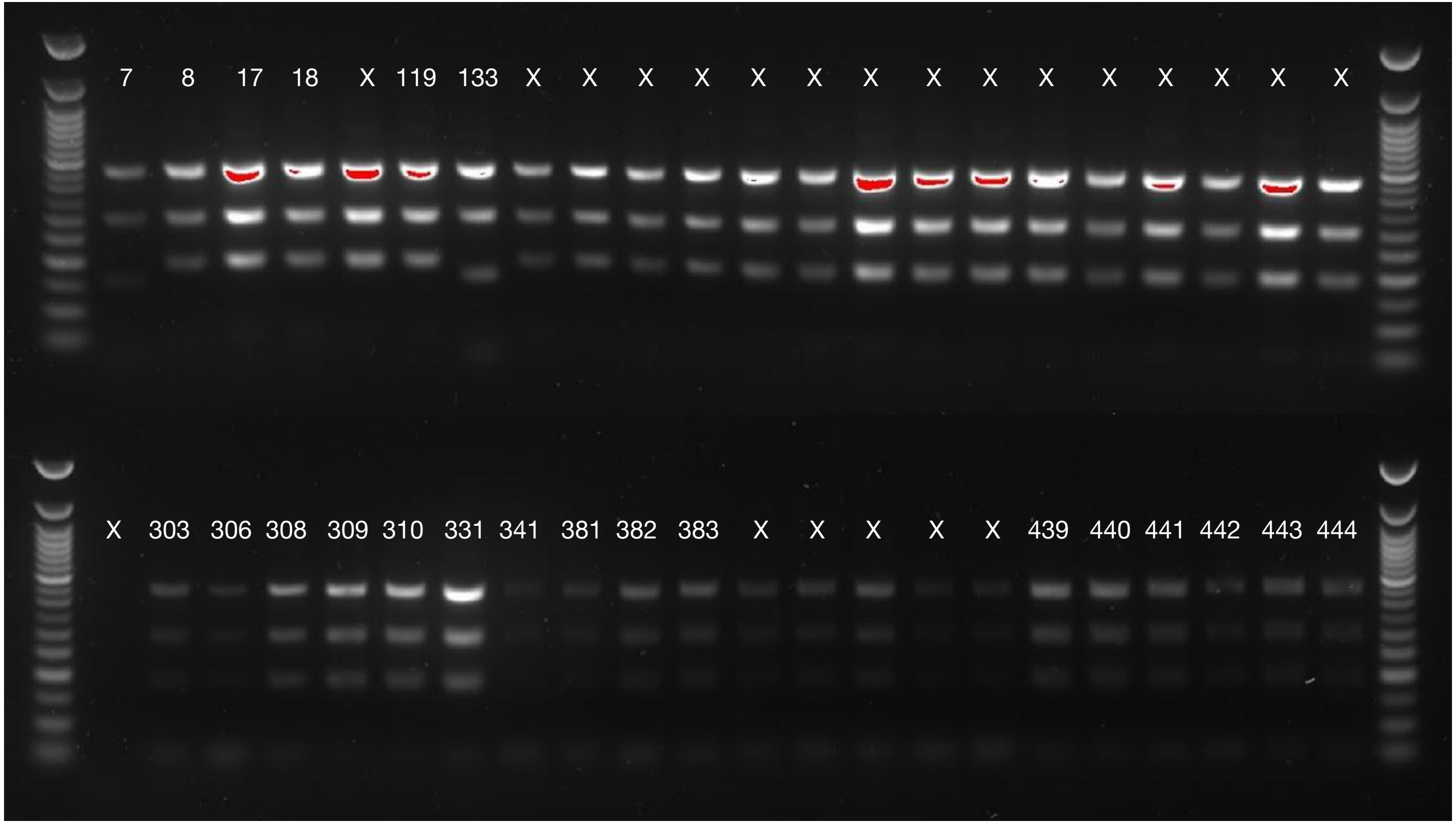

Gel 43
NlaIV
XhoI (116, 159)
AciI-BseYI (207)
11 Mar 26

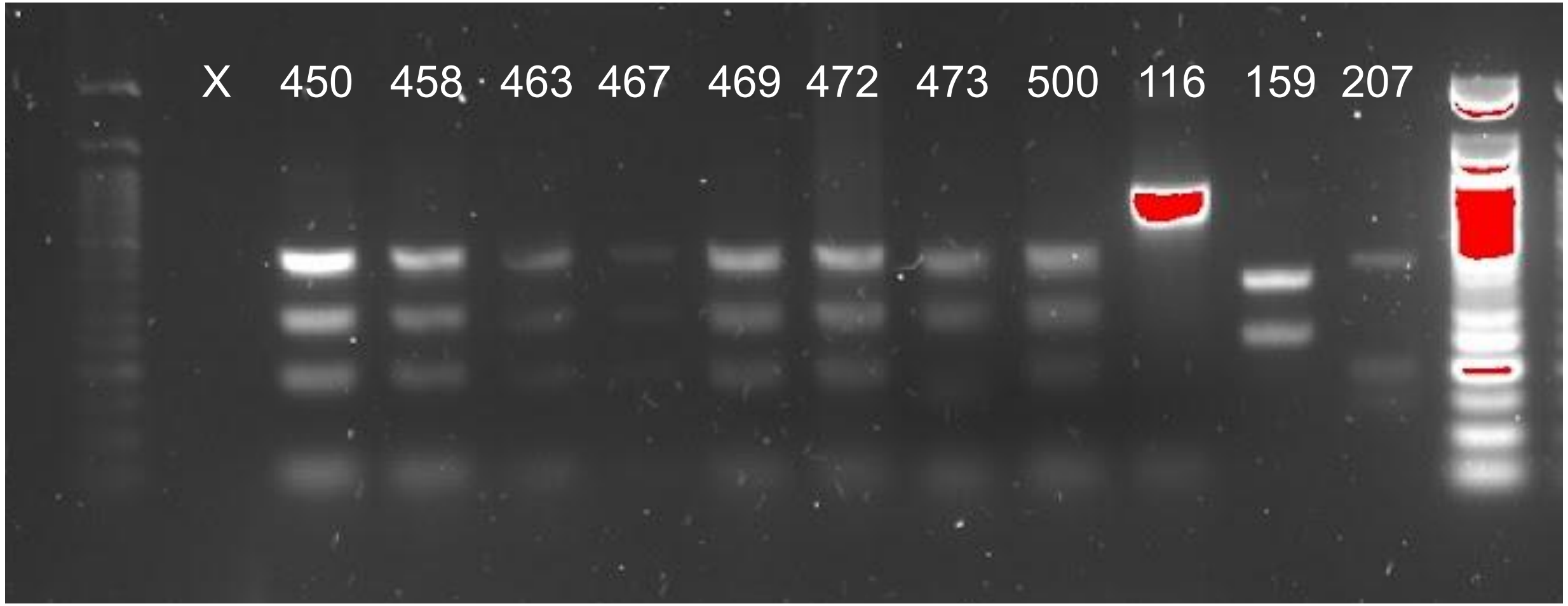

Gel 44
NlaIV, EcoRV-HF
13 Mar 26

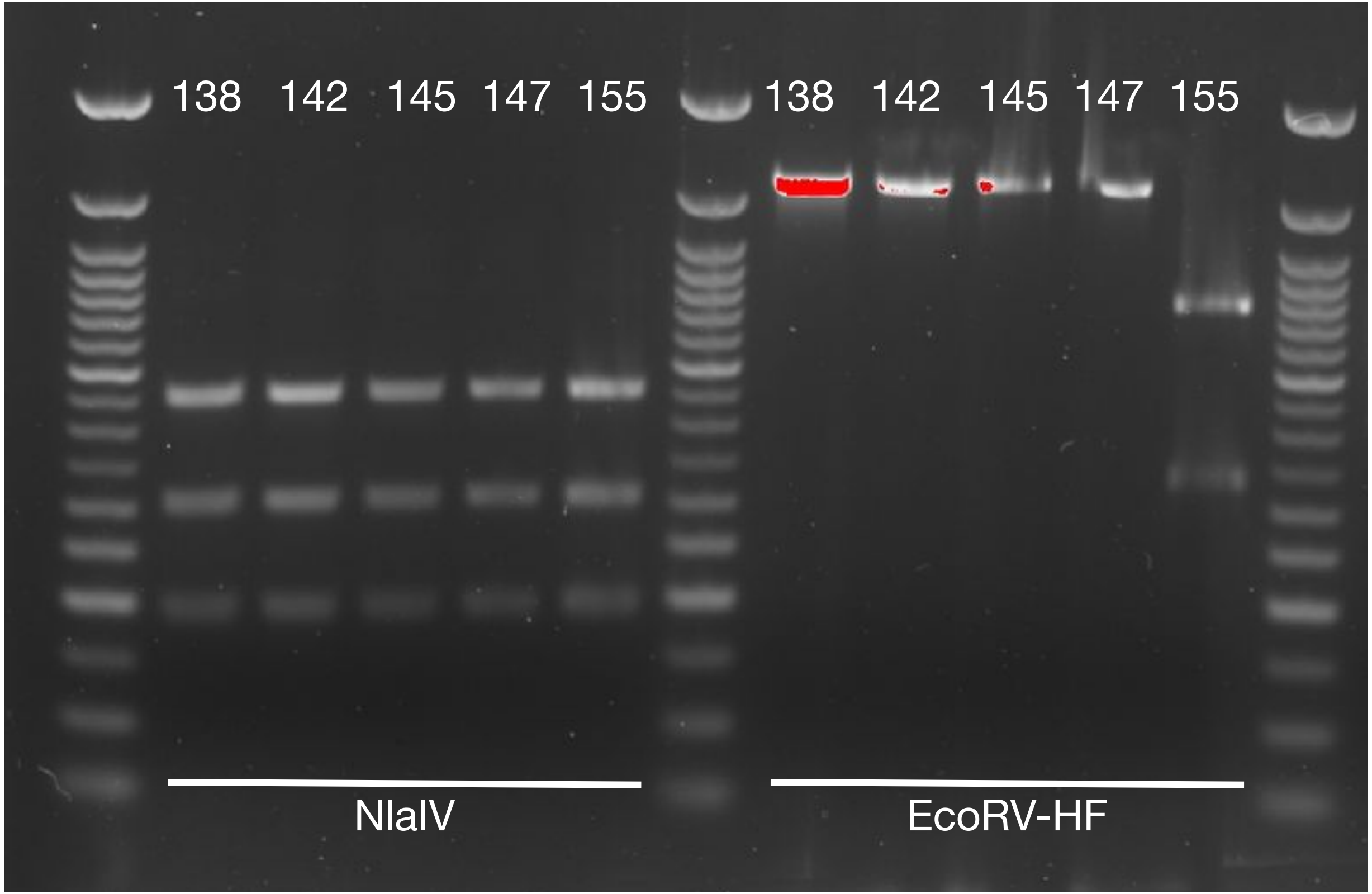

Supplement: Supplementary file 2 [file ECE3-16-e74505-s003.pdf]
