# Supplementary material for: The Role of Light in Mediating Physiological Stress in Small Corals Exposed to Elevated Temperature
Source: Ecol Evol. 2026 Nov 12;16(11):e74505. doi: 10.1002/ece3.74505 (PMC13756585; doi:10.1002/ece3.74505)
Supplement: Supplementary file 3 [file ECE3-16-e74505-s002.docx]

**SUPPLEMENTARY MATERIAL 1**

**The role of light in mediating physiological stress in small corals exposed to elevated temperature**

Edmunds PJ, Scafidi KC, Cohn F, Layagala K, Sims JA, Correa AMS, Burgess SC

Fig. S1. Photographs of representative colonies of *Pocillopora* spp. collected from the back reef of Moorea to support Trial 1 (A) and Trial 2 (B).

Fig. S2. Log_10_ relationship between area (cm^2^) and biomass (mg) for all *Pocillopora* spp*.* in Trial 1 (*P. meandrina*, n = 12; *P. grandis*, n = 1; *P. tuahiniensis*, n = 2) and Trial 2 (*P. meandrina*, n = 8; *P.* cf. *effusa*, n = 3; *P. grandis*, n = 3; *P. verrucosa*, n = 2, no ID, n = 2). Fitted lines and ribbons represent the mean and 95% confidence interval, respectively, estimated from a linear model with interactive effects of trial and area. Refer to Fig. 2 for results from *P. meandrina* alone. Although based on a small sample size, biomass in the “other” *Pocillopora* followed the same trend as for *P. meandrina*, with mean values 16% higher in Trial 1 (9.6 ± 1.8 mg cm^-2^, n = 3) versus Trial 2 (8.3 ± 1.4 mg cm^-2^, n = 10).

Fig. S3. Photophysiological performance of all *Pocillopora* spp. incubated for 11 days in combinations of two temperatures and six light levels in two trials. A randomly selected subset of corals was analyzed for photophysiology at each time point. For measurements on day 11: Trial 1 included 40 *P. meandrina*, 1 *P. acuta*, 5 *P. grandis*, 4 *P. tuahiniensis*, 1 *P. verrucosa*, and 4 no ID; Trial 2 includes 37 *P. meandrina*, 2 *P. acuta*, *4 P.* cf. *effusa*, 30 *P. grandis*, 22 *P. tuahiniensis*, 6 *P. verrucosa*, and 7 no ID. Two response variables were measured: A) minimum fluorescence yield (F_o_), and B) maximum photochemical efficiency of open RCIIs (F_v_/F_m_). The mean (± SE) maximum irradiance at 3-m depth (the collection depth for the corals) was 1,667 ± 9 µmol photons m^-2^ s^-1^ (n = 1,368 days, 19 May 2021 to 29 April 2025), as calculated from K_dPAR_ at 17 m depth on the fore reef of Moorea. Points show the mean ± SE for each tank (n = 2–6 by tank). Lines and shading show the fitted values and 95% CI from a linear mixed effects model with fixed effects of temperature and light, and random effects of trial and tank. On day 11, there were no interactive effects of temperature and light on F_o_ ($\chi^{2}$= 1.018, df = 2, p = 0.601), but F_o_ declined curvi-linearly with light ($\chi^{2}$= 18.779, df = 2, p < 0.001) and was similar in both temperatures ($\chi^{2}$= 0.248, df = 1, p = 0.618). F_v_/F_m_ was significantly affected by the interaction of temperature with light ($\chi^{2}$= 4.648, df = 1, p = 0.031).

Fig. S4. Maximum photochemical efficiency of open RCIIs (F_v_/F_m_) of *Pocillopora* spp. at 28°C (A) and 31°C (B), together with the frequency distribution of daily integrated light at the collection depth from 19 May 2021 to 29 April 2025 (C). Fitted lines and ribbons in A and B represent the mean and 95% CI estimated from a linear effects model with fixed effects of day, temperature and light (as a continuous variable), with tank and trial as random effects. Refer to Fig. 4 for results for *Pocillopora* *meandrina*. F_v_/F_m_ was affected by the interaction of day, temperature, and light ($\chi^{2}$= 28.166, df = 10, p = 0.002, Supplementary Material 1, Table S3). At 31°C, F_v_/F_m_ declined most rapidly at the highest light level, which commonly was reached at 3-m depth.

Fig. S5. The effect of light on the probability of bleaching (rank 1+2), and partial or full mortality (rank 2+3) in small *Pocillopora* *meandrina* by days 8 (A and B) and 11 (C and D) at 28°C or 31°C and six light levels (n = 8-16 per tank) (day 14 displayed in Fig. 5). Lines and shading show the fitted values and 95% CI, respectively, from a binomial linear mixed effects plot with fixed affects of temperature and light and tank as a random effect.

Fig. S6. The effect of light on: a) the probability of bleaching (rank 1+2), and b) partial or full mortality (rank 2+3) in all small *Pocillopora* spp. by day 14 under 28°C or 31°C and six light levels (n = 12-18 per tank). Rugs on upper and lower side of plot frames show records by coral. Lines and shading show the fitted values and 95% CI, respectively, from a binomial linear mixed effects model with fixed effects of temperature and light and tank as a random effect). The probability of bleaching (condition rank 1+2) by day 14 increased with light ($\chi^{2}$= 27.364, df = 1, p < 0.001), but was unaffected by temperature ($\chi^{2}$= 1.785, df = 1, p = 0.182). The odds of bleaching rose by 81.56% (53.03% – 115.41%, 95% CI) for every 100 μmol photons m^-2^ s^-1^ increase in light. There was no interactive effect of temperature and light on the probability of bleaching after 14 days ($\chi^{2}$= 0.159, df = 1, p = 0.690). The probability of partial or full mortality (condition rank 2+3) by day 14 increased with light ($\chi^{2}$ = 16.429, df = 1, p < 0.001) and decreased with temperature ($\chi^{2}$= 14.739, df = 1, p < 0.001). The odds of partial or full mortality by day 14 rose by 27.22% (13.17 - 43.01, 95% CI) for every 100 μmol photons m^-2^ s^-1^ increase in light, and were 341% (2.31 - 8.44, 95% CI) higher at 31°C compared to 28°C (at all light levels). There were no interactive effects of temperature and light on the probability of partial or full mortality by day 14 ($\chi^{2}$= 2.179, df = 1, p = 0.140).

Fig. S7. The effect of light on the probability of bleaching (rank 1+2), and partial or full mortality (rank 2+3) in all small *Pocillopora* spp. by days 8 (A and B) and 11 (C and D) at 28°C or 31°C and six light levels (n = 12–18 per tank) (day 14 displayed in Fig. 5). Lines and shading show the fitted values and 95% CI, respectively, from a binomial linear mixed effects model with fixed effects of temperature and light and tank as a random effect).

Table S1. Statistical contrast of log_10_(biomass) of all *Pocillopora* spp. (Fig. S2) between Trials with Log_10_ (area) as the covariate. Refer to Fig. 2 and Table 3 for the results for *P.* *meandrina*.

| Model structure | ΔAIC | Effect | 𝝌 ^2^ | df | P |
| --- | --- | --- | --- | --- | --- |
| Area + Trial | 0.00 | Trial  Area | 14.530  42.297 | 1  1 | < 0.001  < 0.001 |
| Area * Trial | 0.14 | Area × Trial | 2.593 | 1 | 0.107 |

Table S2. Results of model selection for analysis of *Pocillopora meandrina* using LME or GLMM to test for variation in F_o_, F_v_/F_m_, and condition rank as a function of temperature and light with trial and tanks as random effects. Results for F_o_ and F_v_/F_m_ presented for day 11, day 1 through 14 for F_v_/F_m_, and for day 14 for condition ranks; refer to Table S3 for results from other days. Analyses for bleaching contrasts condition rank 0 vs (1 + 2), severe bleaching contrasts condition rank 1 vs 2, and mortality contrasts condition rank 0+1 vs 2+3. DV = dependent variable, ΔAIC_c_ = change relative to the lowest value of AIC_c_, K = number of parameters. Best supported models (with lowest AICc or those differing by ≲ 2.0) in bold, with significance of the terms within those models reported in Table 4.

| DV |  | Model structure | ΔAIC_c_ | K |
| --- | --- | --- | --- | --- |
| F_o_ (day 11) |  | **light** | **0.0** | **5** |
|  |  | **poly(light, 2)** | **0.08** | **6** |
|  |  | **light + temperature** | **1.75** | **6** |
|  |  | poly(light, 2) + temperature | 2.32 | 7 |
|  |  | light × temperature | 4.11 | 7 |
|  |  | 1 (grand mean) | 7.05 | 4 |
|  |  | poly(light, 2) × temperature | 7.13 | 9 |
|  |  | temperature | 9.08 | 5 |
| F_v_/F_m_ (day 11) |  | **light** × **temperature** | **0.00** | **7** |
|  |  | **poly(light, 2)** × **temperature** | **0.89** | **9** |
|  |  | light + temp | 8.50 | 6 |
|  |  | poly(light, 2) + temperature | 9.18 | 7 |
|  |  | light | 11.93 | 4 |
|  |  | poly(light, 2) | 14.58 | 6 |
|  |  | temperature | 21.81 | 5 |
|  |  | 1 (grand mean) | 22.92 | 4 |
| F_v_/F_m_ |  | **day** × **light** × **temperature** | **0.00** | **11** |
| (day 1 through 14) |  | **poly(day, 2)** × **poly(light,2)** × **temp** | **1.15** | **21** |
|  |  | poly(day, 2) × light × temp | 3.21 | 15 |
|  |  | day + light + temperature + day:light + day:temperature + light:temp | 13.83 | 10 |
|  |  | day + poly(light,2) + temperature + day:poly(light,2) + day:temperature + poly(light,2):temp | 16.59 | 13 |
|  |  | poly(day,2) + poly(light,2)+ temperature + poly(day,2):poly(light, 2) + poly(day,2):temperature + poly(light,2):temperature | 18.59 | 17 |
| Condition rank: bleaching |  | **light** | **0.00** | **4** |
| (0 vs 1+2), day 14 |  | **light + temperature** | **1.50** | **5** |
|  |  | light + temperature + light:temperature | 3.54 | 6 |
|  |  | 1 (grand mean) | 19.50 | 3 |
|  |  | temperature | 21.50 | 4 |
| Condition rank: mortality |  | **light + temperature + light:temperature** | **0.00** | **6** |
| (partial and complete) |  | **light + temperature** | **0.20** | **5** |
| (0+1 vs 2+3), day 14 |  | temperature | 12.37 | 4 |
|  |  | light | 12.81 | 4 |
|  |  | 1 (grand mean) | 19.00 | 3 |

Table S3. Results of model selection for analysis of all *Pocillopora* spp. using LME or GLMM to test for variation in F_o_, F_v_/F_m_, and condition rank as a function of temperature and light with tank as a random effect. Results for F_o_ and F_v_/F_m_ presented for day 11, all days for F_v_/F_m_ at the three highest light levels, and for day 14 for condition ranks. Analyses for bleaching contrasts condition rank 0 vs (1 + 2) and mortality contrasts condition rank 0+1 vs 2+3. DV = dependent variable, ΔAIC_c_ = change relative to the lowest value of AIC_c_, K = number of parameters, and best supported models (with lowest AIC_c_ or those differing by ≲ 2.0) in bold.

| DV | Model | Model structure | ΔAIC_c_ | K |
| --- | --- | --- | --- | --- |
| F_o_ (day 11) | LME | **poly(light, 2)** | **0.00** | **6** |
|  |  | **poly(light, 2) + temperature** | **1.92** | **7** |
|  |  | light | 2.46 | 5 |
|  |  | light + temperature | 4.70 | 6 |
|  |  | poly(light, 2) × temperature | 5.35 | 9 |
|  |  | light × temperature | 6.94 | 7 |
| F_v_/F_m_ (day 11) | LME | **poly(light, 2)** × **temperature** | **0.00** | 9 |
|  |  | **light** × **temperature** | **1.85** | 7 |
|  |  | poly(light, 2) + temperature | 4.53 | 7 |
|  |  | light + temperature | 5.27 | 6 |
|  |  | light | 11.74 | 4 |
|  |  | poly(light, 2) | 14.60 | 6 |
|  |  | temperature | 15.37 | 5 |
|  |  | 1 (grand mean) | 19.30 | 4 |
| F_v_/F_m_ for 80%, 90%,100% light | LME | **poly(day, 2)** × **poly(light,2)** × **temp** | **0** | 21 |
| (across all days) |  | **day** × **light** × **temperature** | 4.92 | 11 |
|  |  | **day** × **poly(light, 2)** × **temperature** | 22.2 | 15 |
|  |  | poly(day, 2) + poly(light, 2) + temperature + poly(day, 2): poly(light, 2) + poly(day,2) :temperature + poly(light, 2):temperature | 22.77 | 17 |
|  |  | day + light + temperature + day:light + day:temperature + light:temp | 32.12 | 10 |
|  |  | day + poly(light,2 ) + temperature + day:poly(light, 2) + day:temperature + poly(light, 2):temp | 32.82 | 13 |
| Condition rank: bleaching |  | **light** | **0.00** | **4** |
| (0 vs 1+2), day 14 |  | **light + temperature** | **0.26** | **5** |
|  |  | light + temperature + light:temperature | 2.16 | 6 |
|  |  | 1 (grand mean) | 24.07 | 3 |
|  |  | temperature | 25.58 | 4 |
| Condition rank: mortality |  | **light + temperature + light:temperature** | **0.00** | **6** |
| (partial and complete) |  | **light + temperature** | **0.12** | **5** |
| (0+1 vs 2+3), day 14 |  | light | 12.81 | 4 |
|  |  | temperature | 14.50 | 4 |
|  |  | 1 (grand mean) | 20.26 | 3 |
